# Semaglutide ameliorates pressure overload-induced cardiac hypertrophy by improving cardiac mitophagy to suppress the activation of NLRP3 inflammasome

**DOI:** 10.1038/s41598-024-62465-6

**Published:** 2024-05-23

**Authors:** Wenxiu He, Jiahe Wei, Xing Liu, Zhongyin Zhang, Rongjie Huang, Zhiyuan Jiang

**Affiliations:** Department of Cardiology, First Affiliated Hospital, Guangxi Medical University, 6 Shuangyong Road, Qingxiu District, Nanning, 530021 China

**Keywords:** Glucagon-like peptide-1 receptor agonist, Semaglutide, Pressure overload, Cardiac hypertrophy, Mitophagy, NLRP3 inflammasome, Cardiology, Medical research

## Abstract

Pathological cardiac hypertrophy is an important cause of heart failure(HF). Recent studies reveal that glucagon-like peptide-1 receptor (GLP1R) agonists can improve mortality and left ventricular ejection fraction in the patients with type 2 diabetes and HF. The present study aims to investigate whether semaglutide, a long-acting GLP1R agonist, can ameliorate cardiac hypertrophy induced by pressure overload, and explore the potential mechanism. The rats were performed transverse aortic constriction (TAC) to mimic pressure overload model. The rats were divided into four groups including Sham, TAC, TAC + semaglutide, and TAC + semaglutide + HCQ (hydroxychloroquine, an inhibitor of mitophagy). The rats in each experimental group received their respective interventions for 4 weeks. The parameters of left ventricular hypertrophy(LVH) were measured by echocardiography, Hematoxylin–eosin (HE) staining, western-blot and immunohistochemistry (IHC), respectively. The changes of mitophagy were reflected by detecting cytochrome c oxidase subunit II (COXII), LC3II/LC3I, mitochondria, and autophagosomes. Meanwhile, NLRP3, Caspase-1, and interleukin-18 were detected to evaluate the activation of NLRP3 inflammasome in each group. The results suggest that LVH, impaired mitophagy, and activation of NLRP3 inflammasome were present in TAC rats. Semaglutide significantly reduced LVH, improve mitophagy, and down-regulated NLRP3 inflammatory signal pathway in TAC rats. However, the reversed effect of semaglutide on cardiac hypertrophy was abolished by HCQ, which restored the activation of NLRP3 inflammasome suppressed by improved mitophagy. In conclusion, semaglutide ameliorates the cardiac hypertrophy by improving cardiac mitophagy to suppress the activation of NLRP3 inflammasome. Semaglutide may be a novel potential option for intervention of cardiac hypertrophy induced by pressure overload.

## Introduction

Cardiovascular diseases are the leading cause of death globally^1^. The mortality of heart failure(HF) resulting from various cardiovascular diseases ≈ 50% at 5 years^2^. The onset of HF is typically preceded by cardiac hypertrophy, a compensatory hypertrophy of the cardiac myocytes in response to elevated left ventricular (LV) wall stress. However, the response becomes maladaptive with persistent volume and pressure overload^3^. This pathologically cardiac hypertrophy is an independent predictor of adverse cardiovascular outcomes, such as cardiovascular death, heart failure, myocardial infarction, arrhythmia, and stroke^4–6^.

Hypertension is the most cause of left ventricular hypertrophy (LVH), which increases pressure load of left ventricle. In 2019, the global age-standardized prevalence of hypertension in adults aged 30–79 years was 32% in women and 34% in men^7^. The absolute number of people aged 30–79 years with hypertension doubled from 331 million women and 317 million men in 1990 to 626 million women and 652 million men in 2019 as population growth and ageing^7^. Moreover, hypertension is the single most important risk factor for heart failure, and about 75% of patients with HF have antecedent hypertension^8^. Therefore. It is important to explore the mechanism of LVH induced by pressure overload for prevention and intervention of HF.

Semaglutide is a long-acting glucagon-like peptide-1 receptor (GLP1R) agonist that stimulates insulin secretion, reduces blood glucose levels, and lowers hemoglobin levels. Interestingly, recent evidences indicate that GLP1R agonists can significantly reduce adverse cardiovascular events, renal dysfunction, and the incidence of stroke in the patients with diabetes^9–12^. In addition, some studies have shown that GLP1R agonists can improve mortality, increased left ventricular ejection fraction, and attenuate the level of brain natriuretic peptide in the patients with type 2 diabetes and HF^13–15^. However, the effect of GLP1R agonists on LVH induced by pressure overload is still unclear.

Recently, some studies have demonstrated that impaired mitophagy contributes to cardiac hypertrophy in the condition of pressure overload^16,17^. Meanwhile, mitophagy dysfunction can cause HF by activating innate immune responses, such as activation of NLRP3 inflammasome^18,19^. Additionally, GLP1R agonists have shown anti-inflammatory effects in the cardiovascular diseases^20,21^. Taken together, our present study was aimed to testify whether semaglutide could reverse LVH induced by pressure overload, and whether the effects of semaglutide on LVH were achieved by improving mitophagy dysfunction to suppress the activation of NLRP3 inflammasome.

## Materials and methods

### Animals

Adult male Sprague–Dawley rats weighing 180–220 g were obtained from the Guangxi Medical University Laboratory Animal Centre. The experimental protocols were performed in accordance with the National Institutes of Health Guide for the Care and Use of Laboratory Animals and were approved by Animal Ethics Committee of Guangxi Medical University. Additionally, we confirm that all aspects of the study are reported in accordance with the ARRIVE guidelines to ensure high-quality and transparent reporting of animal research.

### TAC model

The model of pressure overload-induced LVH was made by transverse aortic constriction (TAC). After an 8-h fasting period, the rats were anesthetized with a 10% pentobarbital sodium solution administered with 25 mg/kg intraperitoneally. After the rat was anesthetized fully, a tracheal intubation was performed, and a ventilator was used to assist ventilation of the rat. The left third rib of rat was transected to open thoracic cavity. Then, the aortic arch was adequately exposed by carefully separating the surrounding connective tissue. Subsequently, a 4–0 nylon monofilament suture was placed underneath the aortic arch between brachiocephalic trunk and left common carotid artery, and a 27G puncture needle was positioned parallel to the aortic arch and ligated to the aorta using a nylon monofilament suture. Lastly, the needle was removed, and the thoracic cavity was closed layer by layer. Erythromycin ointment was applied to the surgical wound after the procedure. In Sham group, the same surgical procedure was performed, but no ligation of the aorta was carried out.

### Intervention of animals

The rats were divided into four groups including Sham group, TAC group, TAC + semaglutide group, TAC + semaglutide + HCQ (hydroxychloroquine) group. Initially, we aimed to include six rats in each group. However, due to mortality associated with the TAC procedure, we adjusted the number of rats in the TAC, TAC + semaglutide, and TAC + semaglutide + hydroxychloroquine (HCQ) groups. Consequently, the final composition was 9 rats in the TAC group, 8 rats rats in TAC + semaglutide group, and 8 rats in TAC + semaglutide + HCQ group, all of whom underwent TAC surgery. Two days after operation, 1 rat from each group succumbed. Therefore, there were 6 rats in Sham group, 8 rats in TAC group, 7 rats in TAC + SMGLT group, and 7 rats in TAC + SMGLT + HCQ group. To better match with Sham group including 6 rats, 6 rats were randomly selected to perform subsequent analyses in TAC group, TAC + semaglutide group, and TAC + semaglutide + HCQ group. The rats in Sham group and TAC group were not administration of drugs. The rats in TAC + semaglutide group were administrated with semaglutide (1 mg/kg) (NovoNordiskA/S, Denmark) once a week, subcutaneously^22^. The rats in TAC + semaglutide + HCQ group were administrated with semaglutide (1 mg/kg) once a week, and HCQ (catalog No. B4874, APExBIO, USA), an inhibitor of mitophagy, 50 mg/kg daily, intraperitoneally^23^. Four weeks after the intervention, each rat in four groups was performed an echocardiography, and then their hearts were collected to perform histological analysis, immunohistochemistry (IHC) analysis, ultrastructure analysis, and protein analysis.

### Echocardiography

After the rat was anesthetized with 20 mg/kg of 10% pentobarbital sodium solution completely, it was fixed in a supine position, and removed thoracic and abdominal hairs. Echocardiography was performed by using an Animal Digital Ultrasound Imaging System (Mylab Sixvet, Saote, Italy) with a 30-MHz scan probe. The speed of the M-shaped diagram is 15 cm/s. Left ventricular posterior wall thickness (LVPWT) and inter-ventricular septum thickness (IVST) were measured from 2D long-axis views under M-mode tracings at the level of the left ventricular posterior papillary muscles and end-diastolic phase of left ventricle. Left ventricular end systolic diameter (LVESD), and left ventricular end diastolic diameter (LVEDD) were measured from 2D long-axis views under M-mode tracings at the level of the left ventricular tendons. FS was calculated by the following formula: (LVEDD-LVESD/LVEDD) × 100%^24–26^. Left ventricular end diastolic volume(LVEDV) was calculated by the following formula: [7/(2.4 + LVEDD)] × LVEDD^3^, and Left ventricular end systolic volume(LVESV) was calculated by the following formula: [7/(2.4 + LVESD)] × LVESD^3^^27^. Left ventricular ejection fraction (LVEF) was calculated by the following formula: (LVEDV − LVESV)/LVEDV × 100%^25^. Left ventricular mass index (LVMI) was calculated by the following formula: {0.8 × 1.04 × [(IVST + LPWT + LVEDD)^3^ − LVEDD^3^] + 0.6}/weight^28^. Six rats in each group were perform the measurement of echocardiography. All measurements were obtained over three consecutive cardiac cycles.

### Hematoxylin and eosin staining

The Hematoxylin and eosin (HE) staining was performed as previous report^29^. Briefly, paraffin-embedded cardiac tissues sections were routinely dewaxed in xylene. The sections were stained in the hematoxylin staining solution for 4 min. One hour after running water washing, the sections were differentiated with 0.3% acid alcohol and washed again with distilled water. Then, the sections were stained in eosin solution for 90 s. Subsequently, the sections were dehydrated in gradient ethanol (90%–70%), made transparent with xylene. The sections of whole heart from three rats in each group were made to evaluate the LV wall thickness, and the sections of LV tissues from six rats in each group were made to observe inflammatory cells infiltration. Lastly, the sections were observed under the microscope.

### Immunohistochemistry

Paraffin-embedded tissues sections were routinely dewaxed in xylene, then dehydrated in gradient ethanol (100–90%–80%–75%–50%), followed by triple washing with phosphate-buffered saline. Antigen retrieval was performed by heat mediation in sodium citrate buffer (pH 6.0)^30^. The sections were blocked for 1 h at room temperature with 5% normal goat serum and stained with anti-atrial natriuretic peptide (ANP) antibody (Servicebio, Wuhan, China) diluted 1: 200, anti-myosin heavy chain 7(MYH7) antibody (Proteintech Group, Inc, Wuhan, China) diluted 1: 200, anti-cytochrome c oxidase subunit II (COX II) antibody (Cell Signaling Technology, Inc, Boston, USA) diluted 1: 1000, anti-NLRP3 antibody diluted (ABclonal, Wuhan, China) 1: 200, and anti- interleukin-18 (IL-18) antibody (Servicebio, Wuhan, China) diluted 1: 1000 overnight at 4 °C. The next day, the sections were stained with horseradish peroxidase-conjugated goat anti-rabbit IgG (Servicebio, Wuhan, China) diluted 1: 300 for 1 h at room temperature. The signals were then visualized by DAB kit (Servicebio, Wuhan, China) and observed under microscope. The LV tissues from six rats in each group were used to detect the expression of ANP, MYH7, and COX II by Immunohistochemistry. The Image J software (version 1.53 K, National Institutes of Health, USA) was used to determine the average optical density of positive tissue in each field.

### Transmission electron microscope

The mitochondria and autophagosome were observed using a transmission electron microscope (TEM). Cardiac tissues were cut into 1 × 1 × 1 mm pieces and fixed with 2.5% glutaraldehyde for 4 h. The samples were then washed with 0.1 M phosphate-buffered saline (PBS) and postfixed with a 1% osmium tetroxide solution for 2 h. After that, the samples were dehydrated using an ethanol gradient series (100–90–80–75–50%) and 100% acetone. They were then immersed in propylene oxide and embedded in epoxy resin at 60 °C. Ultrathin sections were obtained using a Leica EM UC6rt ultramicrotome (Leica Microsystems, Inc., Wetzlar, Germany) and were stained with 0.1% uranyl acetate and 3% lead citrate solution. The sections were observed and photographed^31^. The LV tissues from six rats in each group were used to observe mitochondria and autophagosomes by TEM. We randomly selected 1 section from every rat in all four groups to calculate the amount of mitochondria and autophagosomes.

### Western blot analysis

The proteins of LV tissues were extracted, then were stored in a refrigerator at − 80 °C. The concentrations of proteins were determined with a BCA protein assay kit (Beyotime Institute of Biotechnology, Shanghai, China), and the proteins were boiled with 5X SDS-PAGE sample loading buffer (Beyotime Institute of Biotechnology, Shanghai, China). Subsequently, the proteins (20 ~ 80 μg) were separated by 8%, 10%, or 12% sodium dodecyl sulfated-polyacrylamide gel electrophoresis using voltage mode ranging from 80 to 120 V, and then transferred to 0.22 μm of polyvinylidene fluoride membranes (Millipore, Billerica, Mass., USA) using the Mini Trans-Blot electrophoretic transfer cell system (Bio-Rad Laboratories, Inc., Hercules, CA, USA). There were 10 pores in our gels. The protein markers and proteins in Sham group, TAC group, TAC + semaglutide group, and TAC + semaglutide + HCQ were loaded from the first pore to fifth pore in the gels, respectively. After electrophoresis, the gels without loading proteins were cut, then blots including goal proteins were obtained according to protein markers, to be transferred to the membranes.

The membranes were blocked for 1 h at room temperature with 5% nonfat milk in TBST (20 mM Tris–HCl, 0.5 M NaCl, 0.1% Tween 20). After that, the membranes including the goal proteins were incubated with the corresponding antibody including anti-ANP antibody (abcam, Cambridge, MA, USA) diluted 1: 5000, anti-MYH7 antibody (Proteintech Group, Inc, Wuhan, China) diluted 1: 2000, anti-COXII antibody (Cell Signaling Technology, Inc, Boston, USA) diluted 1: 1000, anti-LC3B antibody (Abclonal, Wuhan, China) diluted 1: 2000, anti-GAPDH antibody (abcam, Cambridge, MA, USA) diluted 1: 10,000, anti-NLRP3 antibody (Abclonal, Wuhan, China) diluted 1: 2000, anti-IL-18 antibody (Abclonal, Wuhan, China) diluted 1: 2000, and anti-Caspase-1 antibody (Proteintech Group, Inc, Wuhan, China) diluted 1: 2000 for at least 16 h. Next, these membranes were washed three times with TBST, and were incubate with the goat anti-rabbit IgG antibody (Abclonal, Wuhan, China) diluted 1: 10,000 for 1 h followed by triple washing with TBST. The LV tissues from six rats in each group were used to detect the relative expression of ANP, MYH7, COX II, LC3II/LC3I, NLRP3, IL-18, and Caspase-1 by Western blot. The Protein expression was detected using a FluorChem M System (ProteinSimple, CA, USA), and the quantification was performed using the Image J software (version 1.53 K, National Institutes of Health, USA).

### Statistical analysis

For statistical analysis, the quantitative data with normal distribution were presented as the mean ± standard error (SEM), and the data with non-normal distribution were presented as median(25% percentile, 75% percentile). Statistical differences among groups were analyzed using a one-way ANOVA followed by the LSD post hoc test. Before one-way ANOVA, the normality of data was testified by Kolmogorov–Smirnov tests. If the data distribution is not normal, we used the non-parametric tests. A p value < 0.05 was considered significant. All statistical analyses were conducted using GraphPad Prism for Windows 8.0 (Graphpad Software Inc., CA, USA), SPSS Statistics 26.0 (SPSS Inc., Ill., USA), and Image J (version 1.53 K, National Institutes of Health, USA).

### Ethics approval

The experimental protocols were performed in accordance with the National Institutes of Health Guide for the Care and Use of Laboratory Animals, and were approved by Animal Ethics Committee of Guangxi Medical University (Approval Number: 2023-E369-01).

## Results

### Semaglutide alleviated left ventricular hypertrophy induced by pressure overload

**Figure 1:**
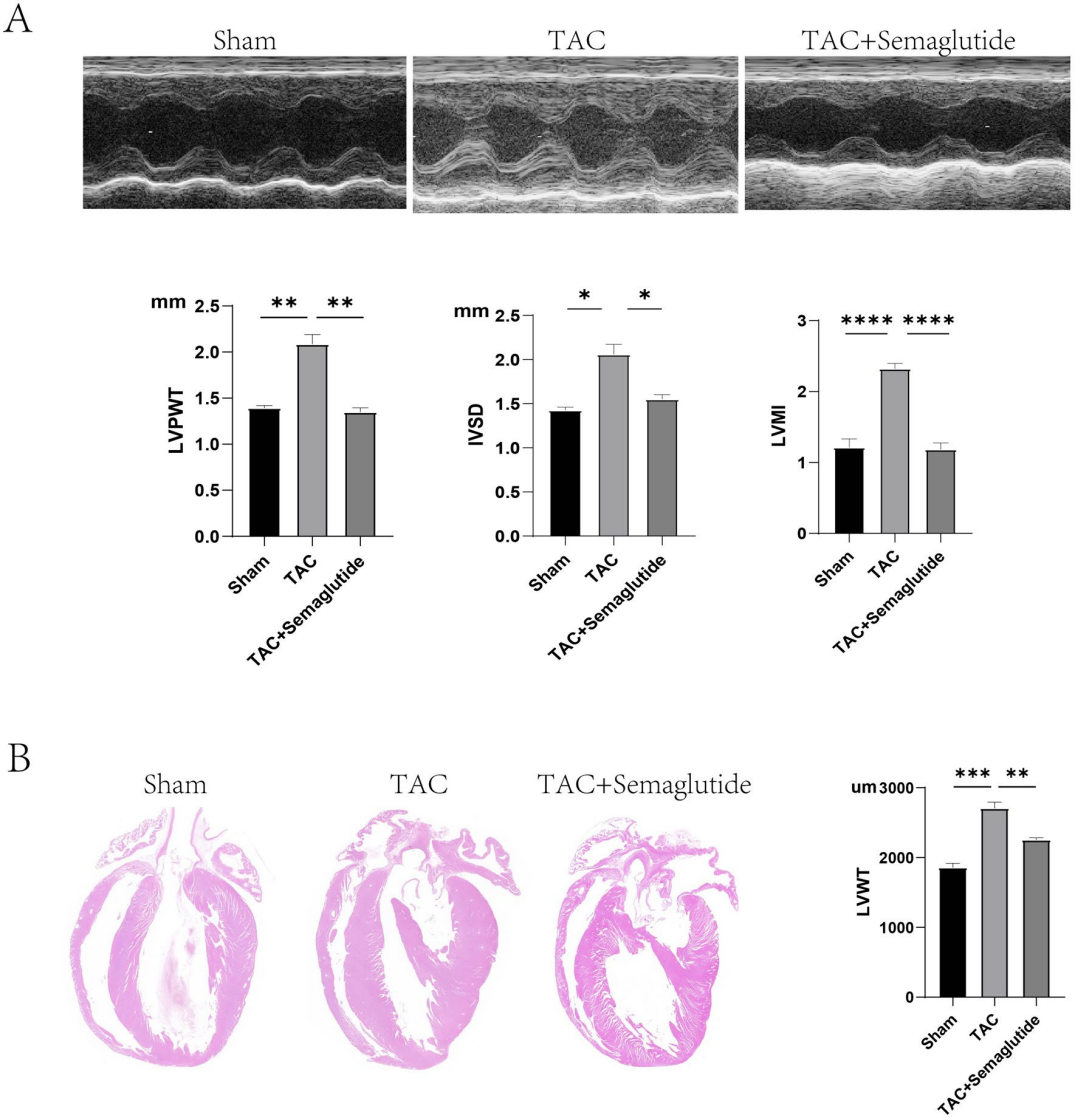
Semaglutide mitigated pressure overload-induced left ventricular hypertrophy. (**A**) Representative images of LVPWT and IVST measured by M-mode doppler echocardiography, and LVPWT, IVST and LVMI of rats in each group(n = 6 in each group) (**B**) Representative longitudinal sections of the whole hearts by HE staining, and LVWT of rats in each group(n = 3 in each group). *LVPWT* left ventricular posterior wall thickness, *IVST* inter-ventricular septum thickness, *LVMI* left ventricular mass index, *LVWT* left ventricular wall thickness, *TAC* transverse aortic constriction. **P < 0.01, ***P < 0.001, and ****P < 0.0001.

We evaluated left ventricular hypertrophy using LVPWT, IVST, and LVMI by echocardiography, and the histomorphology in the longitudinal section of the whole heart by HE staining. The results showed that LVPWT, IVST, and LVMI of rats in TAC group was remarkably enhanced, compared to those of rats in Sham group. However, LVPWT, IVST, and LVMI of rats in TAC + semaglutide group was significantly decreased, compared to those of rats in TAC group (Fig. 1A). Consistently, we found that the left ventricular wall thickness (LVWT) of rats in TAC group was elevated, compared to those of rats in Sham group, but the LVWT of rats in TAC + semaglutide group was lessen by HE staining, compared to those of rats in TAC group (Fig. 1B). However, there were no significant differences in FS and LVEF among the three groups. All the parameters of LV measurement were showed in Supplementary Table 1. These results indicated that semaglutide could ameliorate pressure overload-induced cardiac hypertrophy.

### Semaglutide mitigated pressure overload-induced left ventricular hypertrophy by improving mitophagy dysfunction

**Figure 2:**
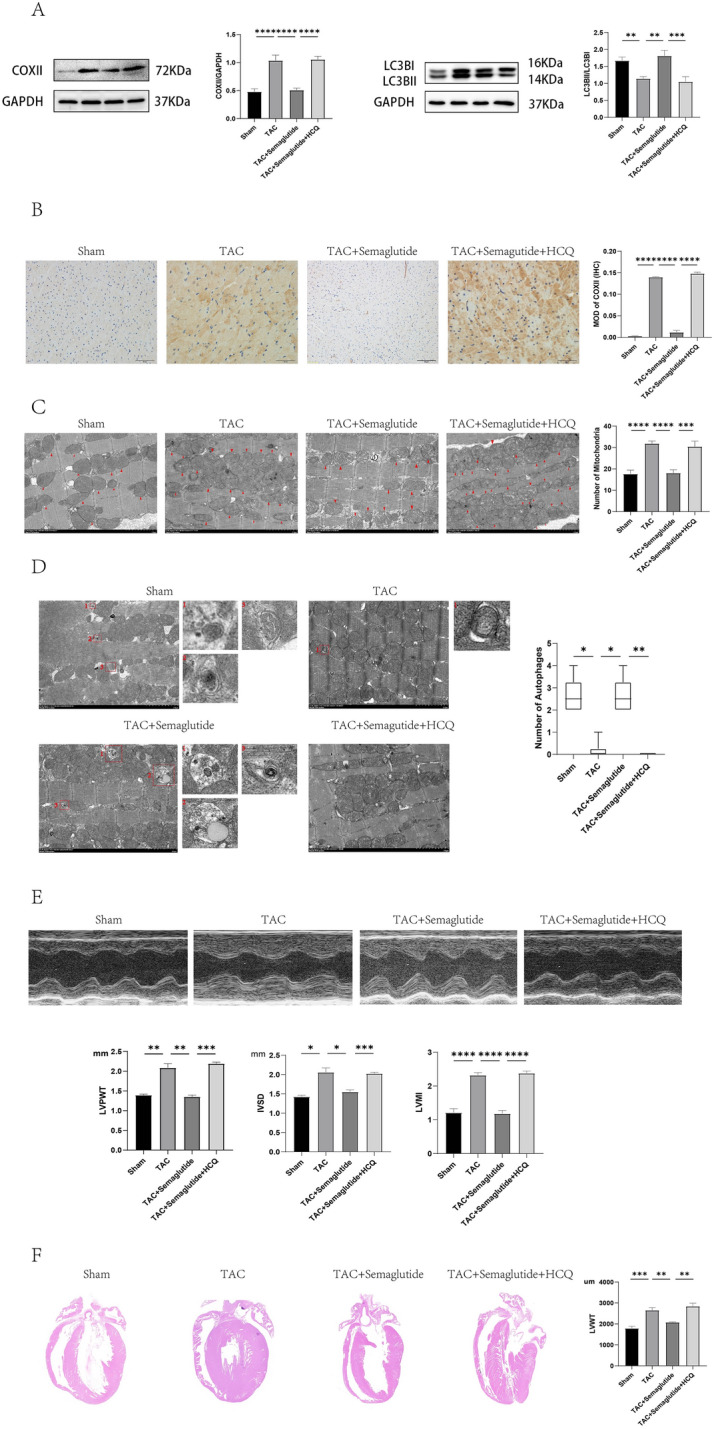
Semaglutide mitigated pressure overload-induced left ventricular hypertrophy by improving mitophagy dysfunction. (**A**) Representative images COX II, LC3I, and LC3II measured by Western-blot, and relative expression of COX II and LC3II/LC3I ratio of rat’s heart in each group (n = 6 in each group). (**B**) Representative immunohistochemical images of COX II (500 ×), and the expression of COX II of rat’s heart in each group (n = 6 in each group). (**C**) Representative images of TEM showing the quantity of mitochondria, and the relative quantity of mitochondria of rat’s heart in each group (7000 ×) (n = 6 in each group). (**D**) Representative images of TEM showing the quantity of autophagosome, and the relative quantity of autophagosome of rat’s heart in each group (7000 ×) (n = 6 in each group). The red arrows indicate typical mitochondria, the autophagosomes were included in a red box, which is amplified in the right pictures. (**E**) Representative images of LVPWT and IVST measured by M-mode doppler echocardiography, and LVPWT, IVST and LVMI of rats in each group(n = 6 in each group). (**F**) Representative longitudinal sections of the whole hearts by HE staining, and LVWT of rats in each group (500 ×) (n = 3 in each group). *LVPWT* left ventricular posterior wall thickness, *IVST* inter-ventricular septum thickness, *LVMI* left ventricular mass index, *LVWT* left ventricular wall thickness, *TAC* transverse aortic constriction, *HCQ* hydroxychloroquine, an inhibitor of mitophagy. *P < 0.05, **P < 0.01, ***P < 0.001, and ****P < 0.0001.

To explore whether semaglutide mitigated pressure overload-induced LVH by improving mitophagy dysfunction, we firstly detected the mitophagy in Sham, TAC, and TAC + semaglutide group, respectively. To reflect the status of mitophagy, we measured COX II and LC3II/LC3I ratio by western-blot, COX II by immuno-histochemistry, quantity of mitochondria and autophagosomes by TEM. The results suggested that the expression of COX II was increased, but the ratio of LC3II/LC3I was decreased in TAC group by western-blot, compared to those in Sham group. Conversely, the expression of COX II was decreased, but the ratio of LC3II/LC3I was elevated in TAC + semaglutide group by western-blot, compared to those in TAC group (Fig. 2A). Similarly, COX II showed a same trend by immuno-histochemistry (Fig. 2B). In addition, the quantity of mitochondria was increased in TAC group, compared to those in Sham group, but the quantity of mitochondria was decreased in TAC + semaglutide group, compared to those in TAC group (Fig. 2C). However, the change on the quantity of autophagosome was converse (Fig. 2D). All above results demonstrated that mitophagy dysfunction may be a cause of cardiac hypertrophy induced by pressure overload, and semaglutide may mitigate pressure overload-induced left ventricular hypertrophy by improving mitophagy.

To further testify our hypothesis, we treated the TAC rats with semaglutide and inhibitor of mitophagy (HCQ). The results showed that LVPWT, IVST, and LVMI of rats in TAC + semaglutide + HCQ group were significantly enhanced by echocardiography, compared to those of rats in TAC + semaglutide group (Fig. 2E), and LVWT of rats in TAC + semaglutide + HCQ group was elevated by HE staining, compared to those of rats in TAC + semaglutide group (Fig. 2F). However, there were no obvious difference on FS and LVEF among four groups. All the parameters of LV measurement were showed in Supplementary Table 1.

Moreover, the expression of COX II was enhanced, but ratio of LC3II/LC3I was decreased in TAC + semaglutide + HCQ group by western-blot, compared to those in TAC + semaglutide group (Fig. 2A), and COX II was elevated in TAC + semaglutide + HCQ group by immuno-histochemistry, compared to those in TAC + semaglutide group (Fig. 2B). Furthermore, the quantity of mitochondria was increased in TAC + semaglutide + HCQ group by TEM, compared to those in TAC + semaglutide group (Fig. 2C), but the quantity of autophagosomes was lessen in TAC + semaglutide + HCQ group by TEM, compared to those in TAC + SMGLT group (Fig. 2D). Thus far, we showed that the reversed effect of semaglutide on cardiac hypertrophy induced by pressure overload is abolished by inhibiting mitophagy. These evidences further verified that semaglutide mitigated pressure overload-induced cardiac hypertrophy by improving mitophagy dysfunction.

### Semaglutide ameliorated pressure overload-induced cardiac hypertrophy by improving mitophagy to suppress the activation of NLRP3 inflammasome

We next investigated the reason why improved mitophagy by semaglutide could ameliorate pressure overload-induced cardiac hypertrophy. We measured the markers of cardiac hypertrophy including MYH7 and ANP, NLRP3 inflammatory signaling pathway including NLRP3, Caspase-1, and IL-18 by Western-blot, and we detected the expression MYH7, ANP, NLRP3, and IL-18 by IHC in Sham group, TAC group, TAC + semaglutide group, and TAC + semaglutide + HCQ group, respectively. We measured the inflammatory cells infiltration by HE staining in Sham group, TAC group, TAC + semaglutide group, and TAC + semaglutide + HCQ group, respectively.

**Figure 3:**
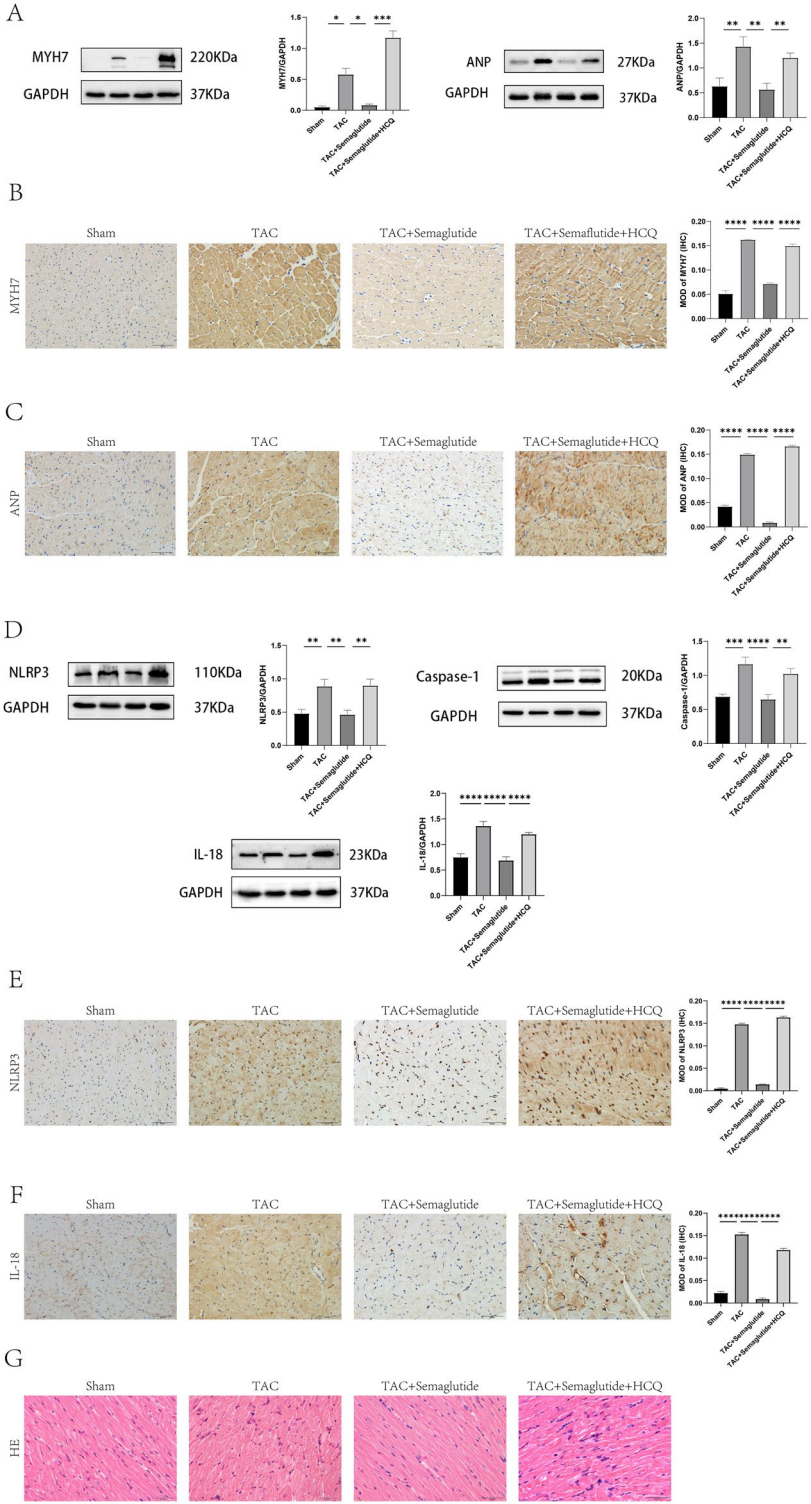
Semaglutide ameliorated pressure overload-induced cardiac hypertrophy by improving mitophagy to suppress the activation of NLRP3 inflammasome. (**A**) Representative images MYH7 and ANP measured by Western-blot, and relative expression of MYH7 and ANP of rat’s heart in each group(n = 6 in each group). (**B**) Representative immunohistochemical images of MYH7 (500 ×), and the expression of MYH7 of rat’s heart in each group(n = 6 in each group). (**C**) Representative immunohistochemical images of ANP (500 ×), and the expression of ANP of rat’s heart in each group(n = 6 in each group). (**D**) Representative images NLRP3, Caspase-1, and IL-18 measured by Western-blot, and relative expression of NLRP3, Caspase-1, and IL-18 of rat’s heart in each group(n = 6 in each group). (**E**) Representative immunohistochemical images of NLRP3 (500 ×), and the expression of NLRP3 of rat’s heart in each group(n = 6 in each group). (**F**) Representative immunohistochemical images of IL-18 (500 ×), and the expression of IL-18 of rat’s heart in each group(n = 6 in each group). (**g**) Representative sections of HE staining to observe inflammatory cell infiltration (500 ×)(n = 6 in each group). *TAC* transverse aortic constriction, *HCQ* hydroxychloroquine, an inhibitor of mitophagy. **P < 0.01, ***P < 0.001, and ****P < 0.0001.

The results revealed that the expression of MYH7 and ANP were significantly elevated in TAC group, compared to the Sham group, but their expression in the TAC + semaglutide group were markedly reduced, compare to the TAC group, by Western blot and IHC respectively. However, the weaken effect of semaglutide on expression of MYH7 and ANP was eliminated by HCQ (Fig. 3A–C). Meanwhile, the expression of NLRP3, Caspase-1, and IL-18 were obviously enhanced in TAC group, compared to the Sham group, but their expression in TAC + semaglutide group were remarkably lessened, compared to the TAC group, by Western-blot and IHC respectively. However, the inhibitory effect of semaglutide on NLRP3 inflammasome activation was counteracted by HCQ (Fig. 3D–F). Furthermore, the results uncovered that there were more inflammatory cells infiltration in TAC group and TAC + semaglutide + HCQ than those in Sham group and TAC + semaglutide group by HE staining(Fig. 3G).

**Figure 4:**
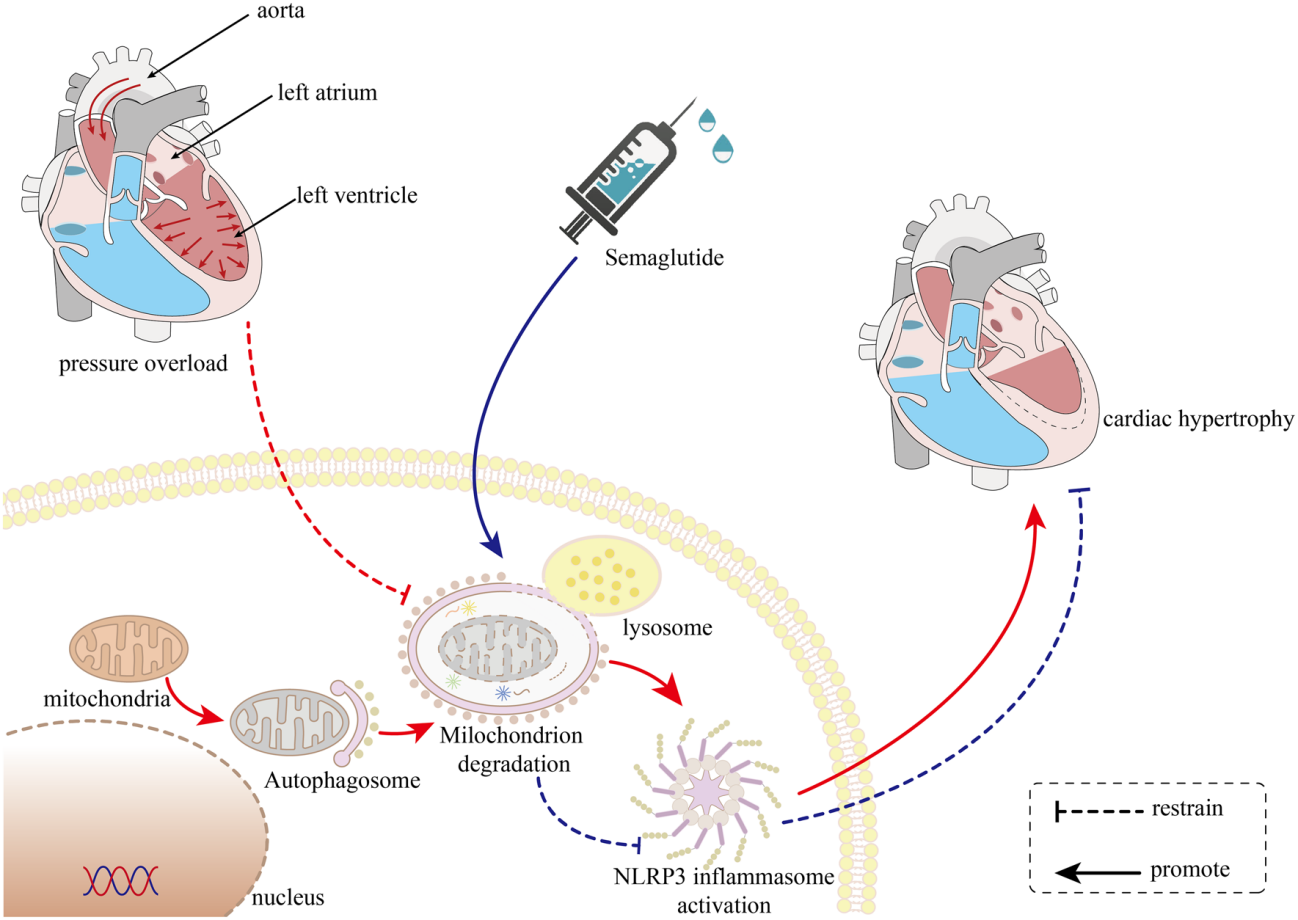
The potential mechanism of semaglutide improving cardiac hypertrophy induced by pressure overload.

Our evidences suggested that impaired mitophagy may be a cause of cardiac hypertrophy induced by pressure overload, and semaglutide ameliorated the cardiac hypertrophy by improving mitophagy to suppress the activation of NLRP3 inflammasome (Fig. 4).

## Discussion

In present study, we find that dysfunction of mitophagy may play an important role in pressure overload-induced cardiac hypertrophy, and semaglutide ameliorates this type of cardiac hypertrophy by improving mitophagy to suppress the activation of NLRP3 inflammasome.

At present, there are so many people with LVH because massively absolute number of hypertensive people. LVH is a crucial structure structural changes to develop into HF. In clinical, although the blood pressure of some hypertensive patients is controlled, LVH is still present. Therefore, we wanted to look for some novel potential strategies to improve LVH. In recent studies, GLP1R agonists have shown a significantly cardiac protective effects in the patients with type 2 diabetes^9^. In addition, some studies have shown that GLP1R agonists can improve mortality, increase left ventricular ejection fraction, and attenuate the level of brain natriuretic peptide in the patients with type 2 diabetes and HF. Therefore, we focused on the effect of semaglutide, a long-acting GLP1R agonist, on cardiac hypertrophy induced by pressure overload in this study.

We mimicked condition of pressure overload in rats by TAC and observed the effect of semaglutide on LVH in TAC rats. The results intuitively showed that LVH induced by TAC in rats was attenuated by the administration of semaglutide. Hypertrophic cardiomyocytes decrease the ventricular diastolic function, and enhanced ventricular filling pressure in diastolic phase, which is a main cause of heart failure with preserved ejection fraction (HFpEF). A recent study indicated that semaglutide led to larger reductions in symptoms and physical limitations, greater improvements in exercise function in patients with HFpEF and obesity^32^. Regrettably, whether the ventricular diastolic function was improved, and the intracardiac pressure was decreased were not be evaluated in this study. In accord with this study, a better condition was observed in rats with semaglutide in our study. Furthermore, our evidences revealed that better condition in rats with semaglutide is at least partially attributed to improved LVH. In Withaar et al.’s study, aged mice were administrated with angiotensin II and a high-fat diet to mimic HFpEF model. They revealed that liraglutide attenuated cardiac hypertrophy in mice with HFpEF^33^. Their results are similar with ours, but they used more hits including senescence, hyperlipidemia, and pressure overload to mimic LVH. In another study, liraglutide reduced the LVPWT of rats with abdominal aortic constriction^34^. The differences between our study and their study were that heart dilation and systolic dysfunction were present because of 16 weeks follow-up in their study. Their study suggests that liraglutide may reverse cardiac remolding in relatively late stage. The reason why we chose a 4 weeks follow-up time was that we wanted to observe the effects of semaglutide on cardiac hypertrophy in relatively early stage of HF. In addition, a recent study showed HF was present 45 days after TAC^35^. Our findings, corroborated by the outcomes of two additional studies, furnish evidence supporting the potential of GLP-1R agonists to mitigate LVH in animal models.

Next, we investigated the potential mechanism of improved cardiac hypertrophy by semaglutide. We found obviously cardiac mitophagy dysfunction and LVH in TAC group, compared to that in Sham group, and the cardiac mitophagy dysfunction and LVH induced by pressure overload were significantly improved by semaglutide. However, The improved mitopahgy and LVH were completely abolished by an inhibitor of mitophagy. These results uncovered that pressure overload impaired cardiac mitophagy, and reversed effect of semaglutide on LVH was correlative with increased cardiac mitophagy. Some previous studies have also reported that mitophagy play a cardiac protective effect during pressure overload in heart^16,17^, which is similar to our study.

Subsequently, we explore the reason why improved mitophagy can ameliorate LVH induced by pressure overload. We found that pressure overload induced cardiac mitophagy dysfunction, as well as activation of NLRP3 inflammasome. Meanwhile, the expression of hypertrophic marker, such as ANP and MYH7, were enhanced under pressure overload. Of note, semaglutide markedly improved cardiac mitophagy, suppressed the activation of NLRP3 inflammasome, and down-regulated the expression of ANP and MYH7. However, all the effects of semaglutide were abolished by the inhibitor of mitophgay. These results suggested that the inhibitory effect of semaglutide on NLRP3 inflammasome was associated with improved mitophagy by semaglutide. NLRP3 inflammasome activation is an important mechanism of cardiac hypertrophy^36,37^. As previously reported, mitophagy dysfunction is closely associated with activation of NLRP3 inflammasome^19,38,39^. Therefore, we think that cardiac mitophagy dysfunction induced by pressure overload activates NLRP3 inflammasome to cause cardiac hypertrophy.

In the present study, we found that cardiac mitophagy dysfunction induced by pressure overload can activate NLRP3 inflammasome, and lead to cardiac hypertrophy, and improving mitophagy by semaglutide could suppress the activation of NLRP3 inflammasome to alleviate cardiac hypertrophy. To the best of our knowledge, it is the first study to report that semaglutide ameliorates pressure overload-induced cardiac hypertrophy by improving cardiac mitophagy to suppress the activation of NLRP3 inflammasome. Our study may provide a novel potential option for intervention of cardiac hypertrophy induced by pressure overload.

## Conclusion

Our study reveals that the activation of NLRP3 inflammasome caused by mitophagy dysfunction plays an important role in cardiac hypertrophy induced by pressure overload, and semaglutide ameliorates the cardiac hypertrophy by improving cardiac mitophagy to suppress the activation of NLRP3 inflammasome. Semaglutide may be a novel potential option for intervention of cardiac hypertrophy induced by pressure overload.

## Limitations

However, our current study has several limitations. Primarily, given that our conclusions are derived from a small sample size and observational study utilizing an animal model, these results need to be further verified in the population with pressure overload by large-sample prospective randomized controlled trial. Second, we constructed pressure-overload model by TAC, but this model cannot completely mimic the status of hypertensive patients. Third, there were male rats in our study. We selected only male rats, based on findings from previous research indicating that male mice showed a stronger response to cardiac pressure overload in terms of LVH development^40^. However, whether the reserved effect of semaglutide on LVH induced by pressure overload is similar in female rats is still uncertain, and needs to verify in future study. Finally, we only observe that pressure overload can lead to mitophagy dysfunction, but the mechanism of mitophagy dysfunction is not fully elucidated in current study. We will investigate it in our future study.

### Supplementary Information

Supplementary Information 1.

Supplementary Information 2.

Supplementary Information 3.

Supplementary Information 4.

Supplementary Information 5.

Supplementary Information 6.

Supplementary Information 7.

Supplementary Information 8.

Supplementary Information 9.

Supplementary Information 10.

Supplementary Information 11.

Supplementary Information 12.

Supplementary Information 13.

Supplementary Information 14.

Supplementary Information 15.

Supplementary Information 16.

Supplementary Information 17.

Supplementary Information 18.

Supplementary Information 19.

Supplementary Information 20.

Supplementary Information 21.

Supplementary Information 22.

Supplementary Information 23.

Supplementary Information 24.

Supplementary Information 25.

Supplementary Information 26.

Supplementary Information 27.

Supplementary Information 28.

Supplementary Information 29.

Supplementary Information 30.

Supplementary Information 31.

Supplementary Information 32.

Supplementary Information 33.

Supplementary Information 34.

Supplementary Information 35.

Supplementary Information 36.

Supplementary Information 37.

Supplementary Information 38.

Supplementary Information 39.

Supplementary Table 1.

Supplementary Table 2.

Supplementary Table 3.

Supplementary Table 4.

Supplementary Information 40.

## Data Availability

The datasets of the present study are available from the corresponding author upon request.
