# Supplementary figures and images for: Semaglutide ameliorates pressure overload-induced cardiac hypertrophy by improving cardiac mitophagy to suppress the activation of NLRP3 inflammasome
Source: Sci Rep. 2024 May 23;14:11824. doi: 10.1038/s41598-024-62465-6 (PMC11116553; doi:10.1038/s41598-024-62465-6)

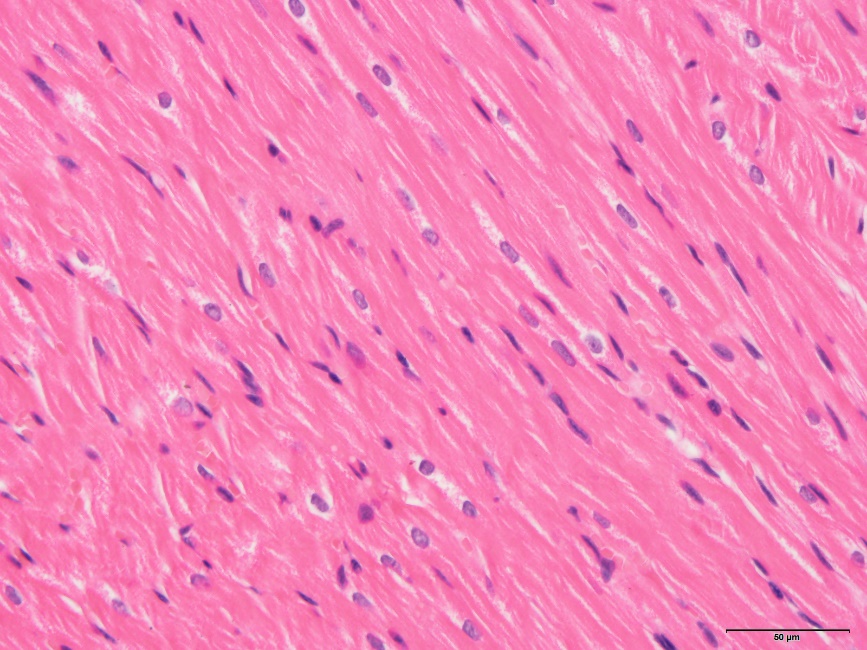

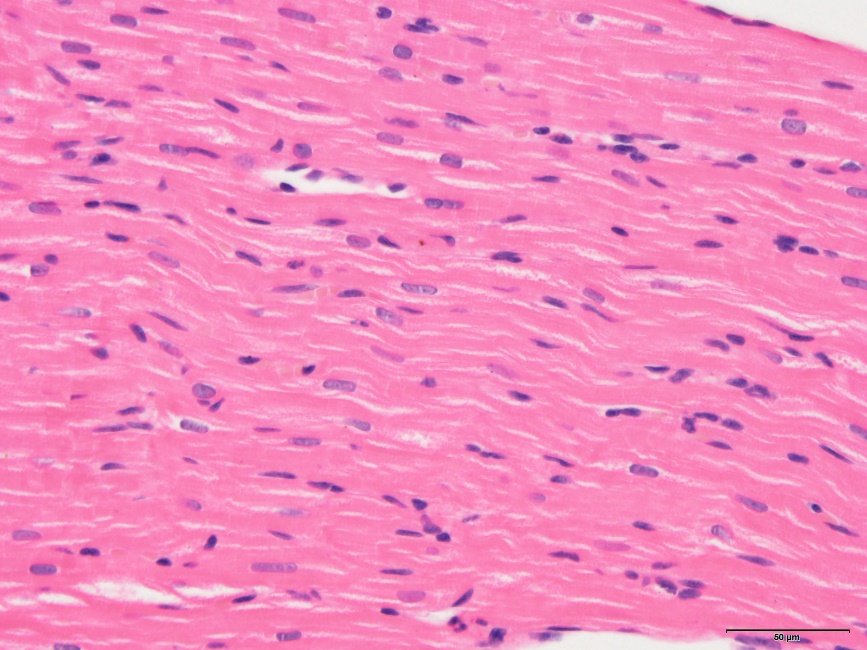

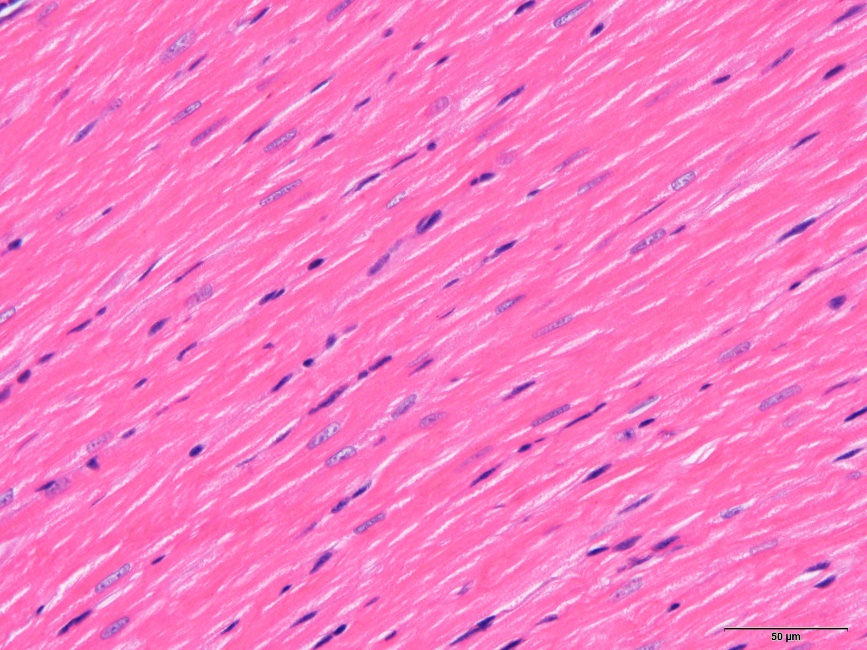

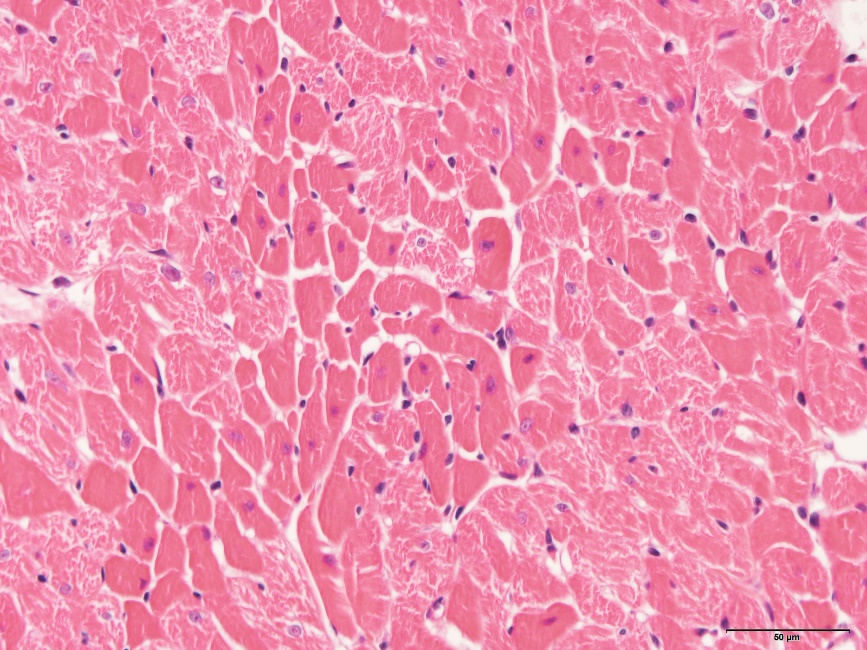

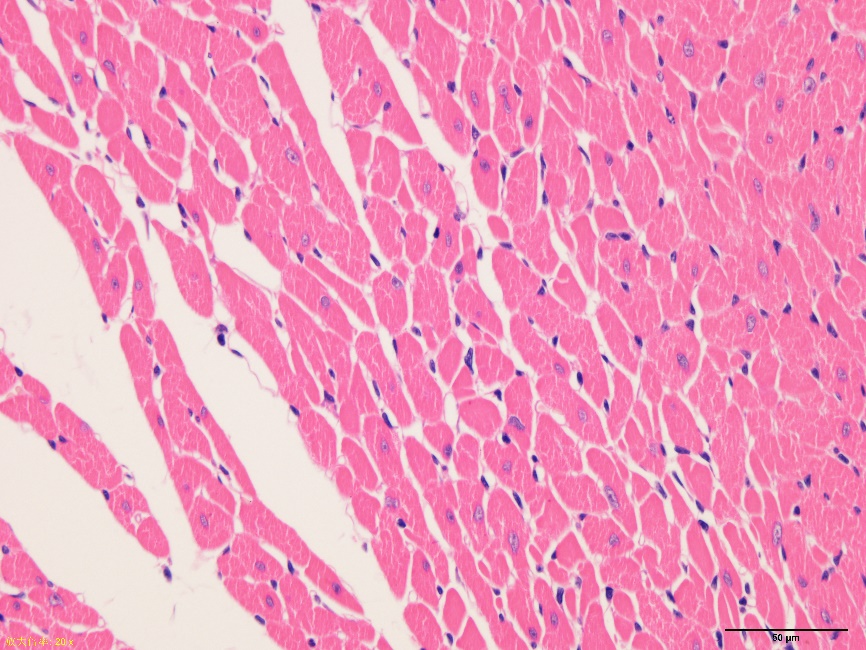

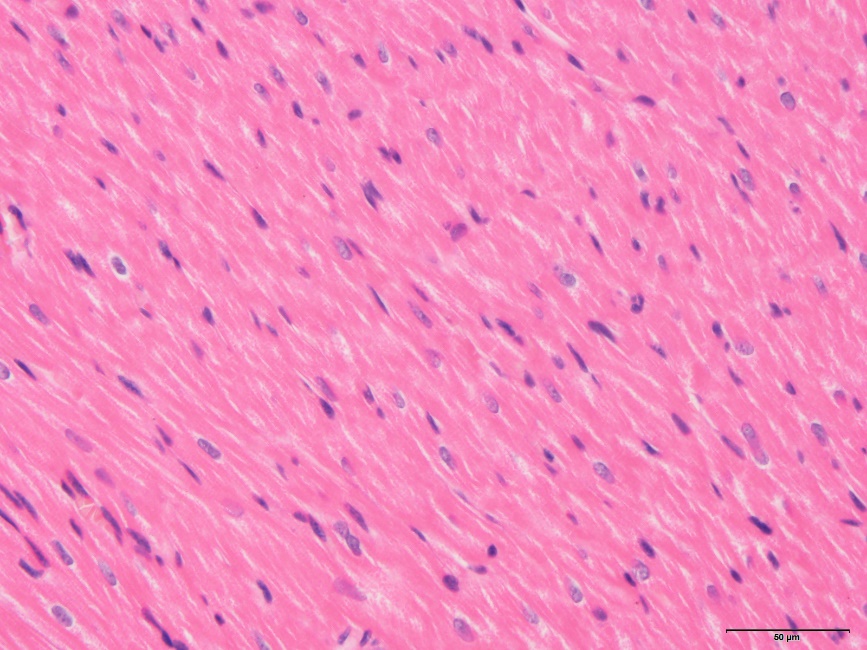


This picture was showed in our manuscript (Fig. 3G).

Sham

Supplement: Supplementary file 5 — Supplementary Information 5. [file 41598_2024_62465_MOESM5_ESM.docx]

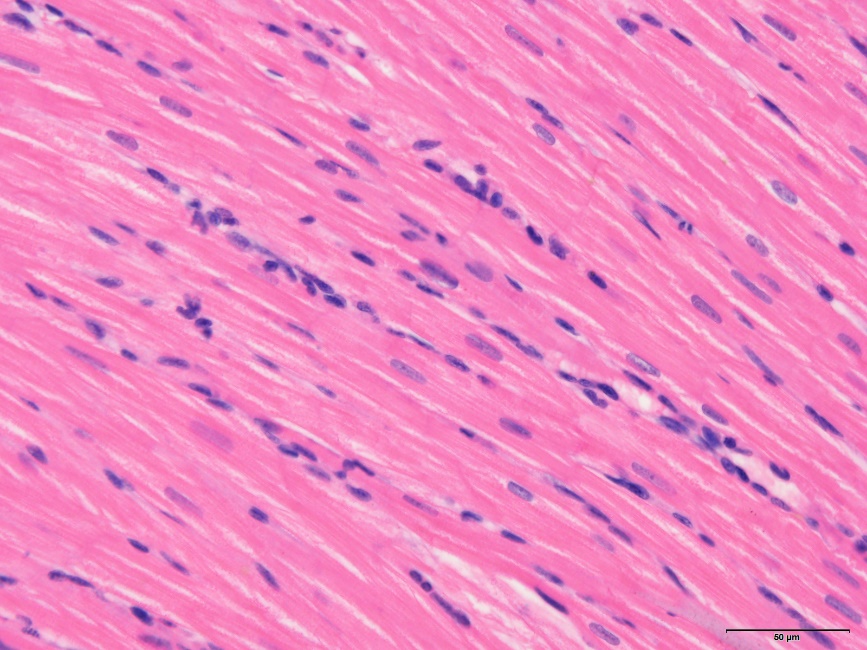

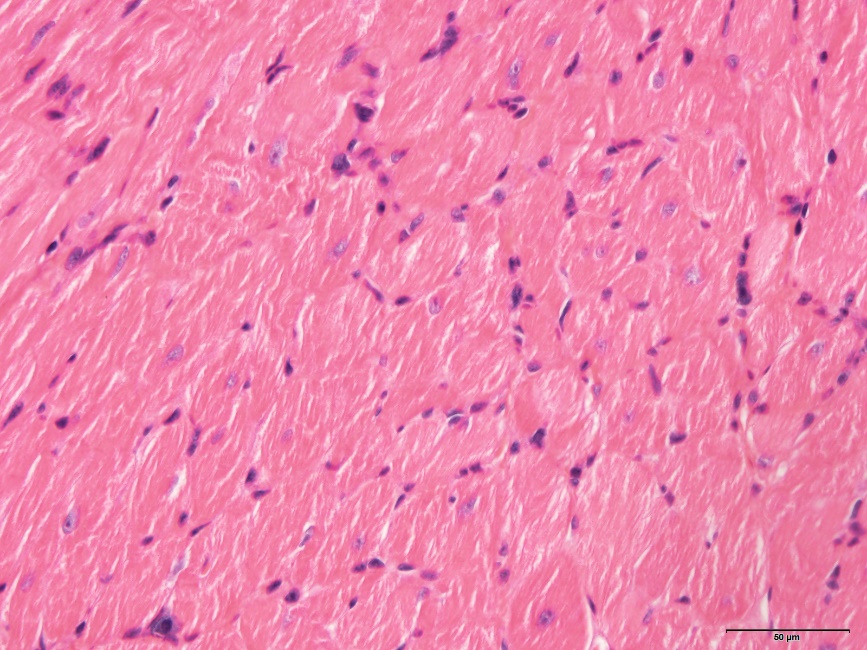

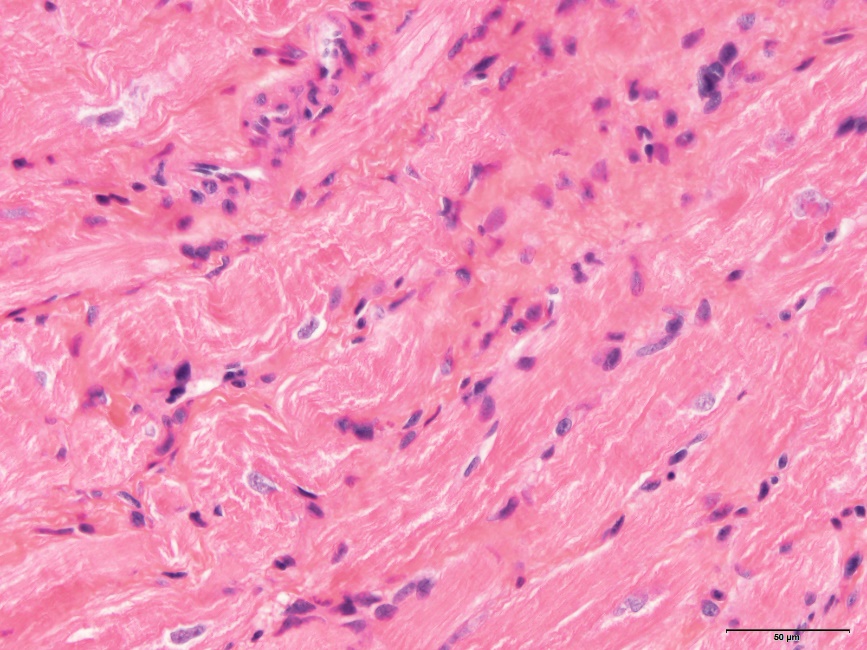

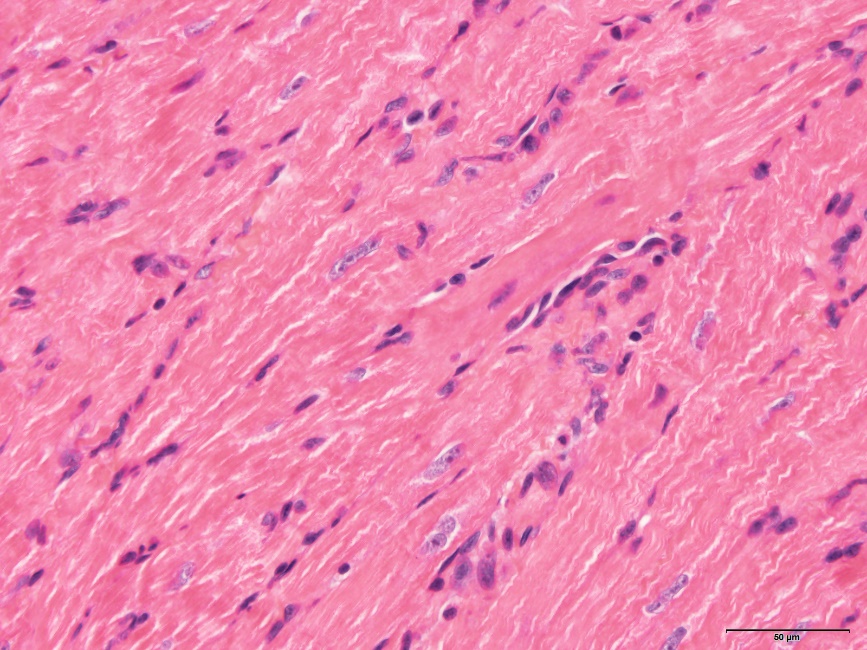

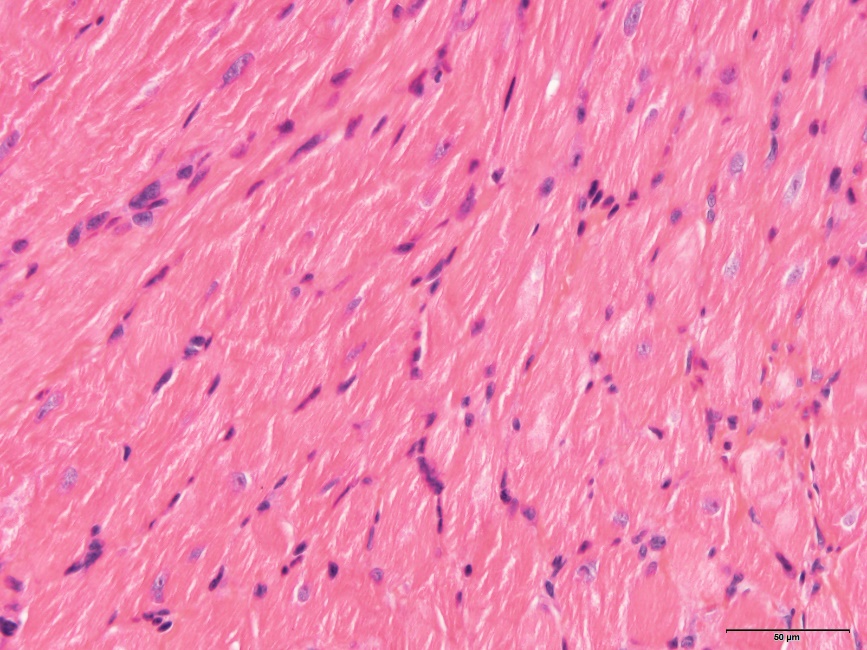

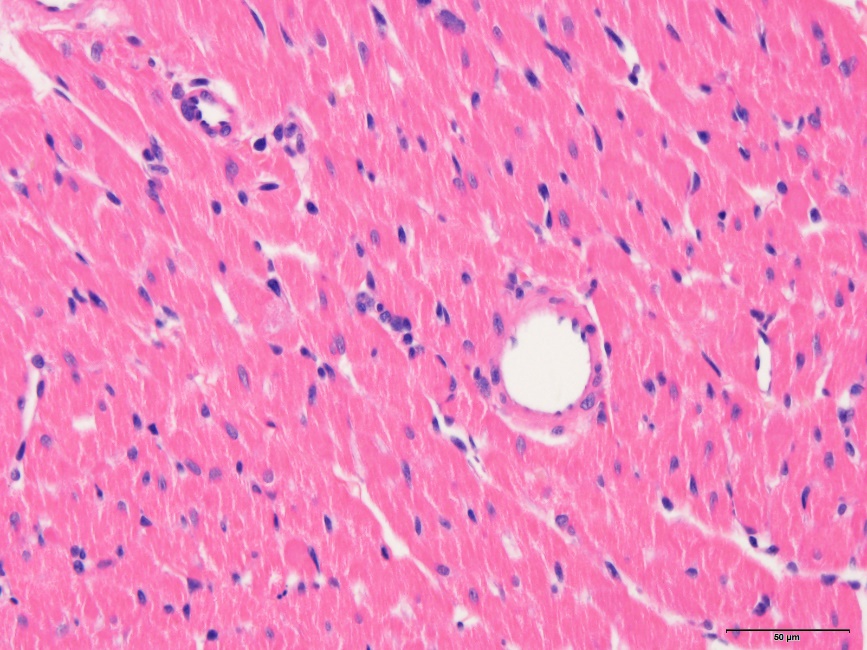


TAC

This picture was showed in our manuscript (Fig. 3G).

Supplement: Supplementary file 6 — Supplementary Information 6. [file 41598_2024_62465_MOESM6_ESM.docx]

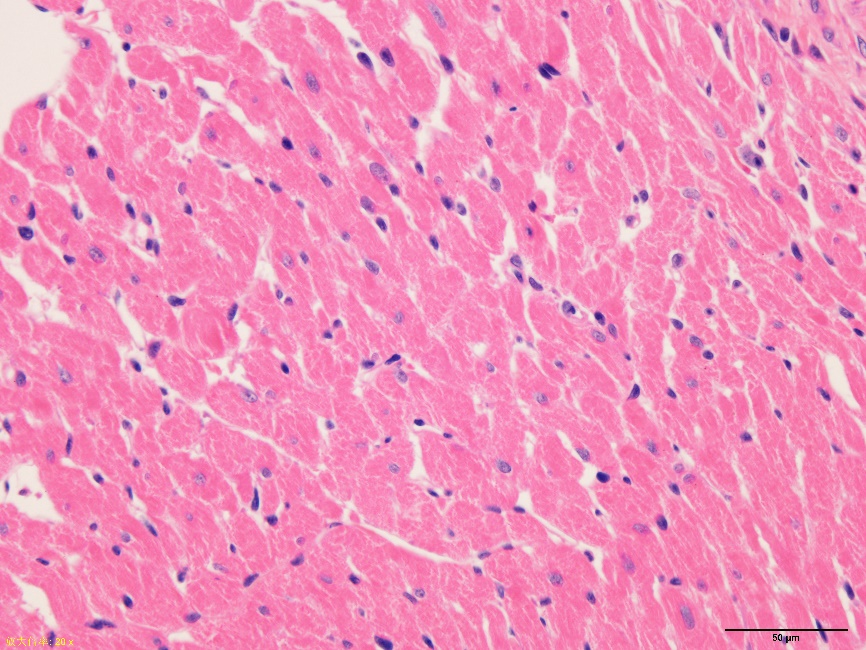

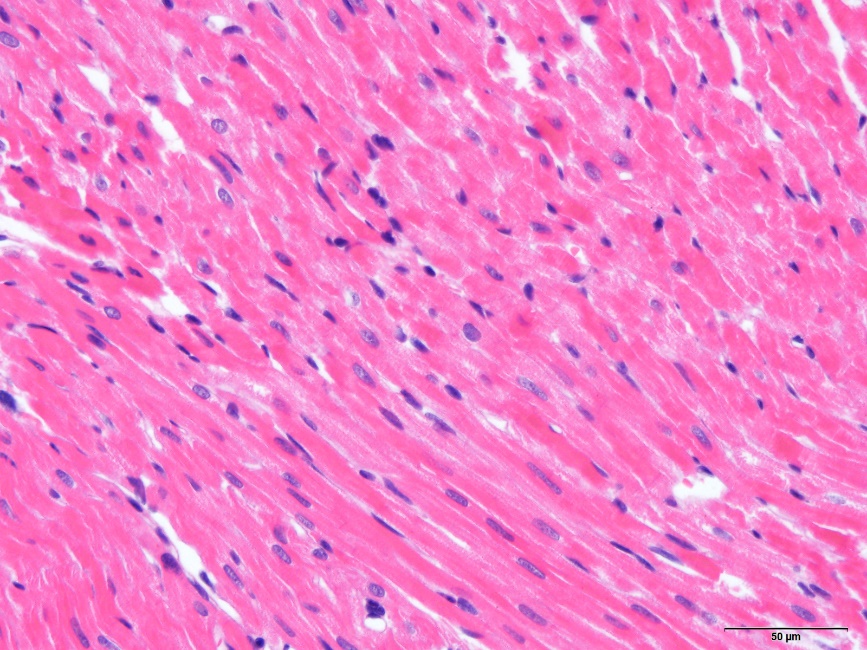

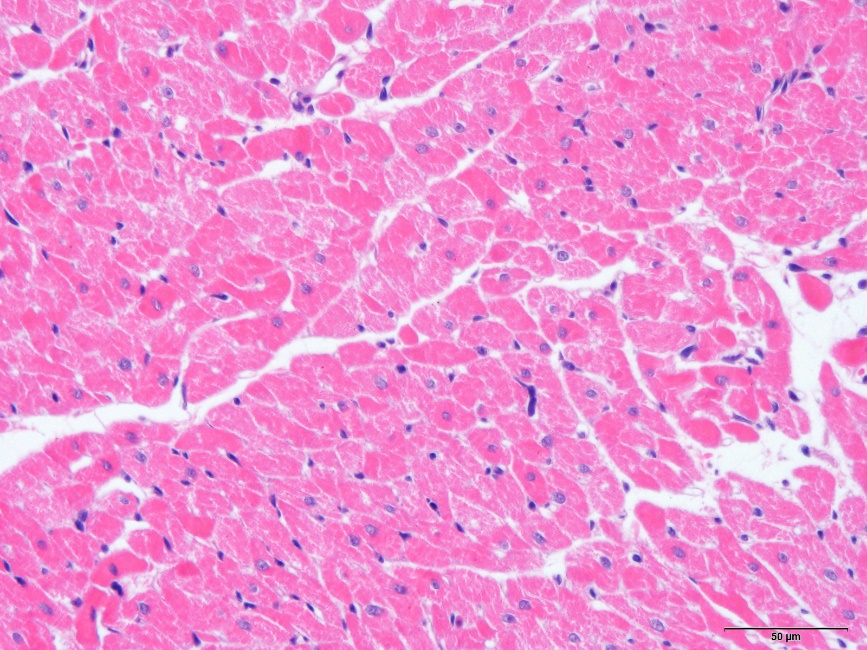

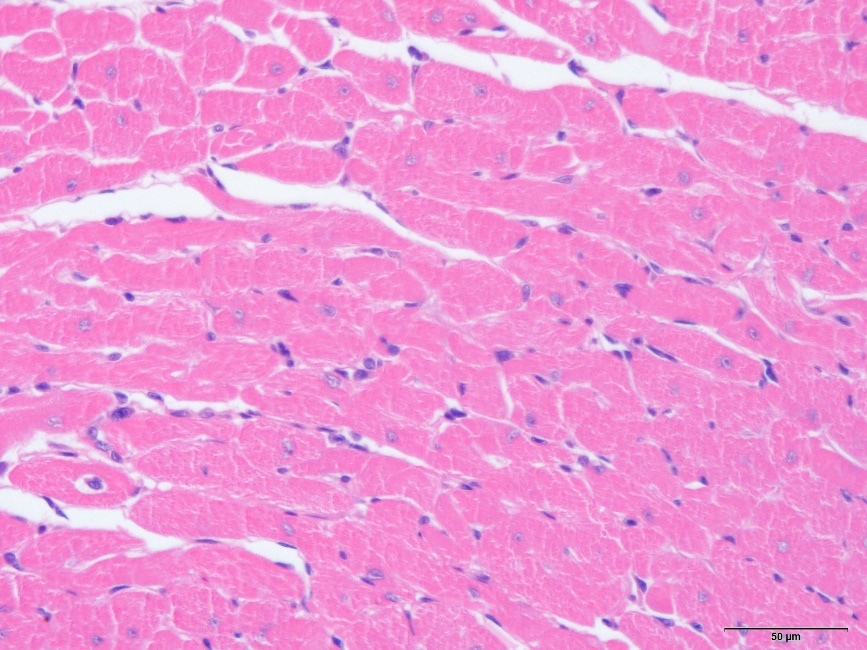

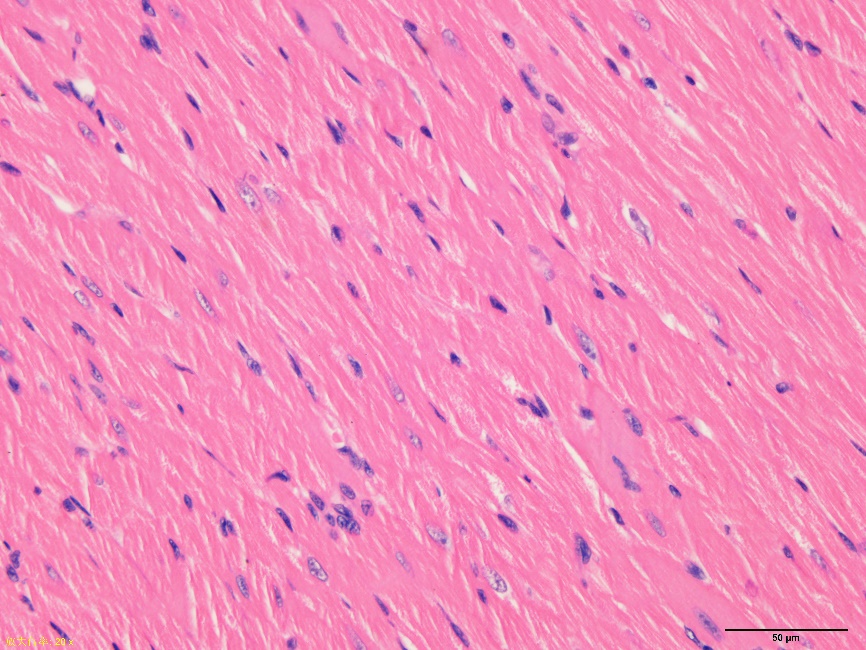

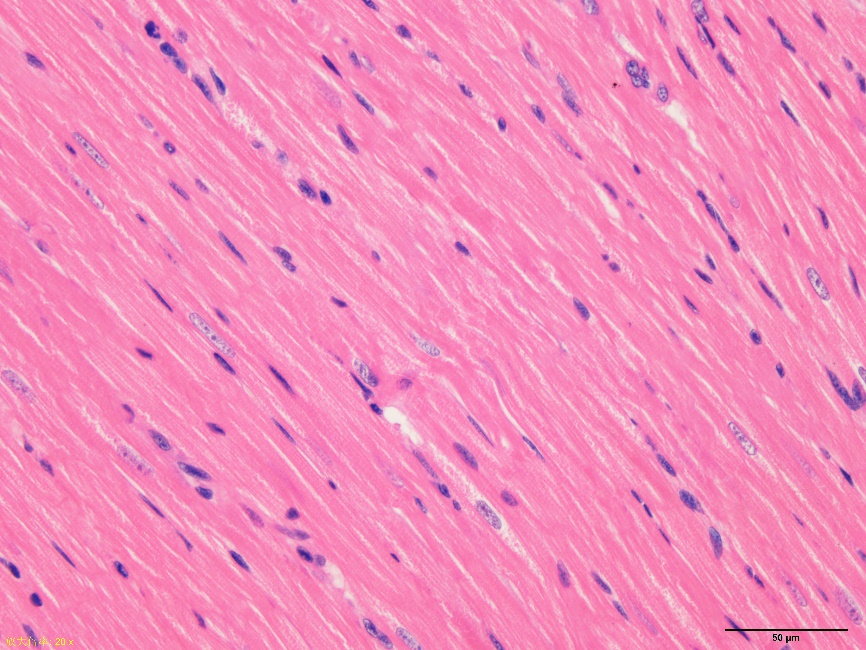


TAC+Semaglutide

This picture was showed in our manuscript (Fig. 3G).

Supplement: Supplementary file 7 — Supplementary Information 7. [file 41598_2024_62465_MOESM7_ESM.docx]

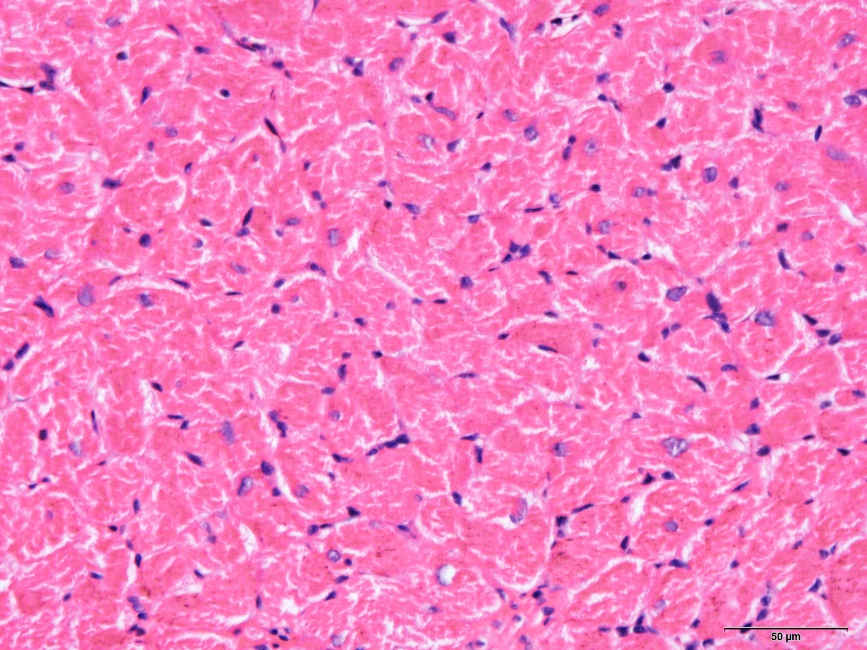

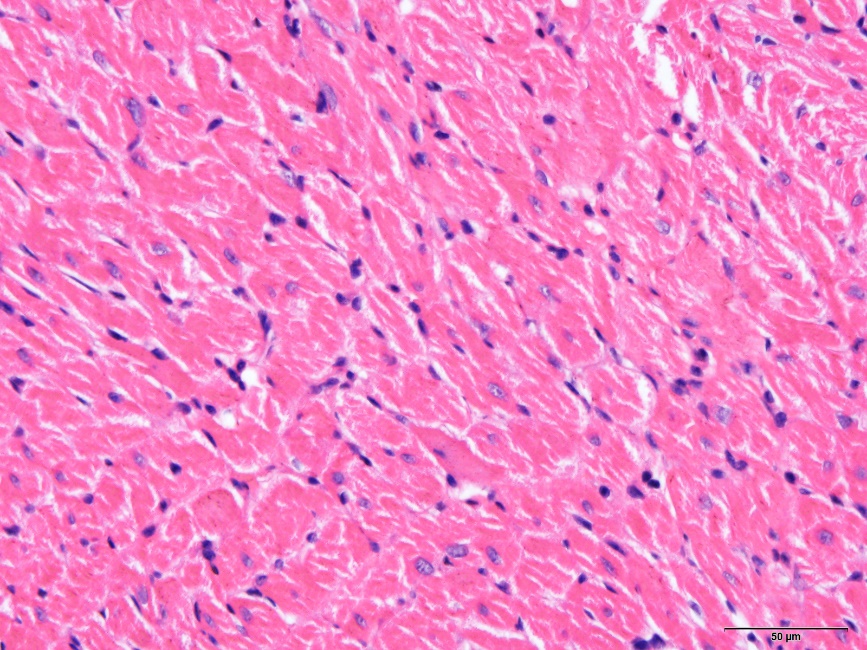

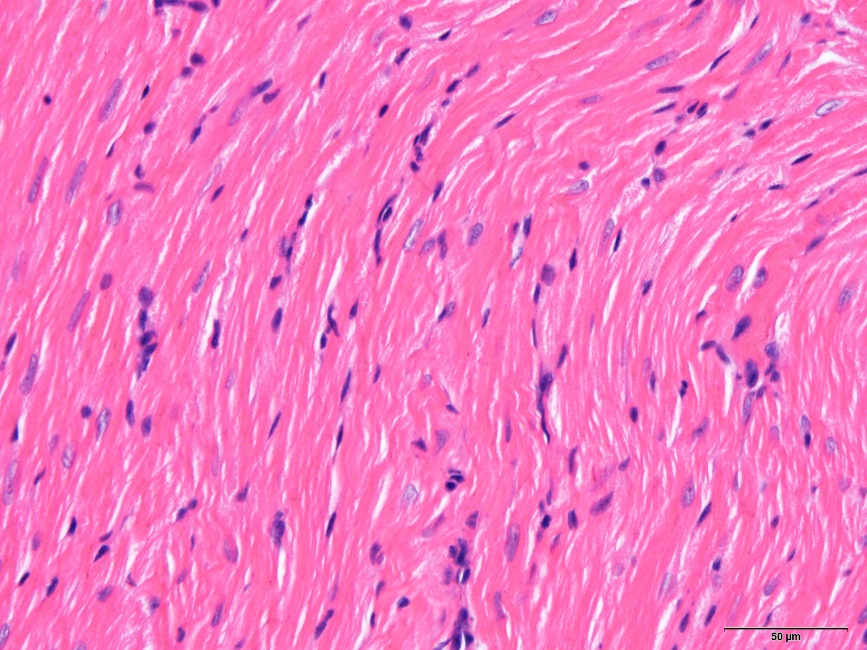

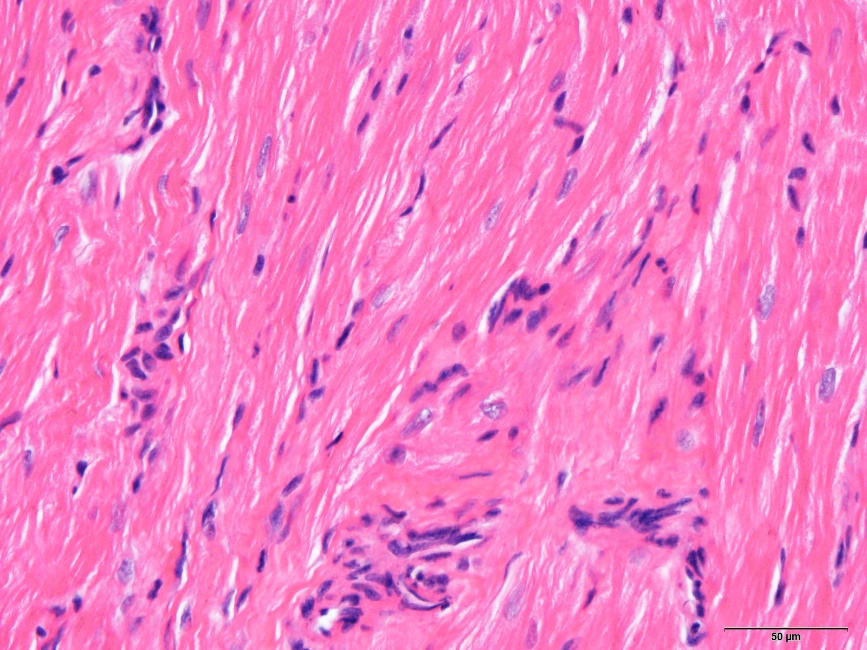

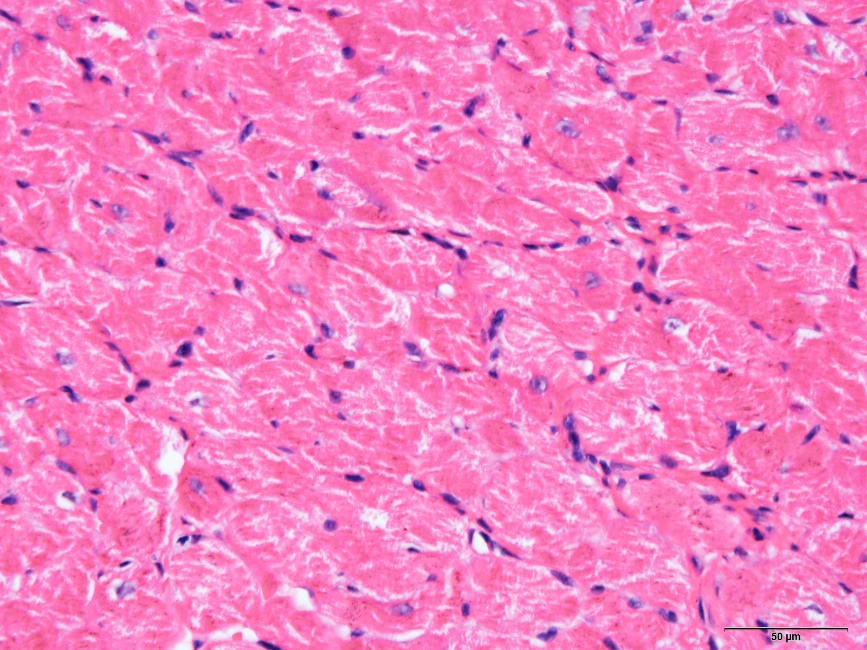

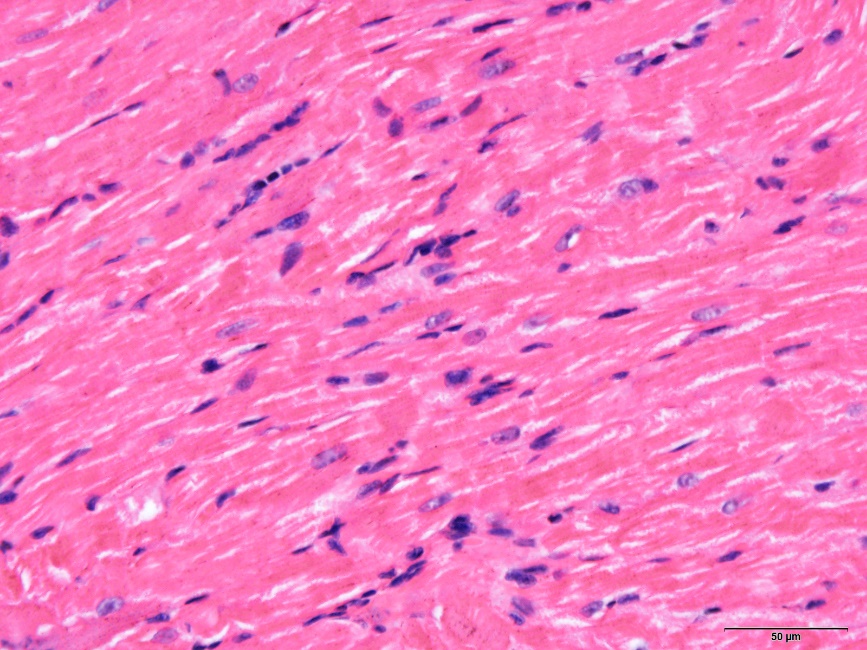


TAC+Semaglutide+HCQ

This picture was showed in our manuscript (Fig. 3G).

Supplement: Supplementary file 8 — Supplementary Information 8. [file 41598_2024_62465_MOESM8_ESM.docx]

Sham-IVSD


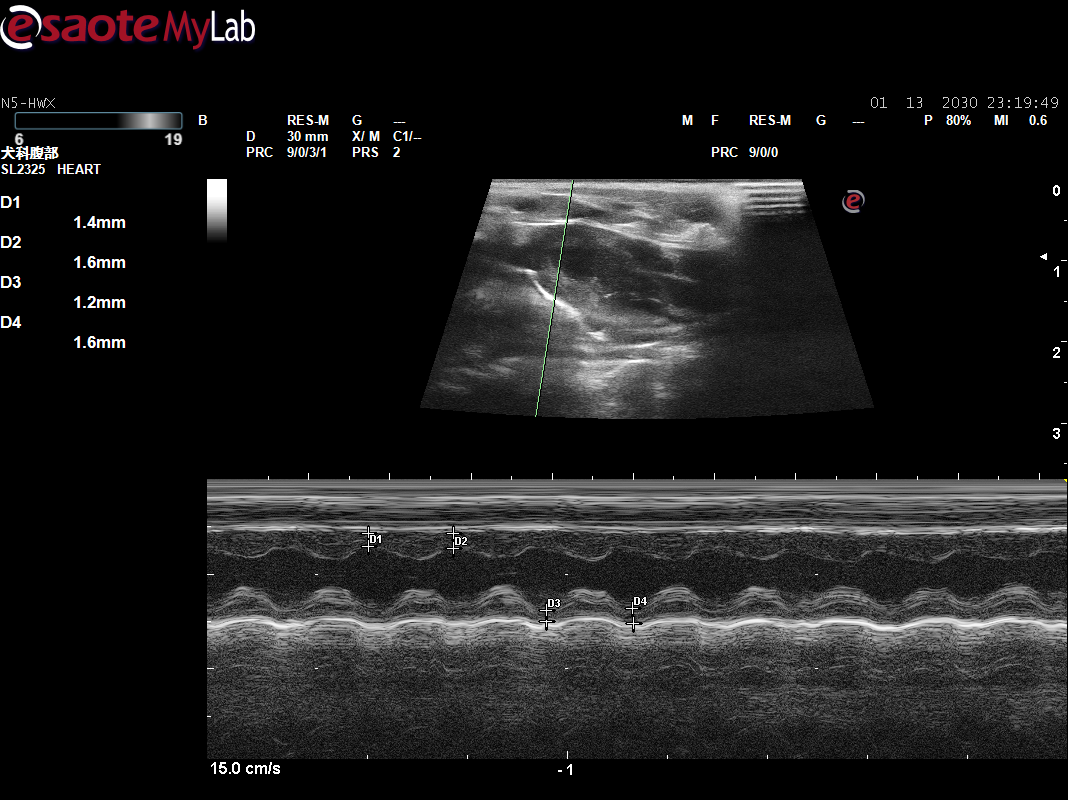

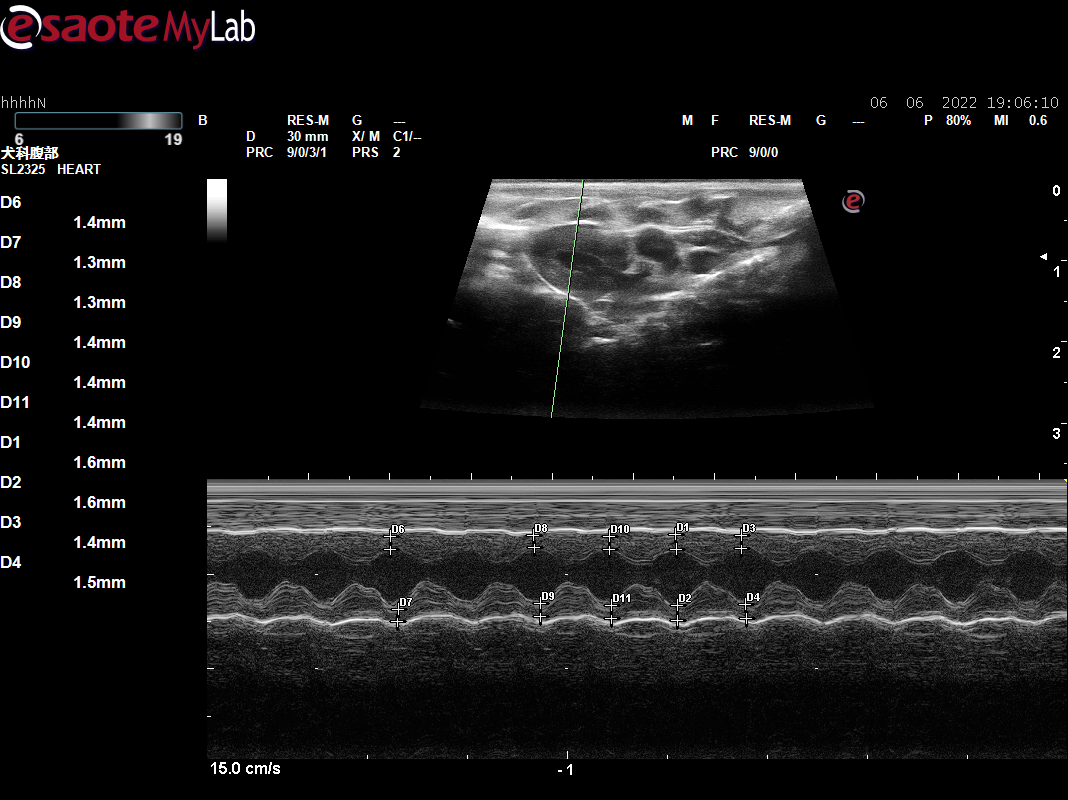

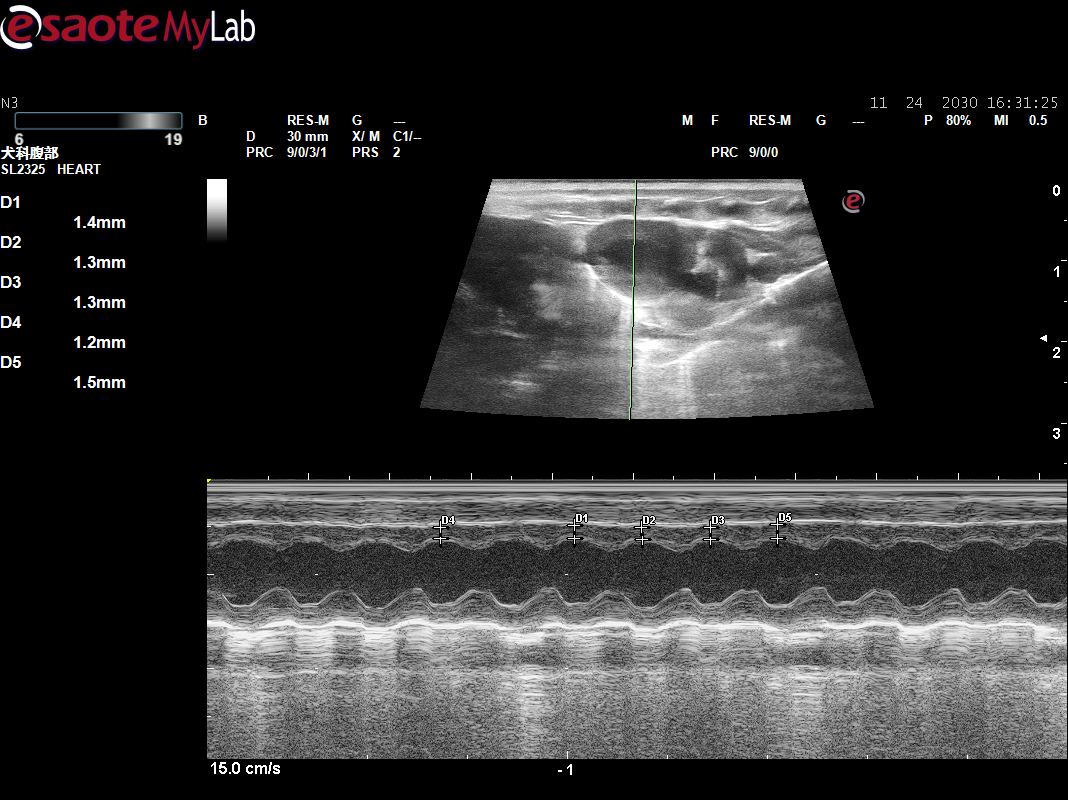

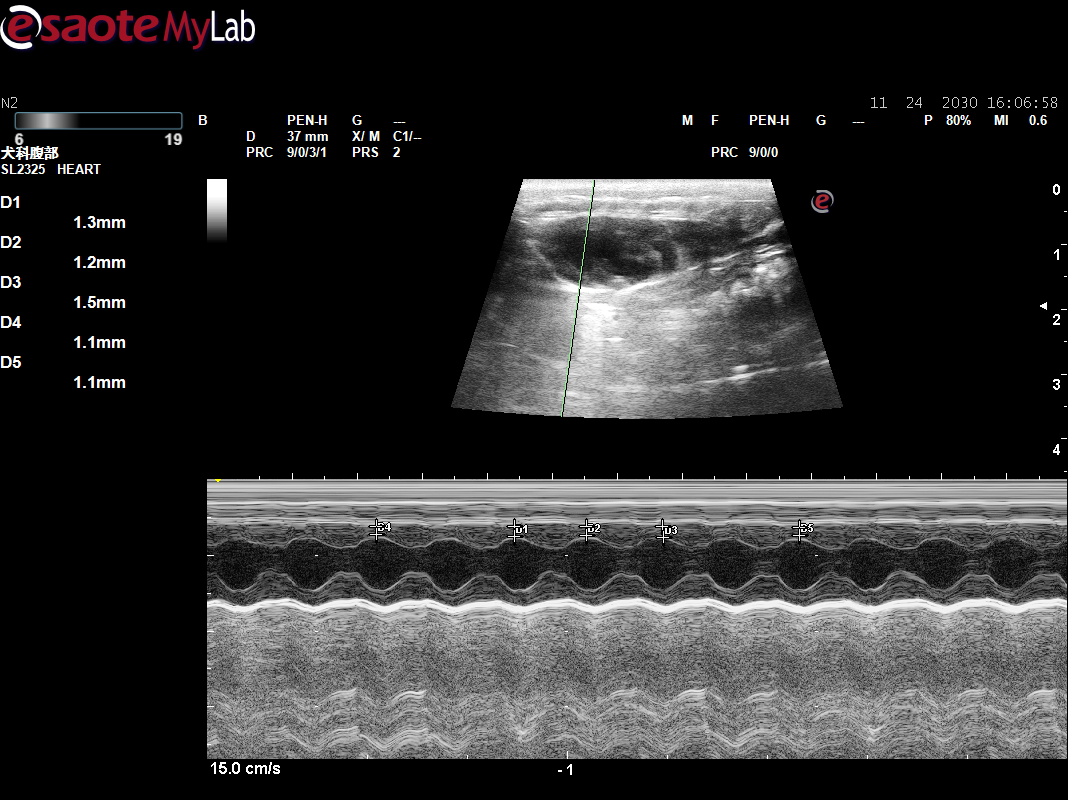


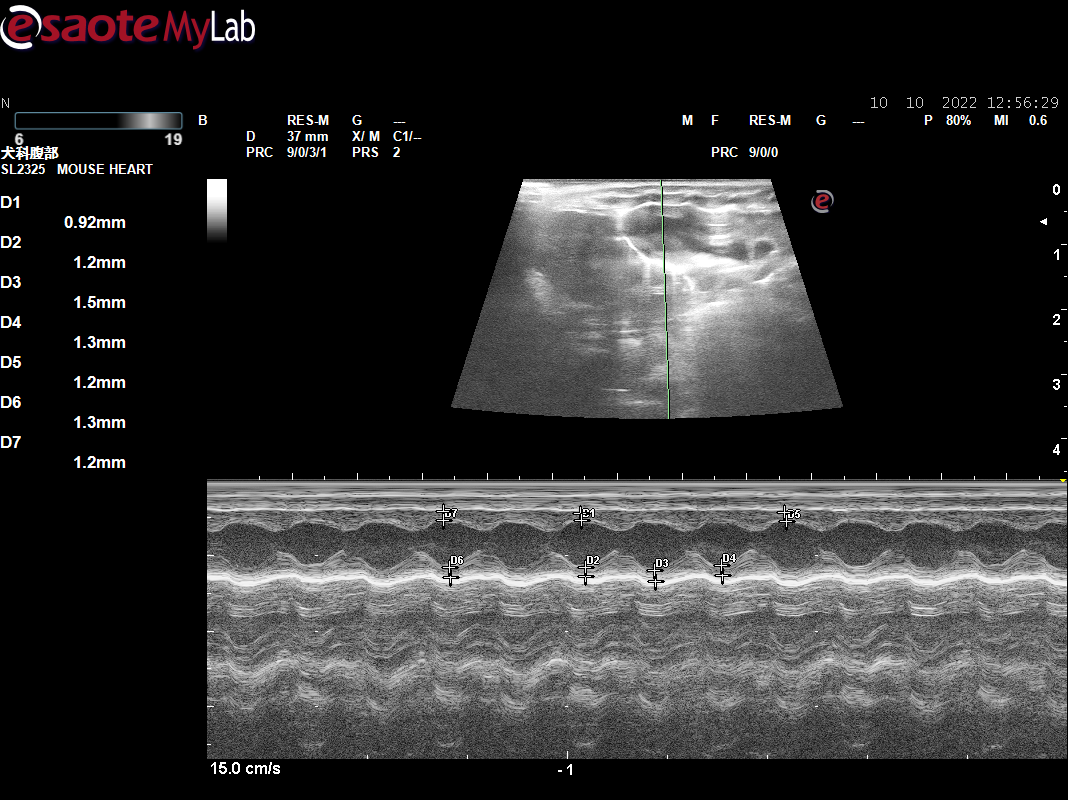

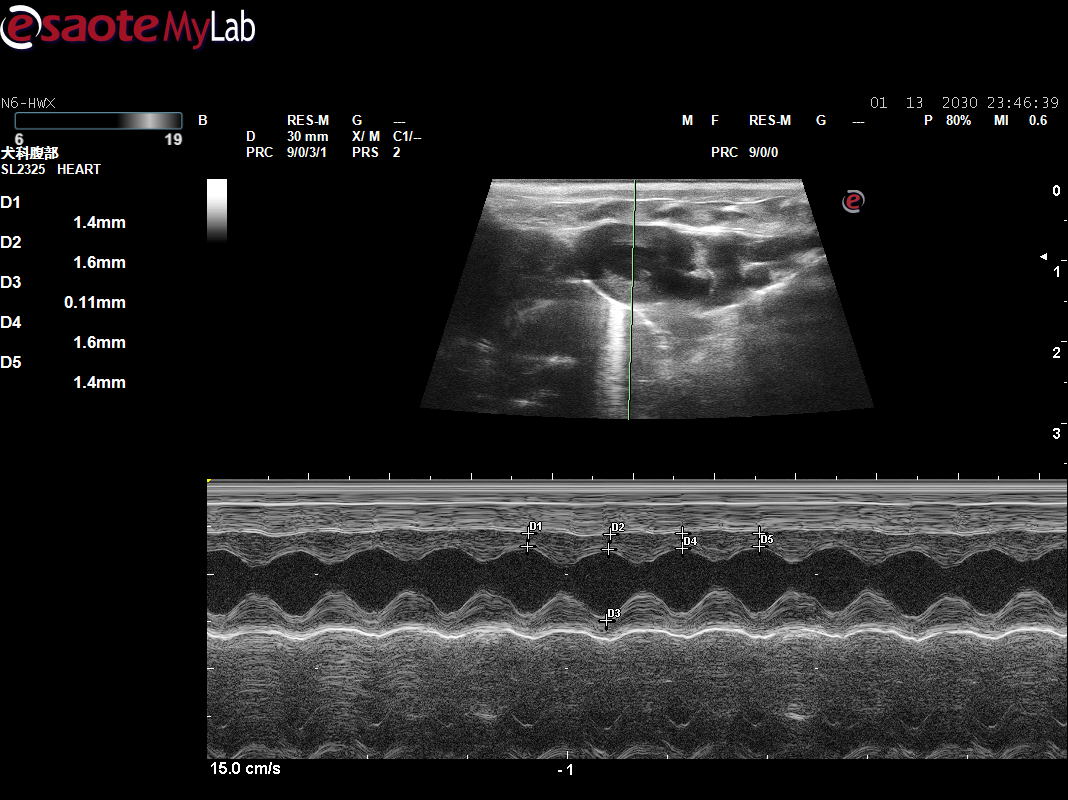


TAC-IVSD


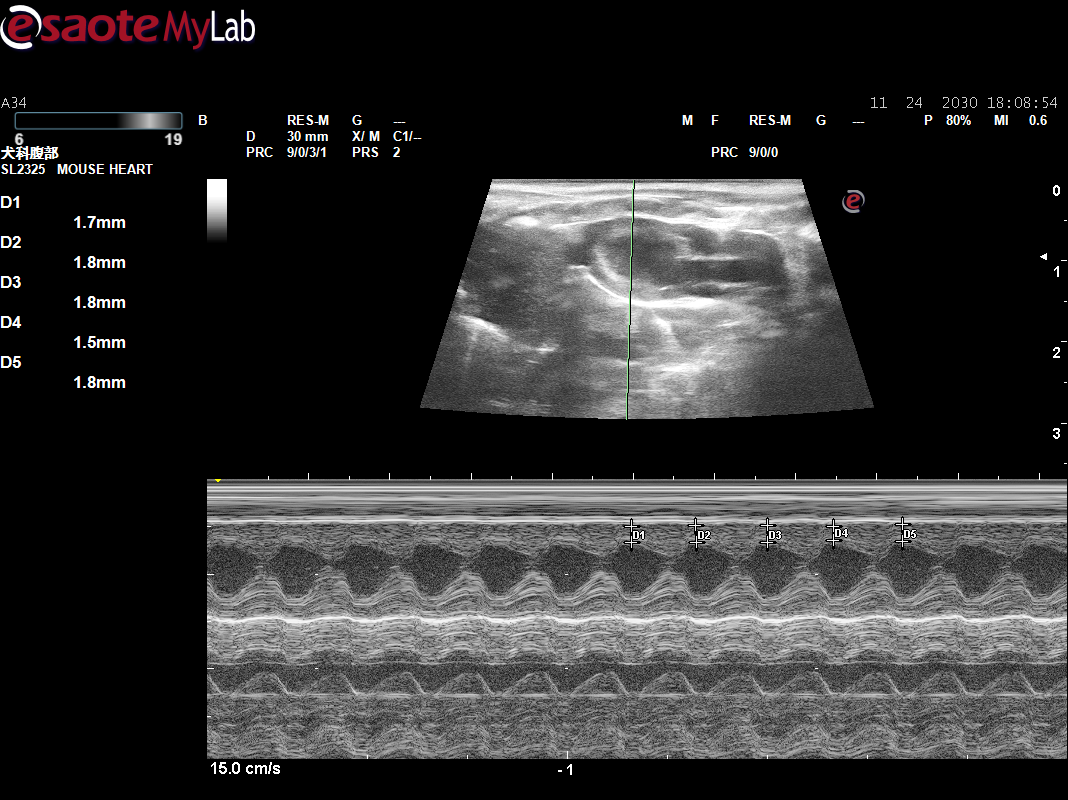

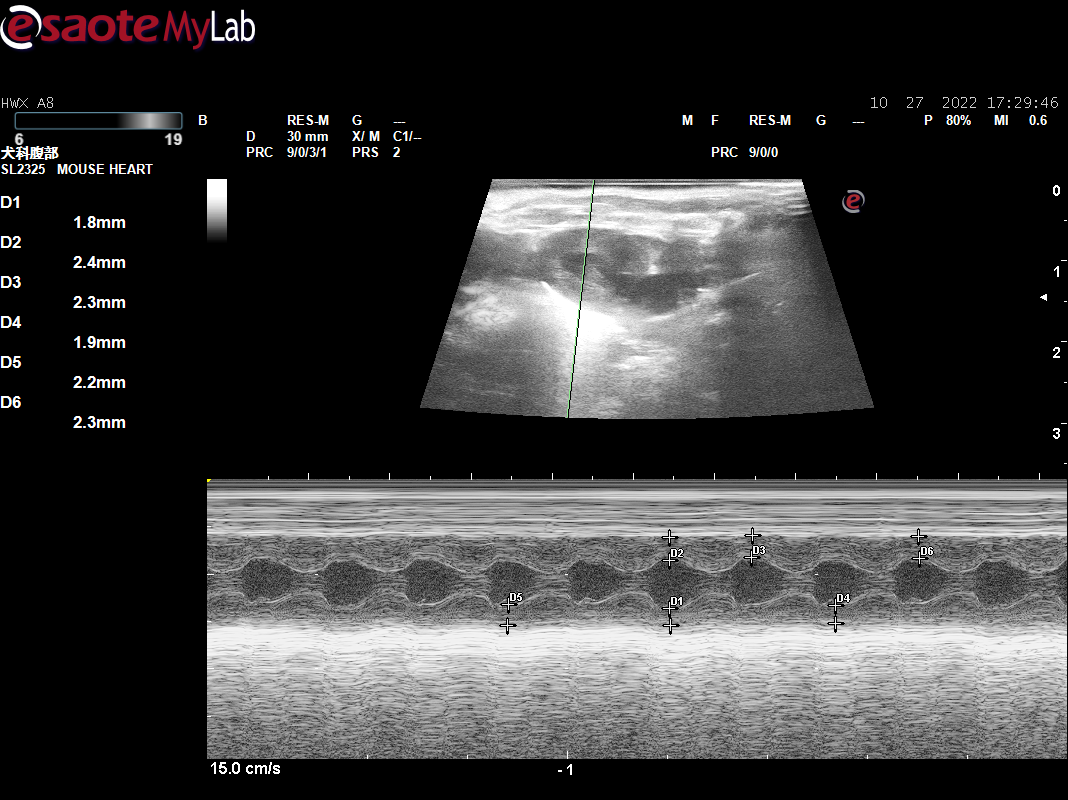

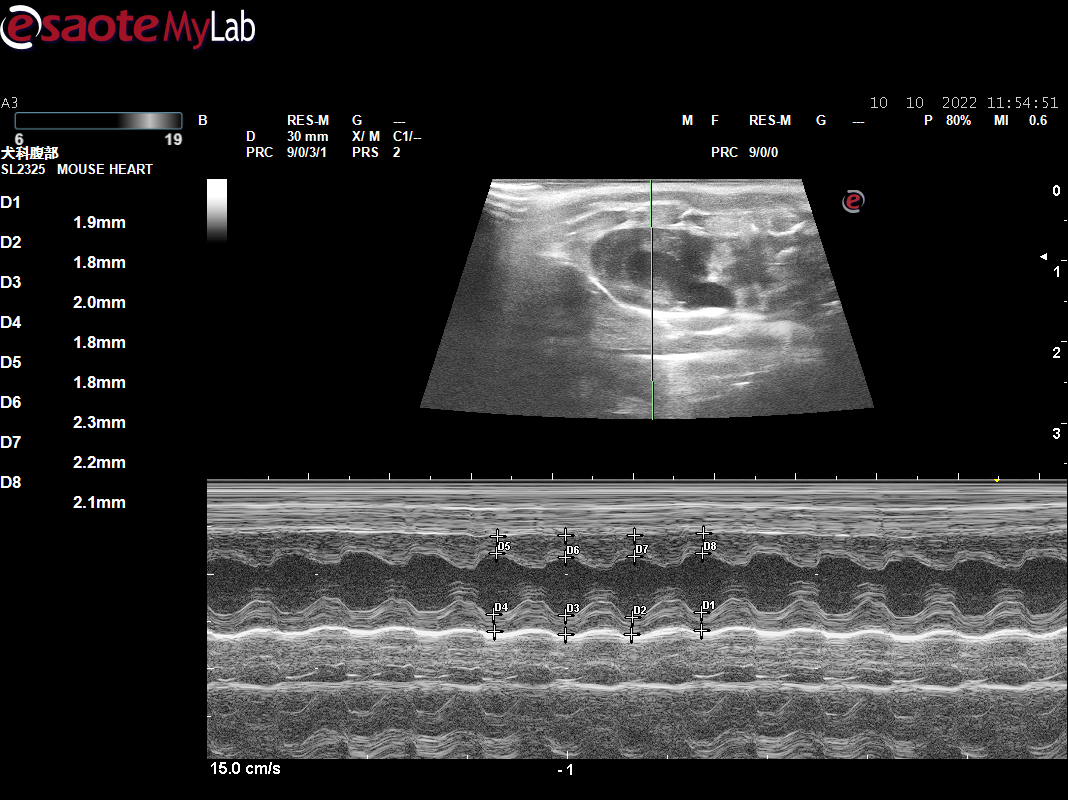

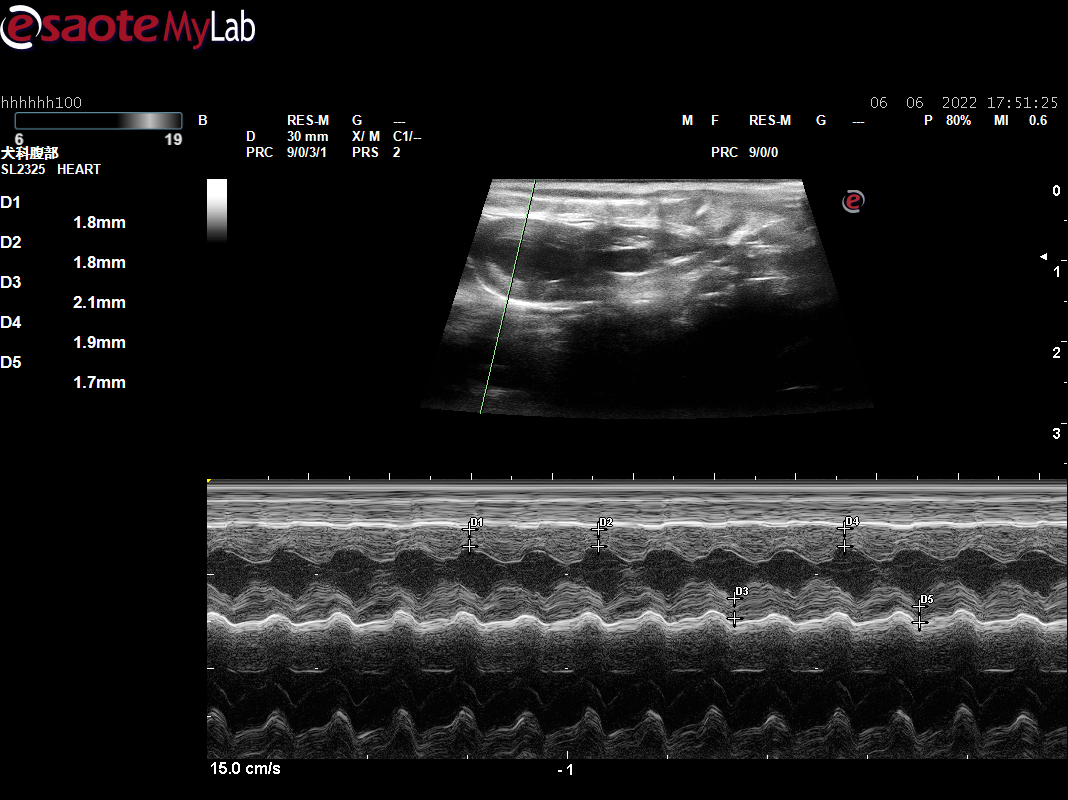

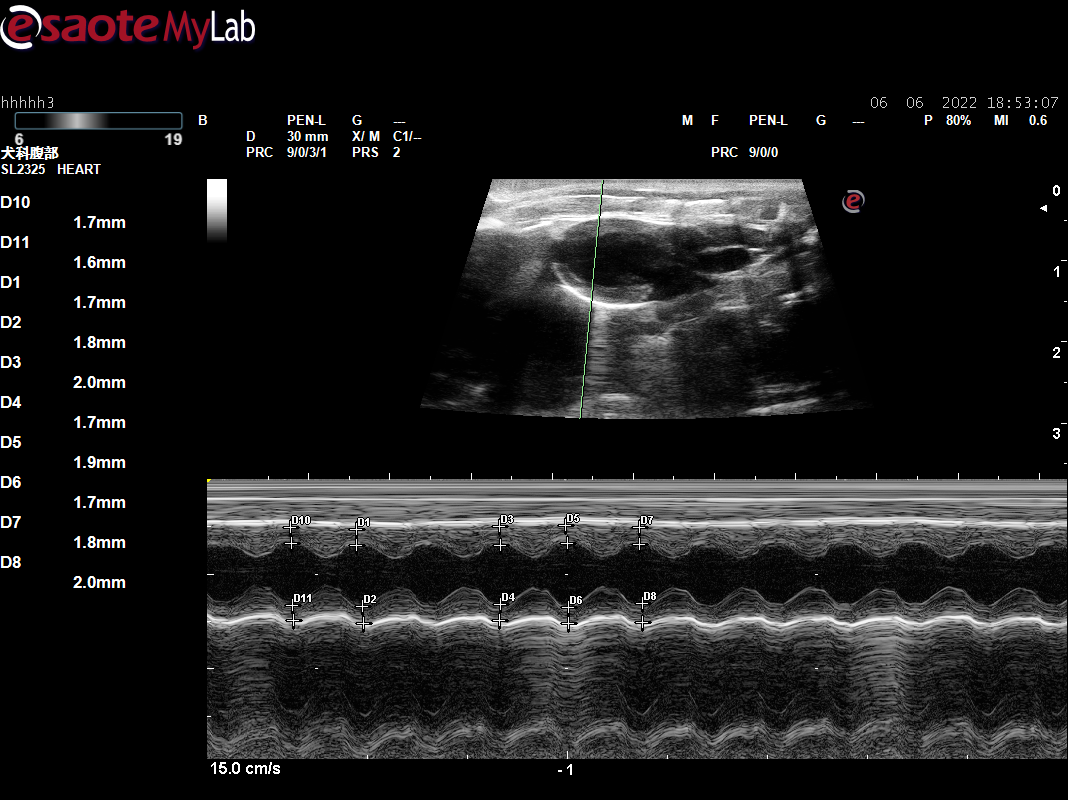

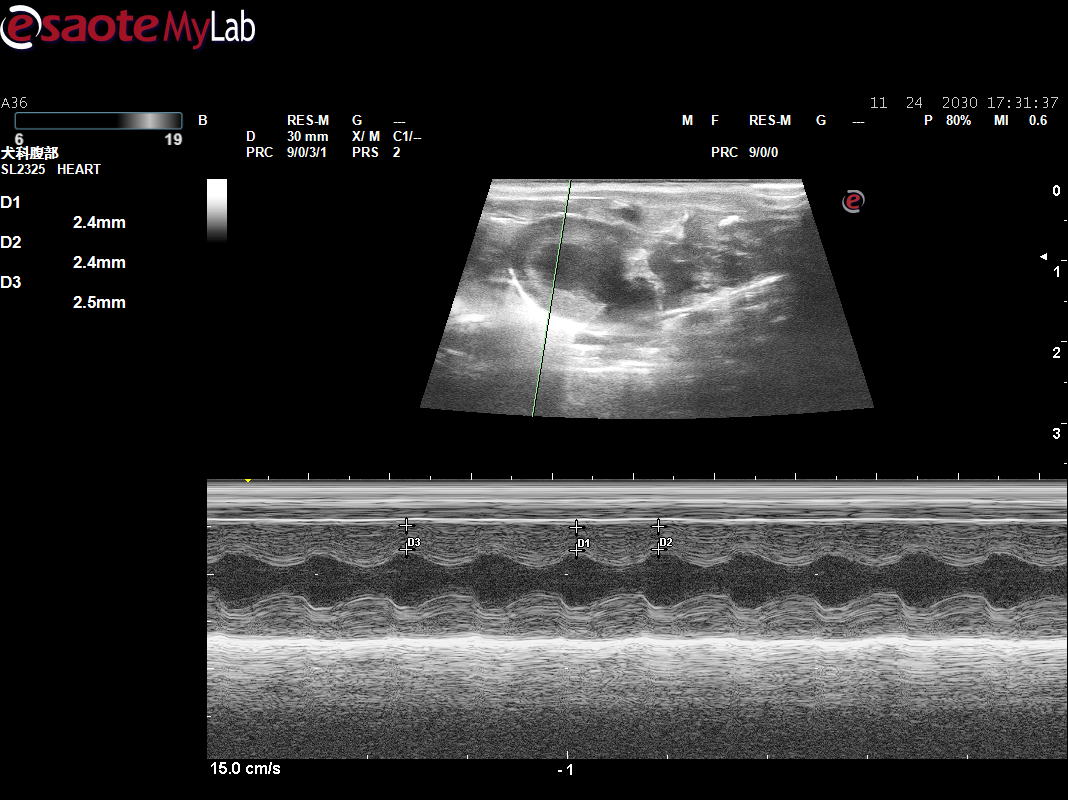


TAC+Semaglutide-IVSD


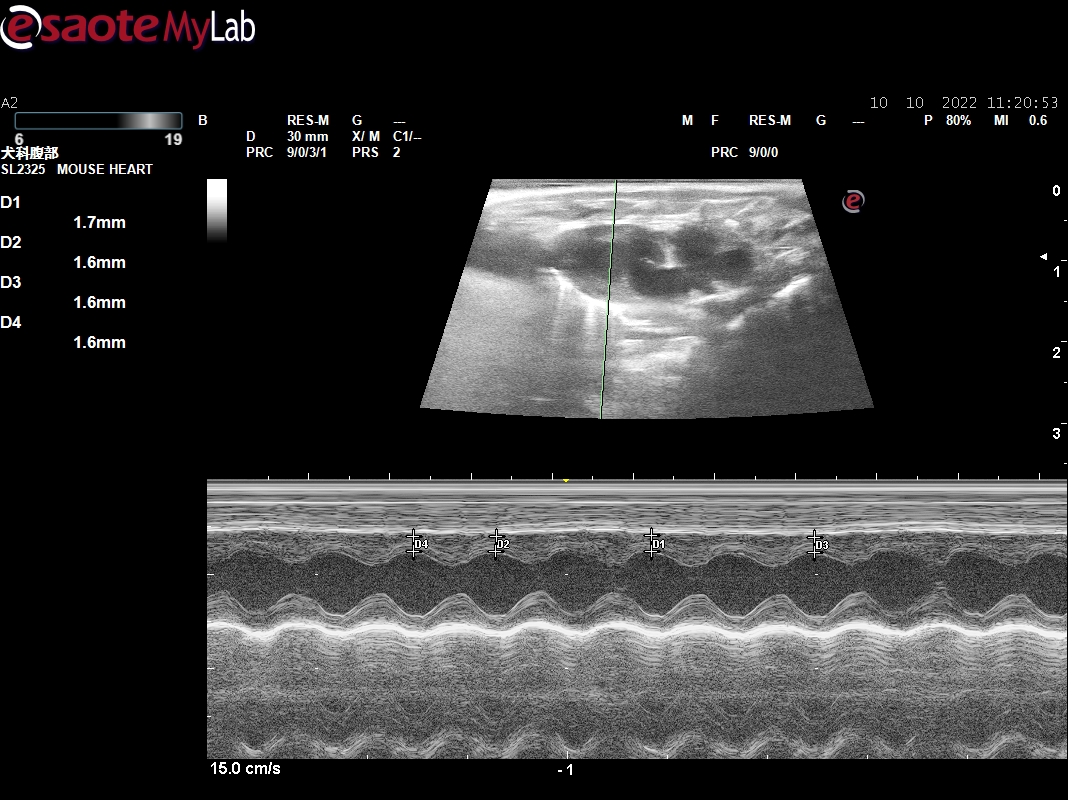

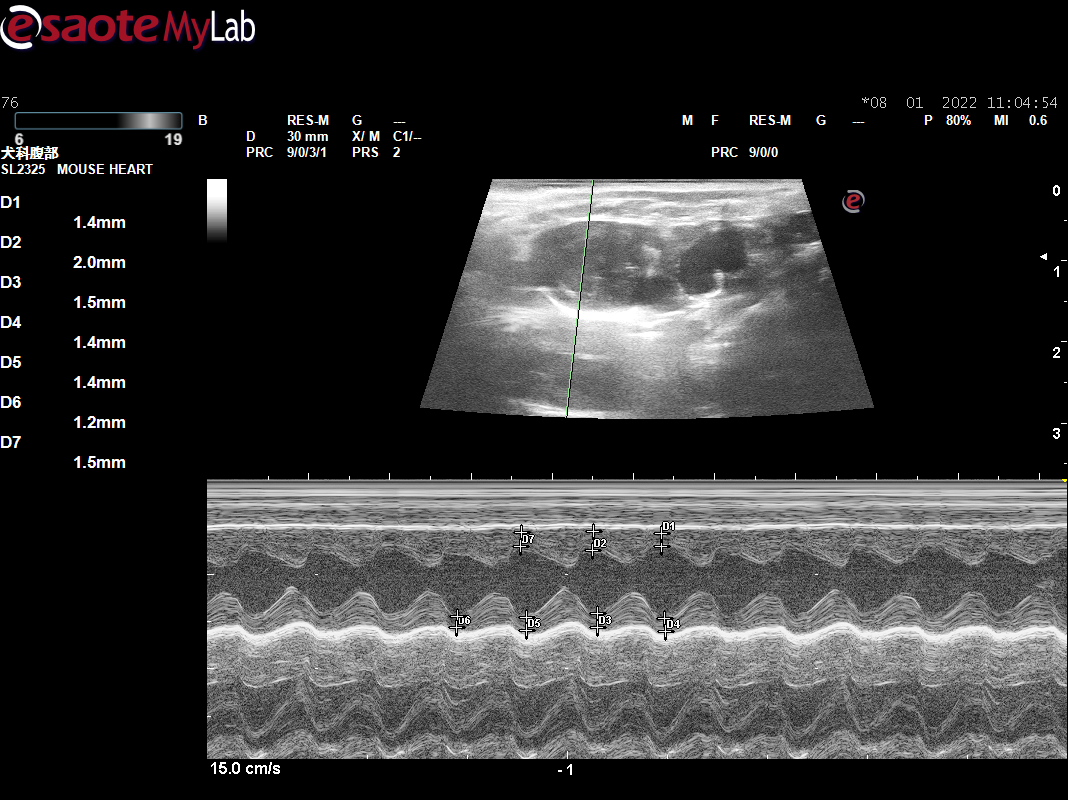

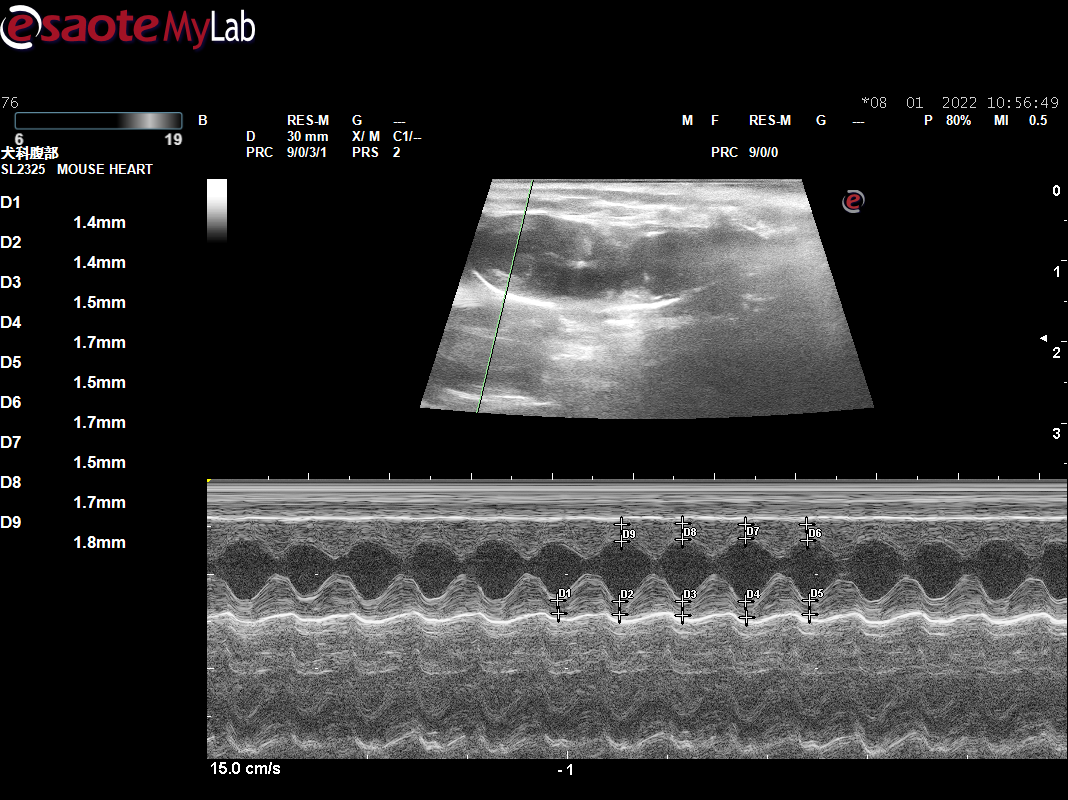

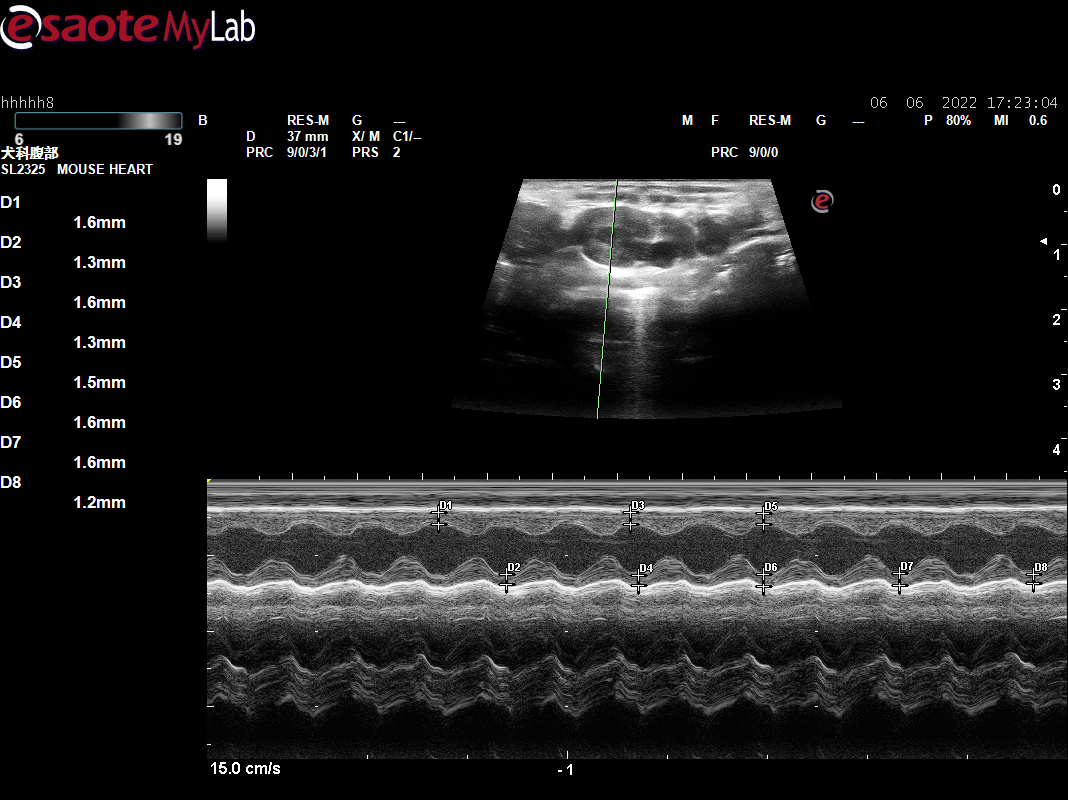

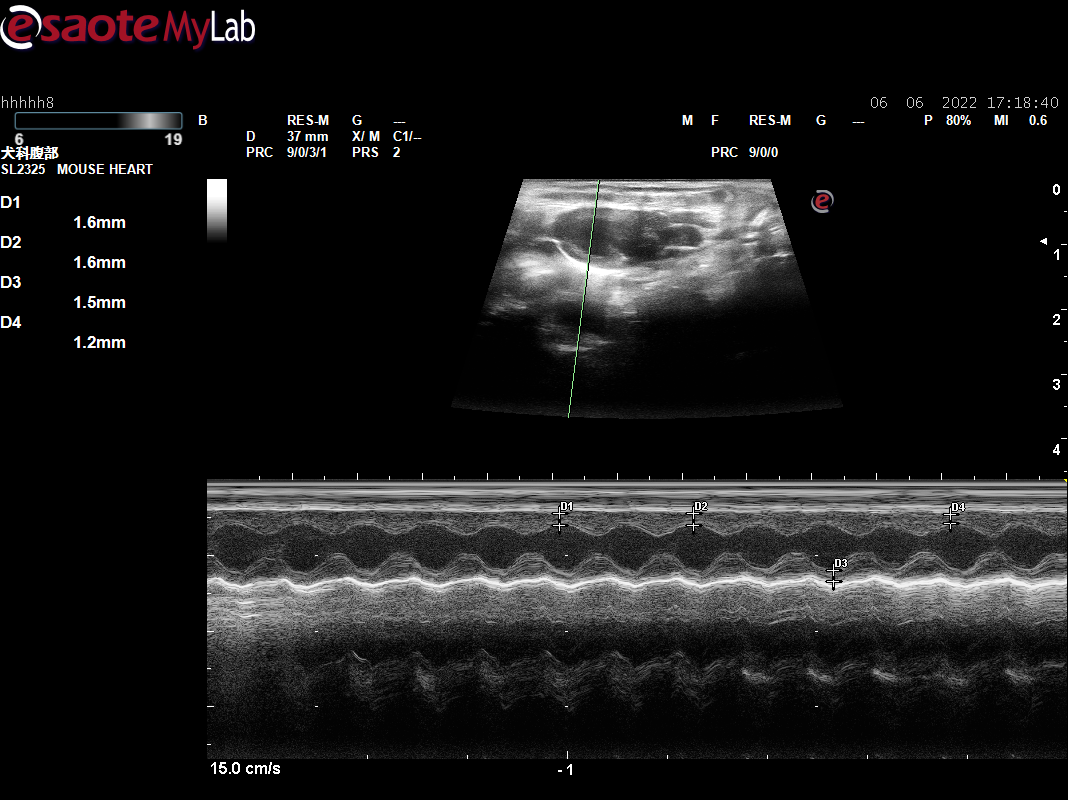

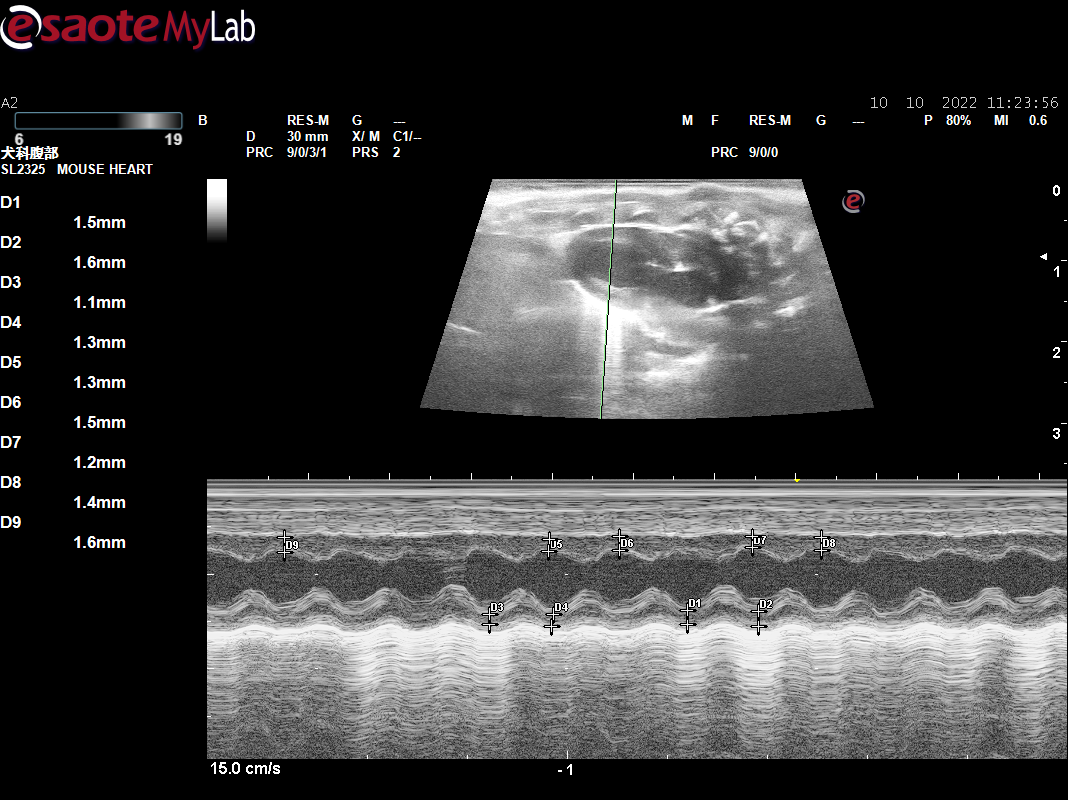


TAC+Semaglutide+HCQ-IVSD


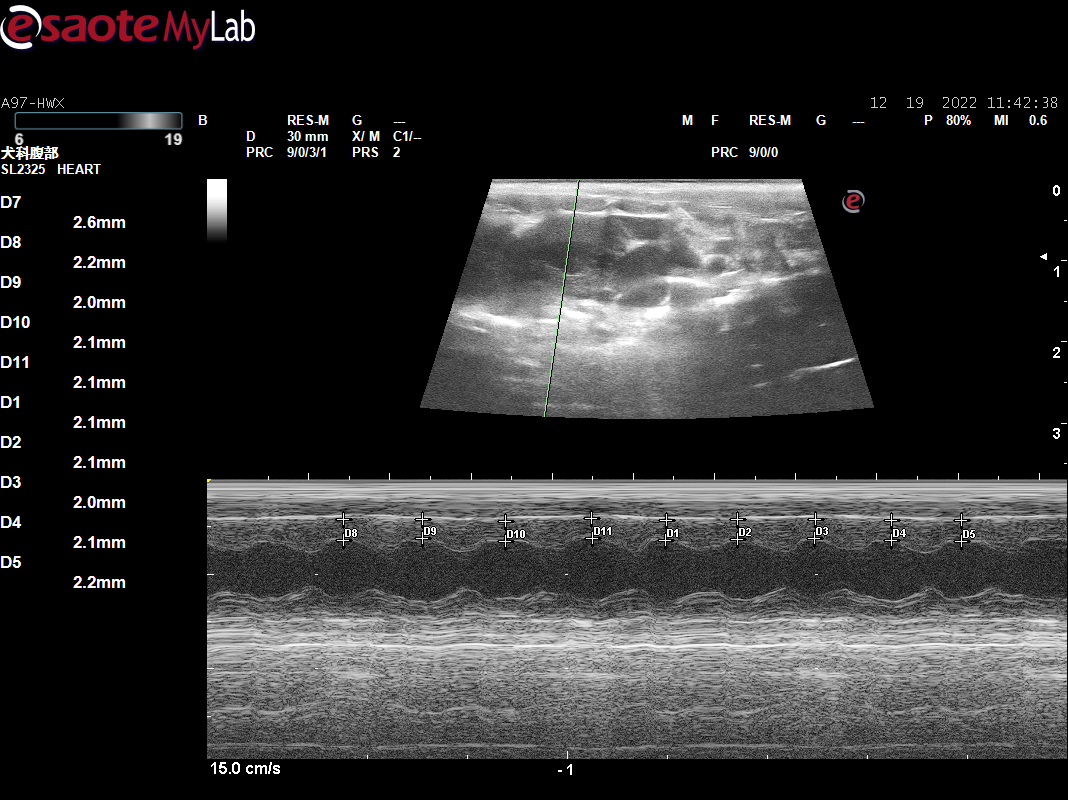

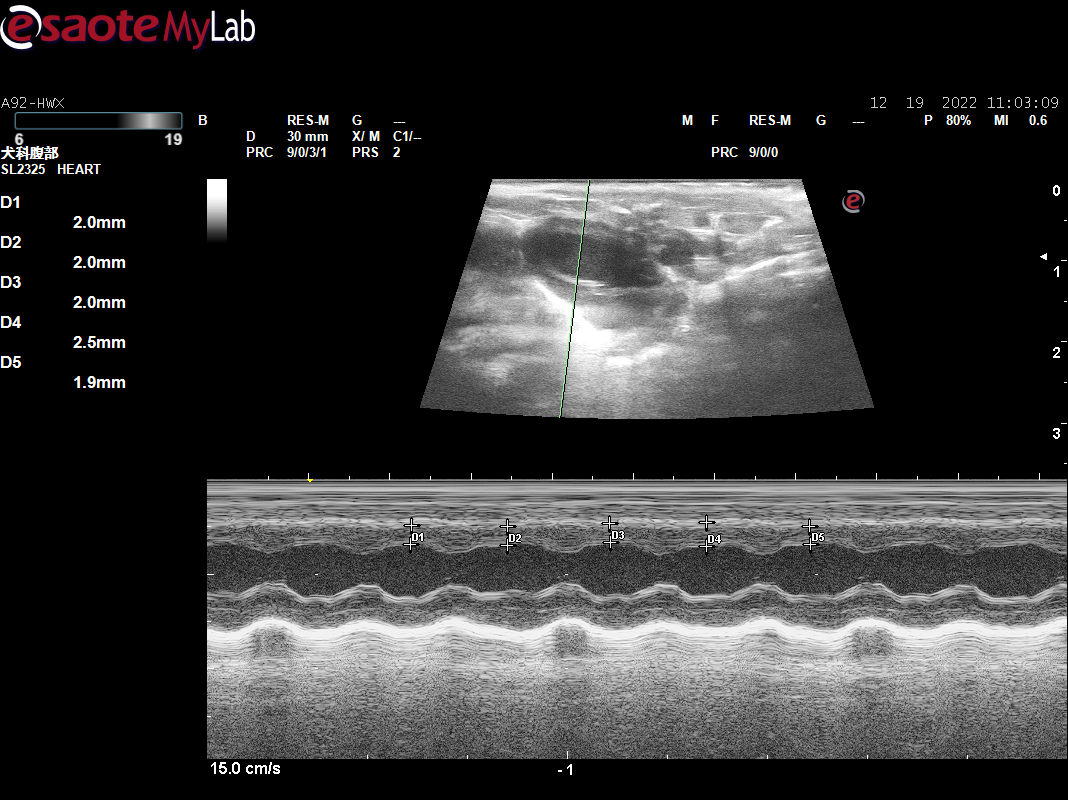

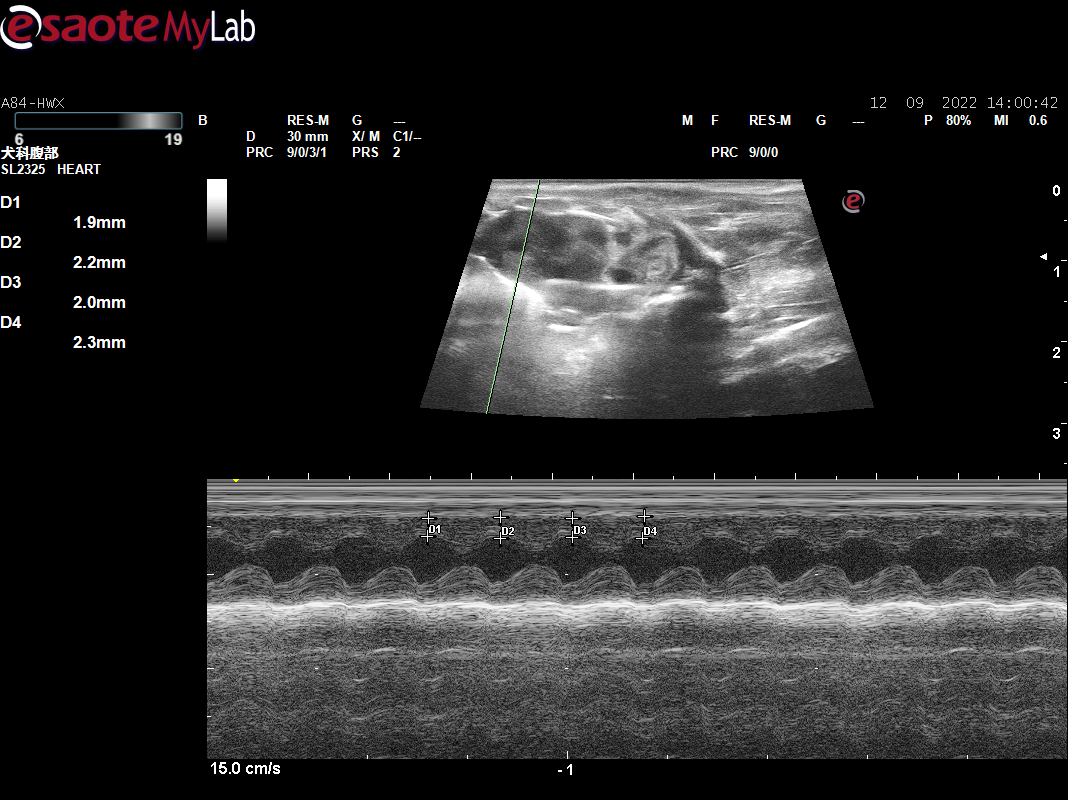

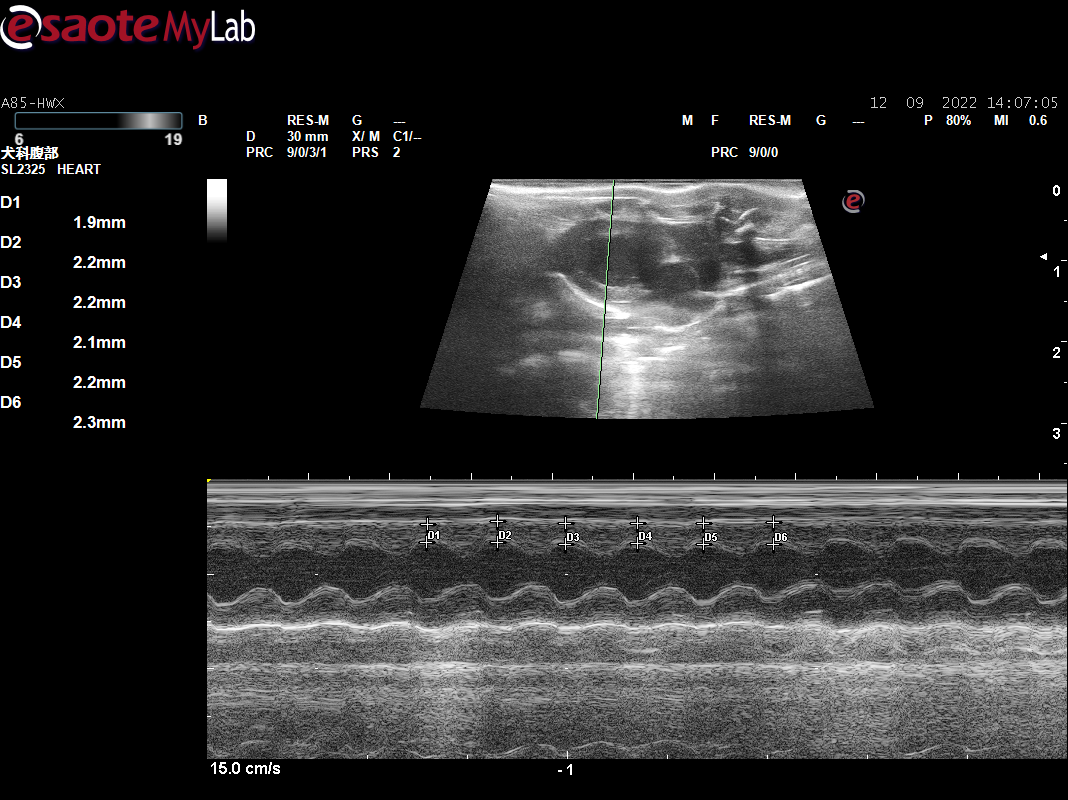

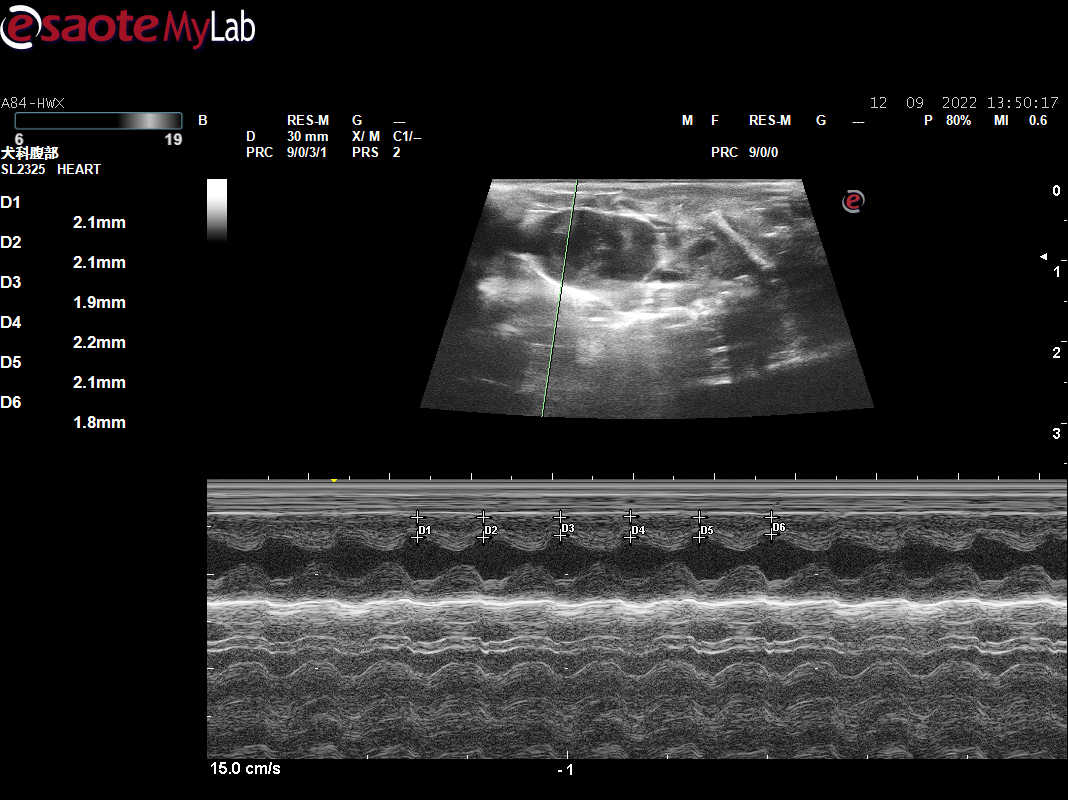

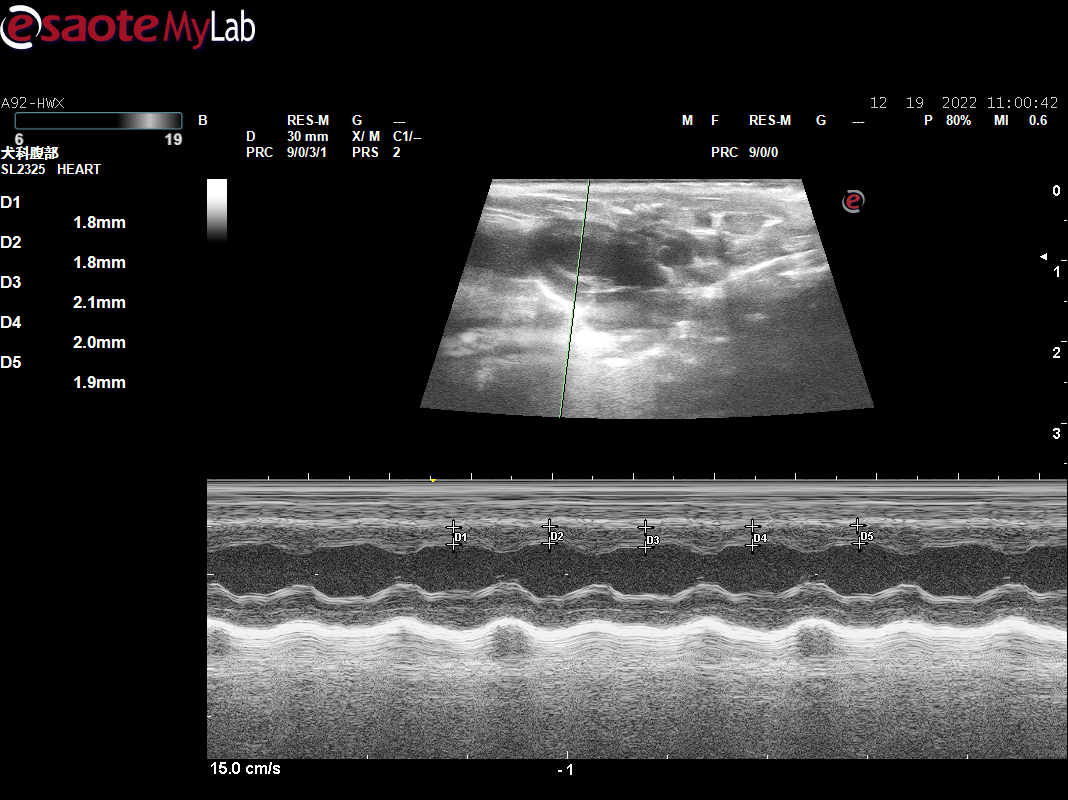

Supplement: Supplementary file 31 — Supplementary Information 31. [file 41598_2024_62465_MOESM31_ESM.docx]

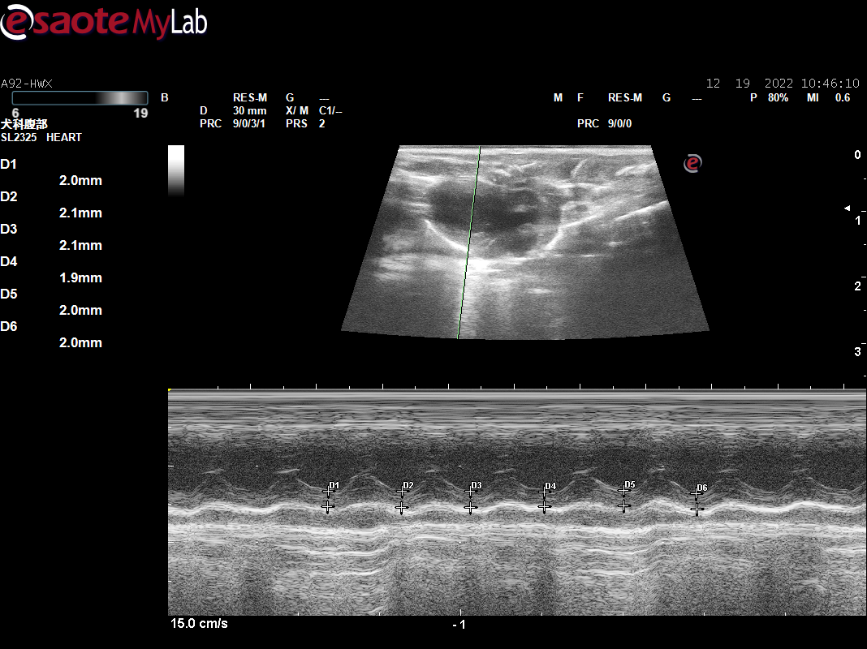

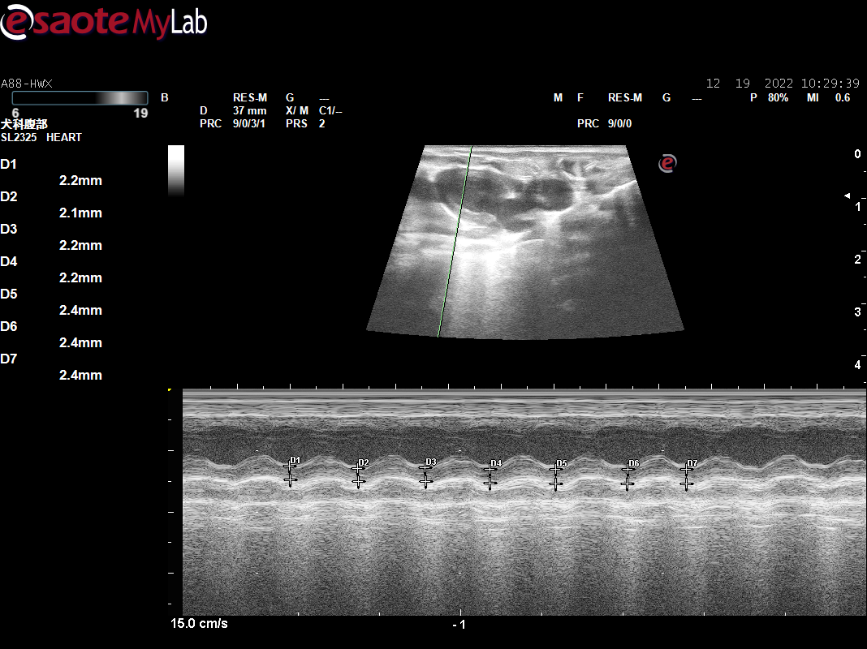

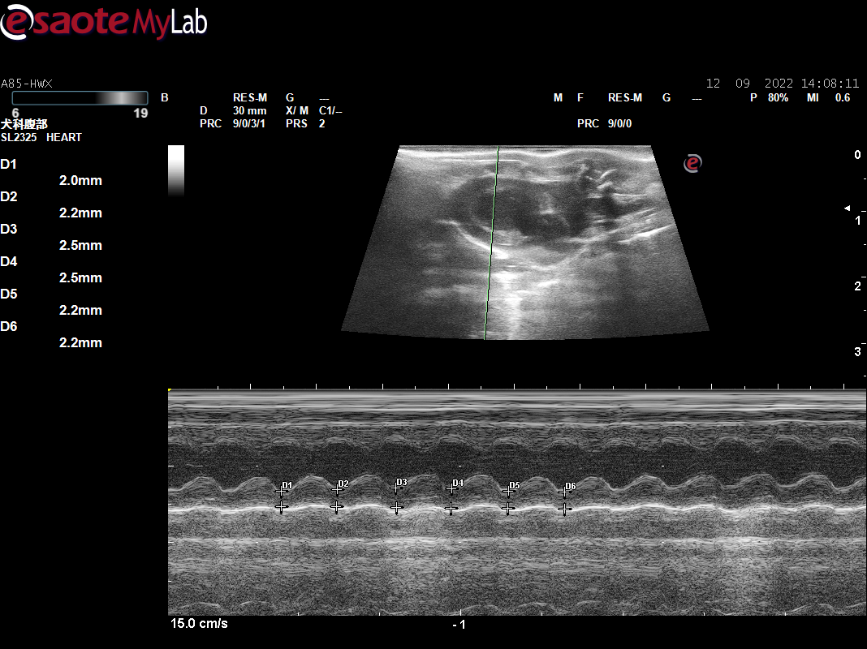

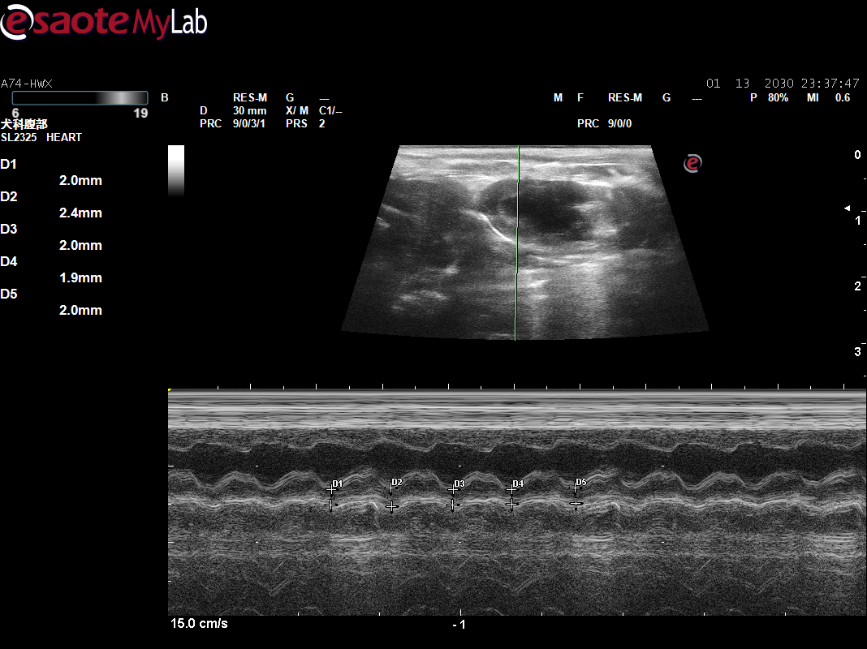

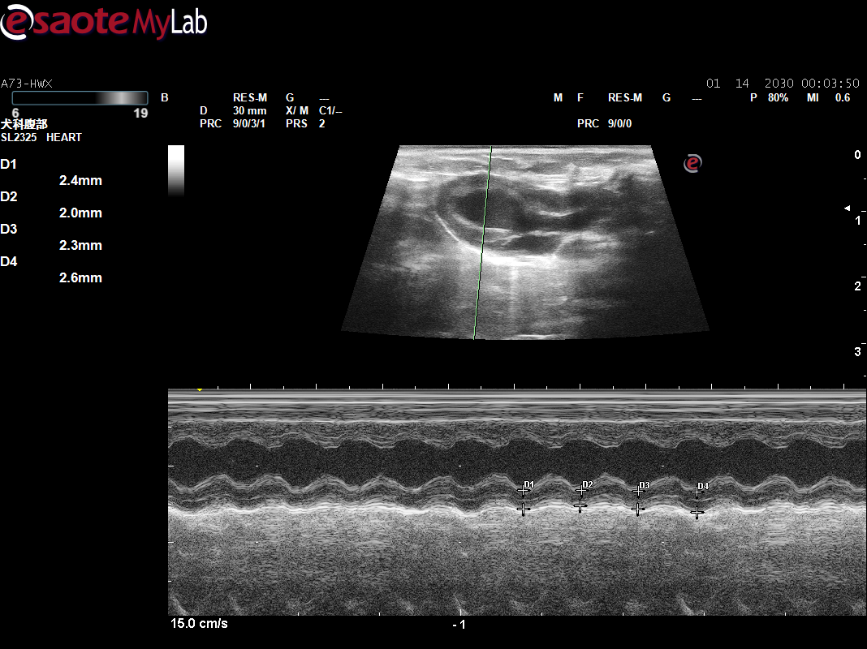

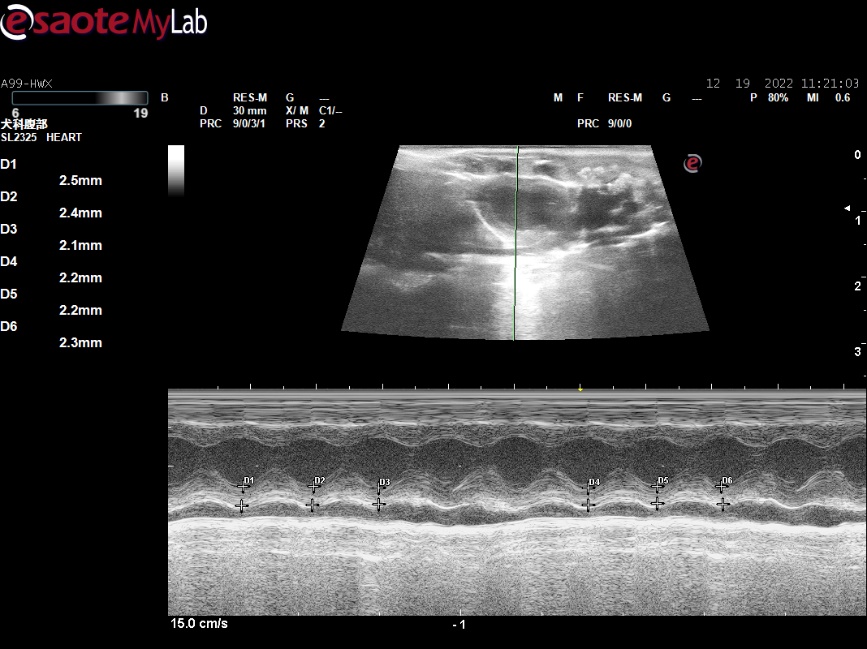


The red part of this picture is shown in our manuscript (Fig. 2E).

TAC+Semaglutide+HCQ

Supplement: Supplementary file 35 — Supplementary Information 35. [file 41598_2024_62465_MOESM35_ESM.docx]
