# Supplementary material for: Semaglutide ameliorates pressure overload-induced cardiac hypertrophy by improving cardiac mitophagy to suppress the activation of NLRP3 inflammasome
Source: Sci Rep. 2024 May 23;14:11824. doi: 10.1038/s41598-024-62465-6 (PMC11116553; doi:10.1038/s41598-024-62465-6)

Aorta without constriction identified by echocardiology


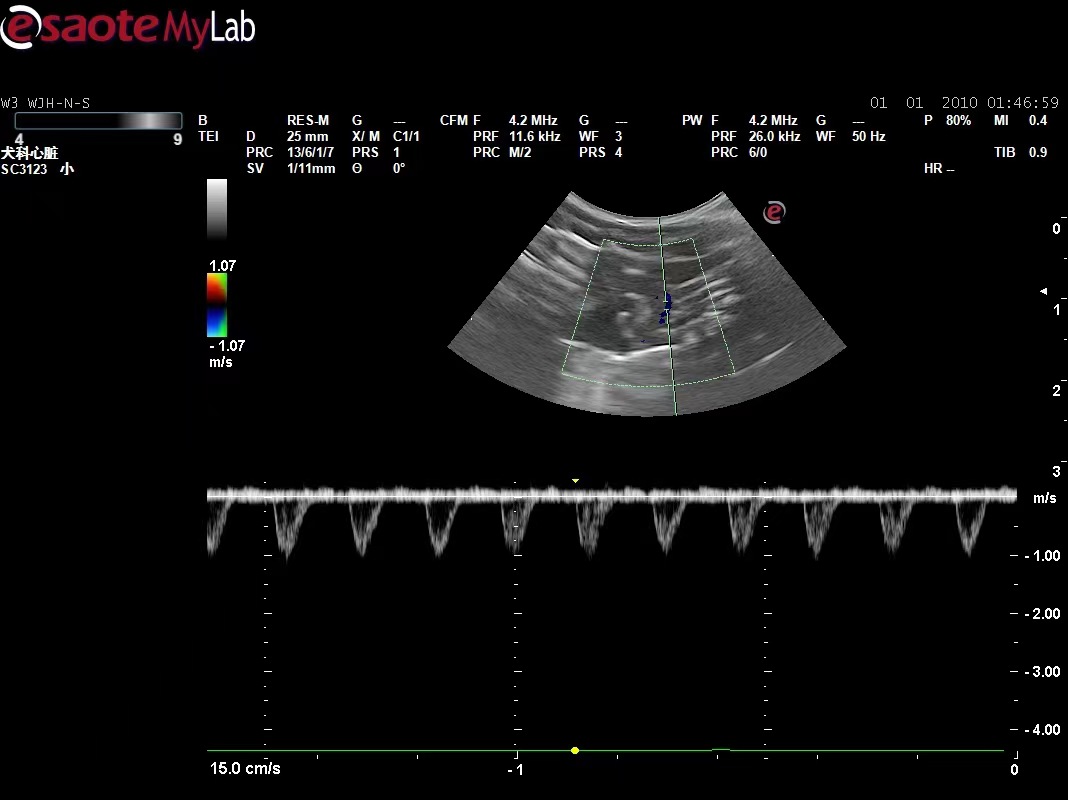


Aorta with constriction identified by echocardiology


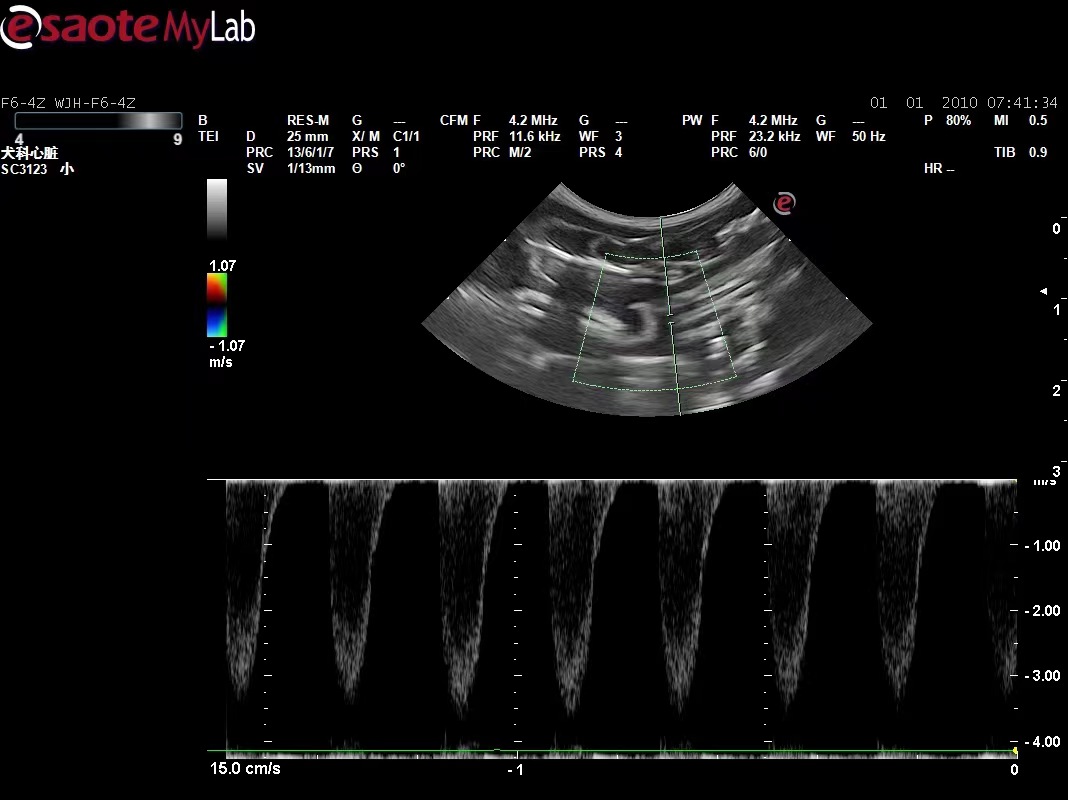


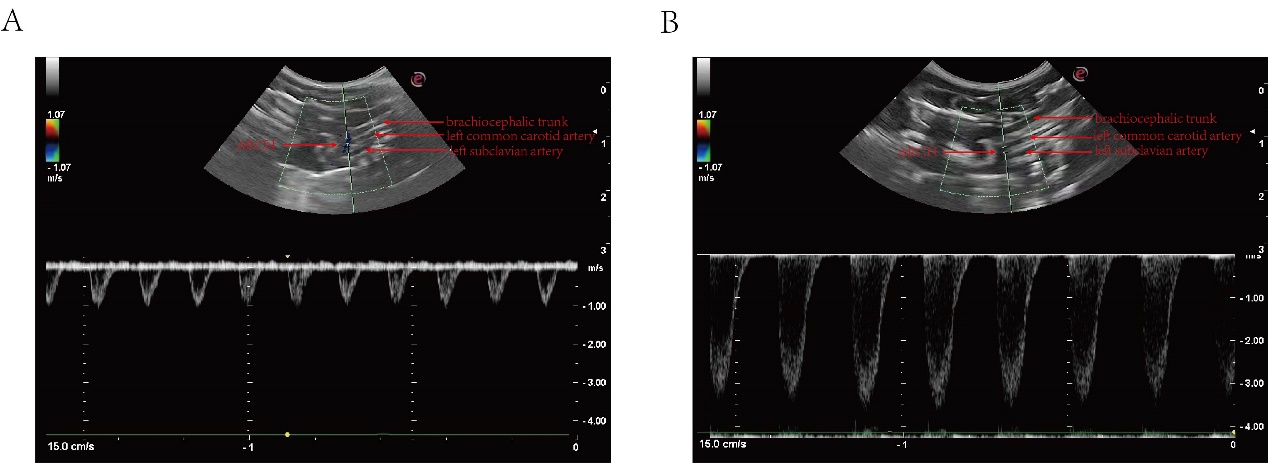

Supplement: Supplementary file 9 — Supplementary Information 9. [file 41598_2024_62465_MOESM9_ESM.docx]
