# Supplementary material for: Semaglutide ameliorates pressure overload-induced cardiac hypertrophy by improving cardiac mitophagy to suppress the activation of NLRP3 inflammasome
Source: Sci Rep. 2024 May 23;14:11824. doi: 10.1038/s41598-024-62465-6 (PMC11116553; doi:10.1038/s41598-024-62465-6)
Supplement: Supplementary file 11 — Supplementary Information 11. [file 41598_2024_62465_MOESM11_ESM.docx]

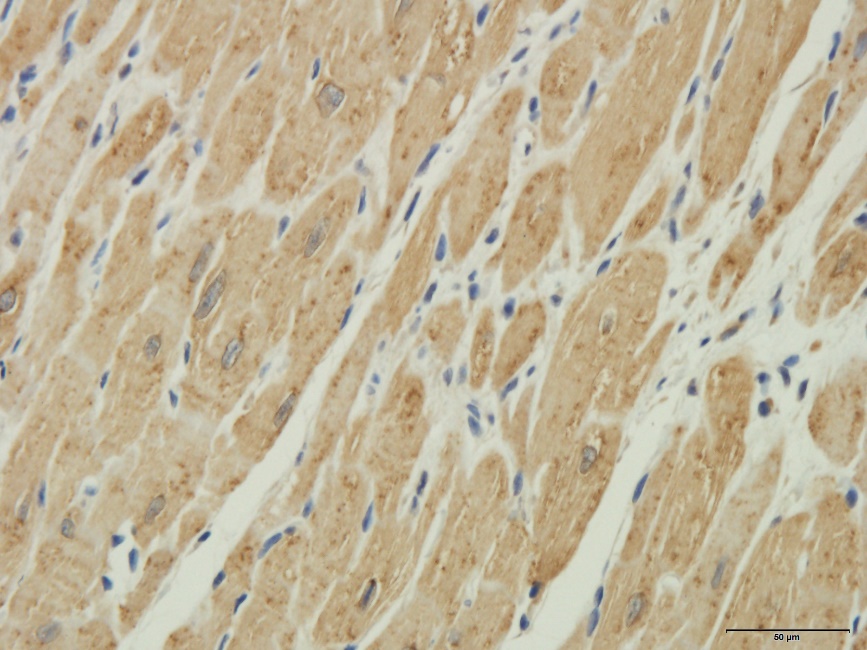

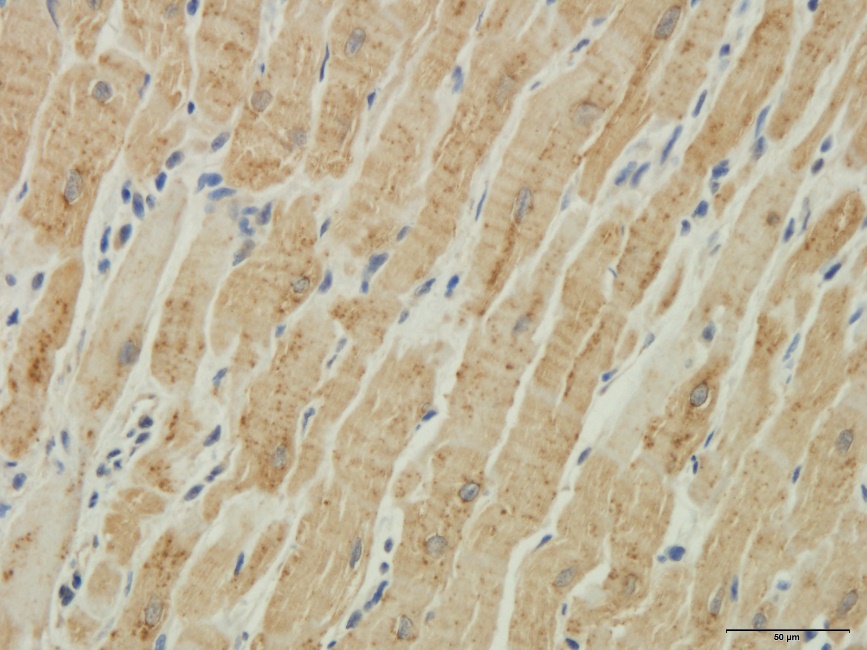

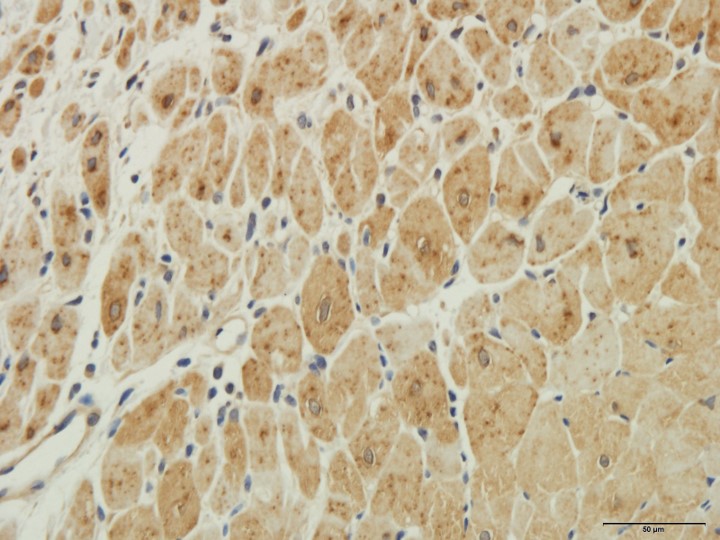

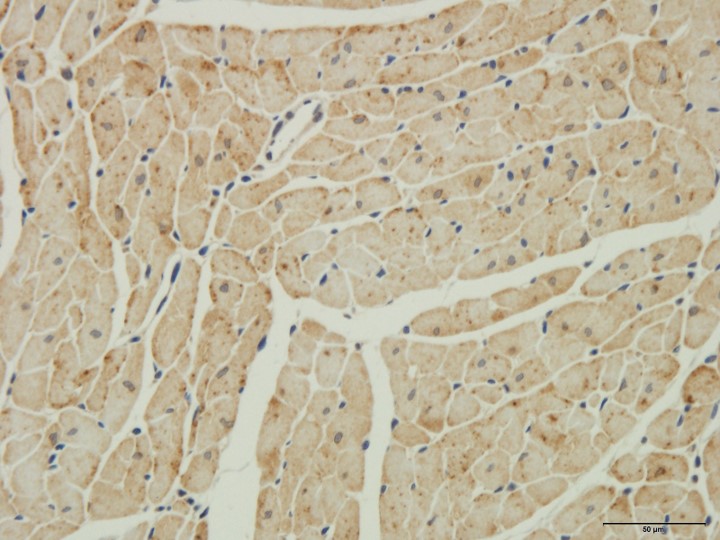

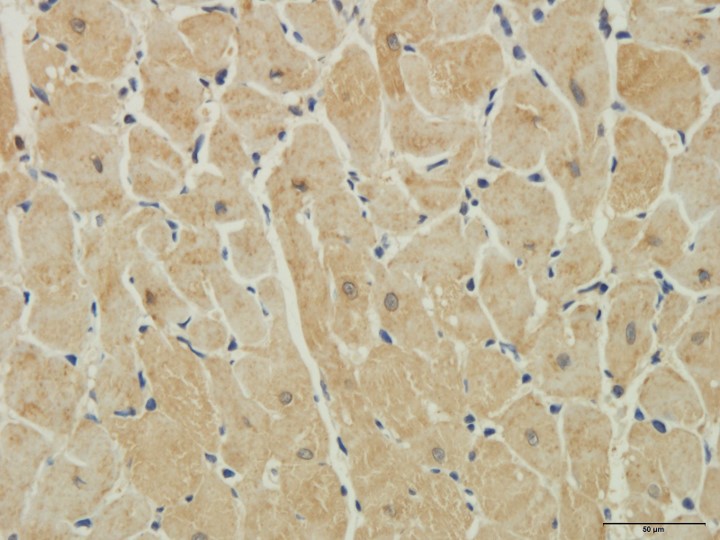

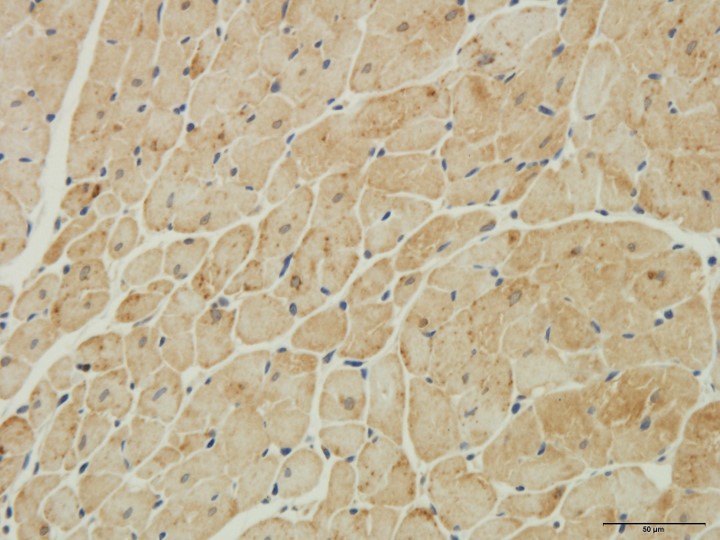


This picture was used for statistics.

This picture was used for statistics.

This picture was used for statistics.

ANP-TAC

This picture was used for statistics.

This picture was used for statistics.

This picture was showed in our manuscript (Fig. 3C).
