# Supplementary material for: Semaglutide ameliorates pressure overload-induced cardiac hypertrophy by improving cardiac mitophagy to suppress the activation of NLRP3 inflammasome
Source: Sci Rep. 2024 May 23;14:11824. doi: 10.1038/s41598-024-62465-6 (PMC11116553; doi:10.1038/s41598-024-62465-6)
Supplement: Supplementary file 16 — Supplementary Information 16. [file 41598_2024_62465_MOESM16_ESM.docx]

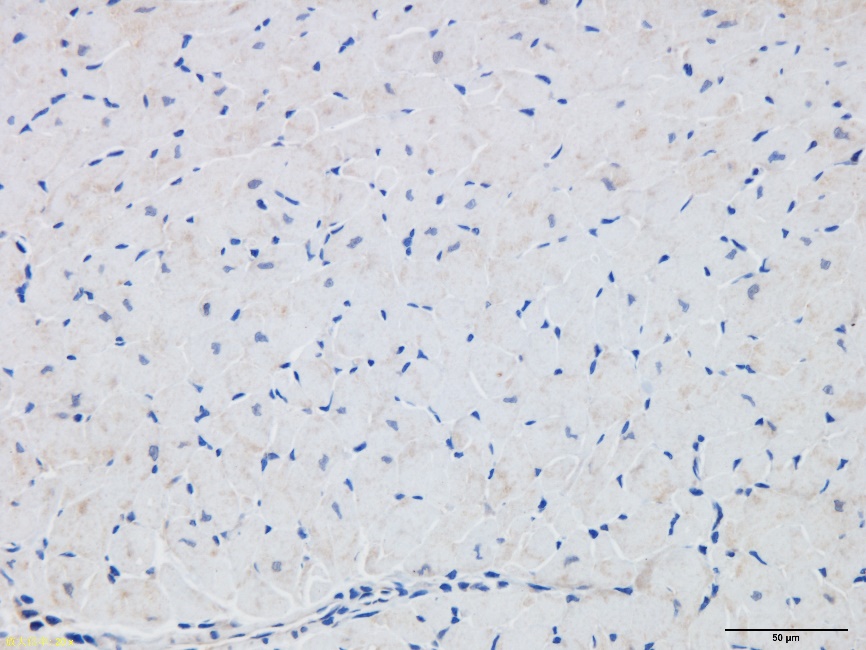

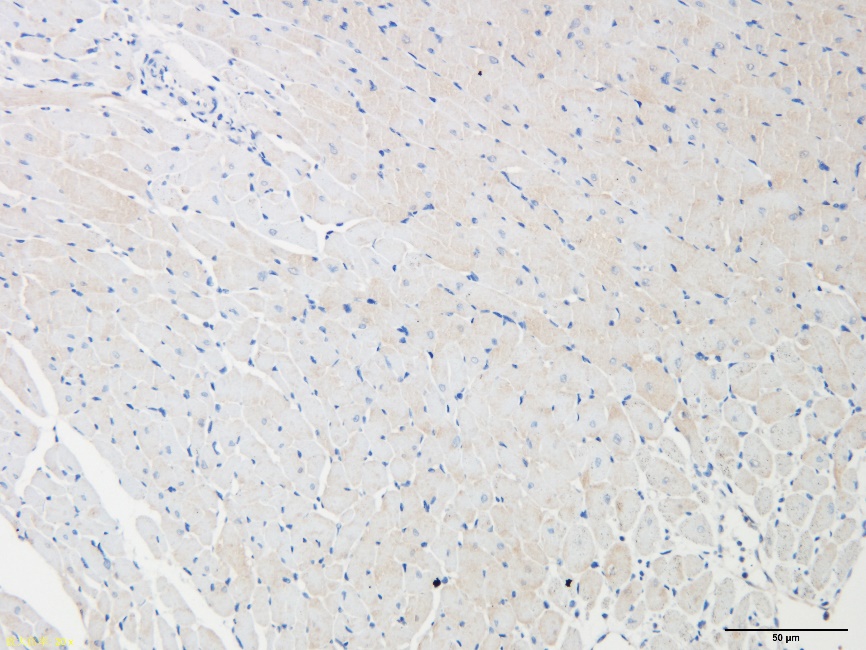

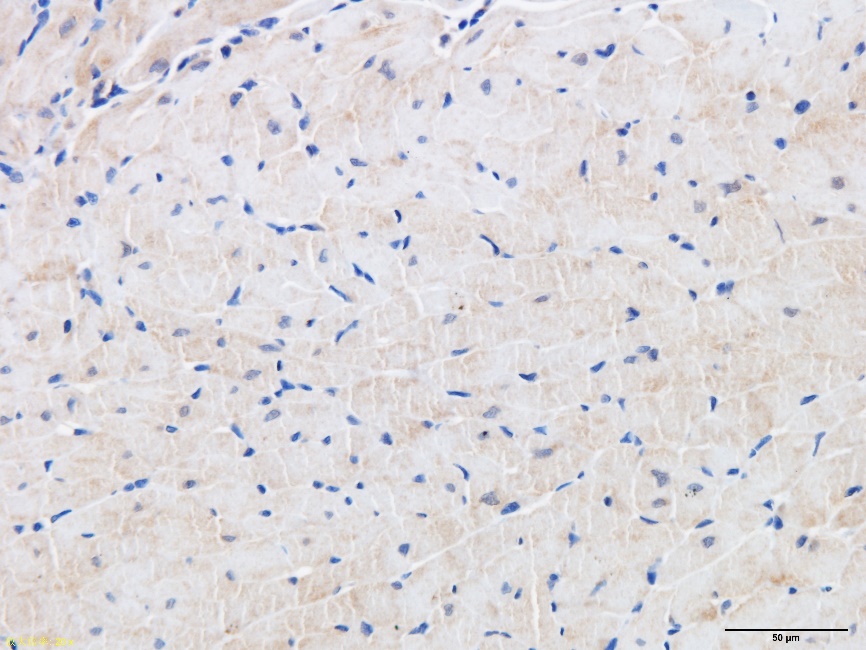

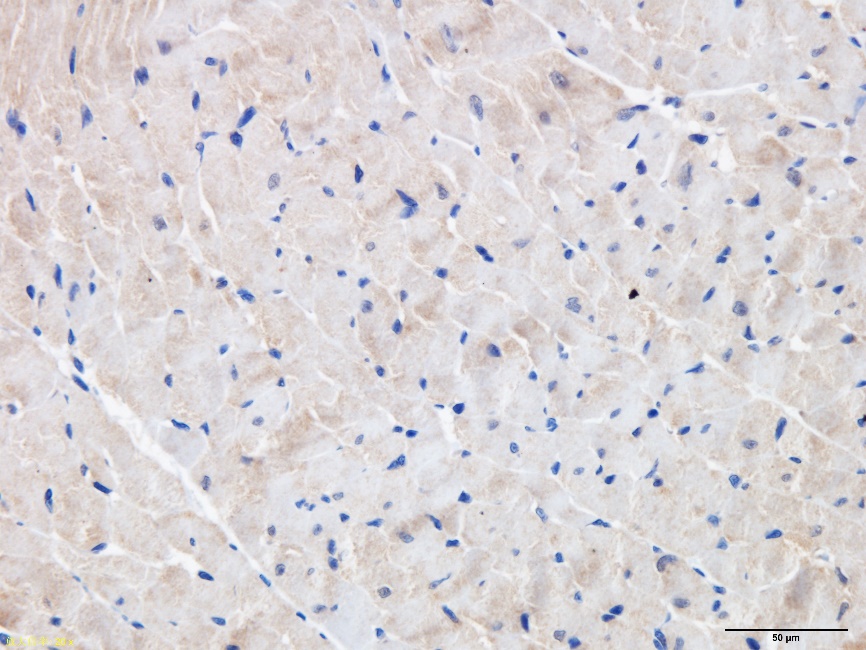

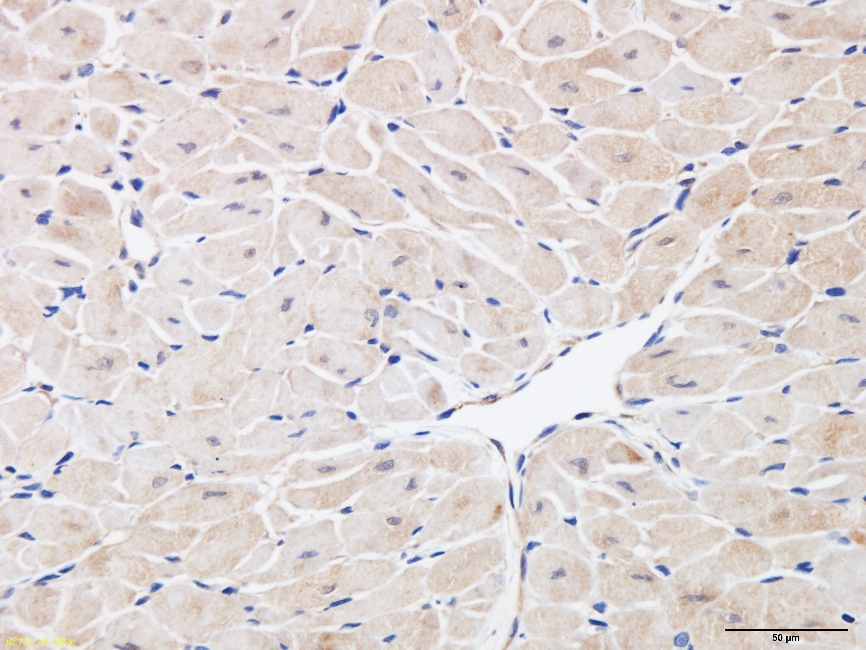

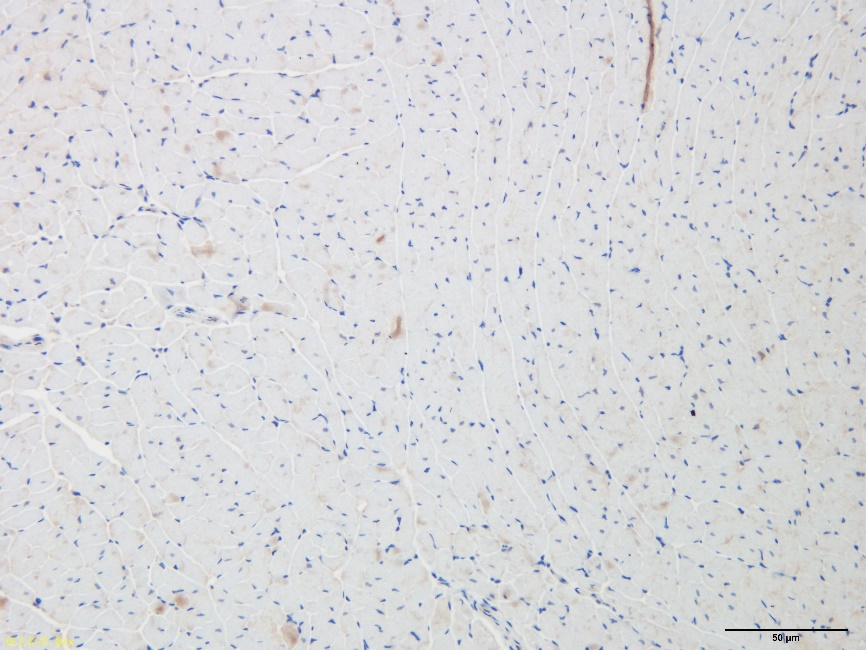


COXII-TAC+Semaglutide

This picture was used for statistics.

This picture was used for statistics.

This picture was used for statistics.

This picture was used for statistics.

This picture was used for statistics.

This picture was showed in our manuscript (Fig. 2B).
