# Supplementary material for: Semaglutide ameliorates pressure overload-induced cardiac hypertrophy by improving cardiac mitophagy to suppress the activation of NLRP3 inflammasome
Source: Sci Rep. 2024 May 23;14:11824. doi: 10.1038/s41598-024-62465-6 (PMC11116553; doi:10.1038/s41598-024-62465-6)

Interpretation of images with high contrast

1. Images of blots of redone


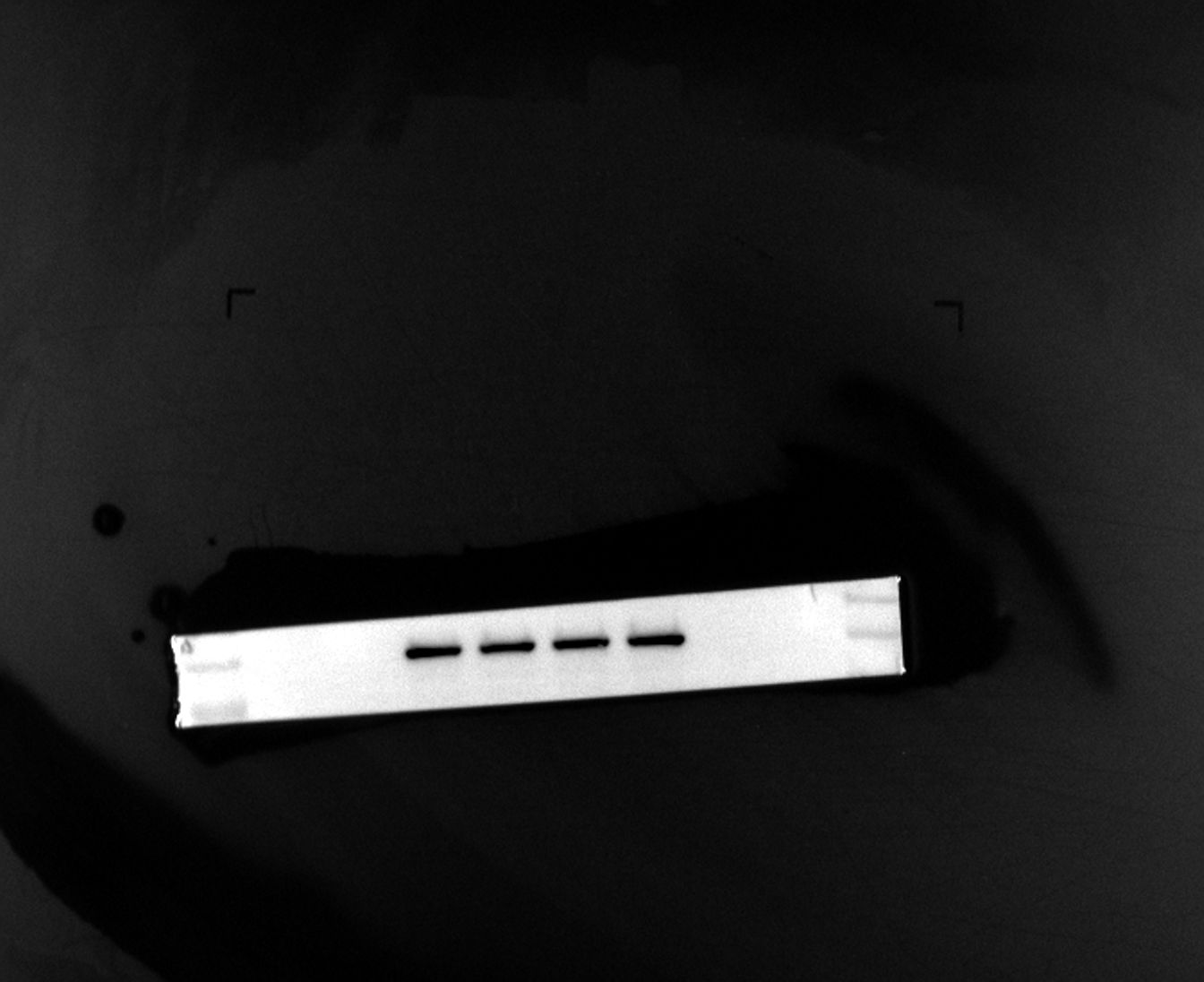


GAPDH-1 redone


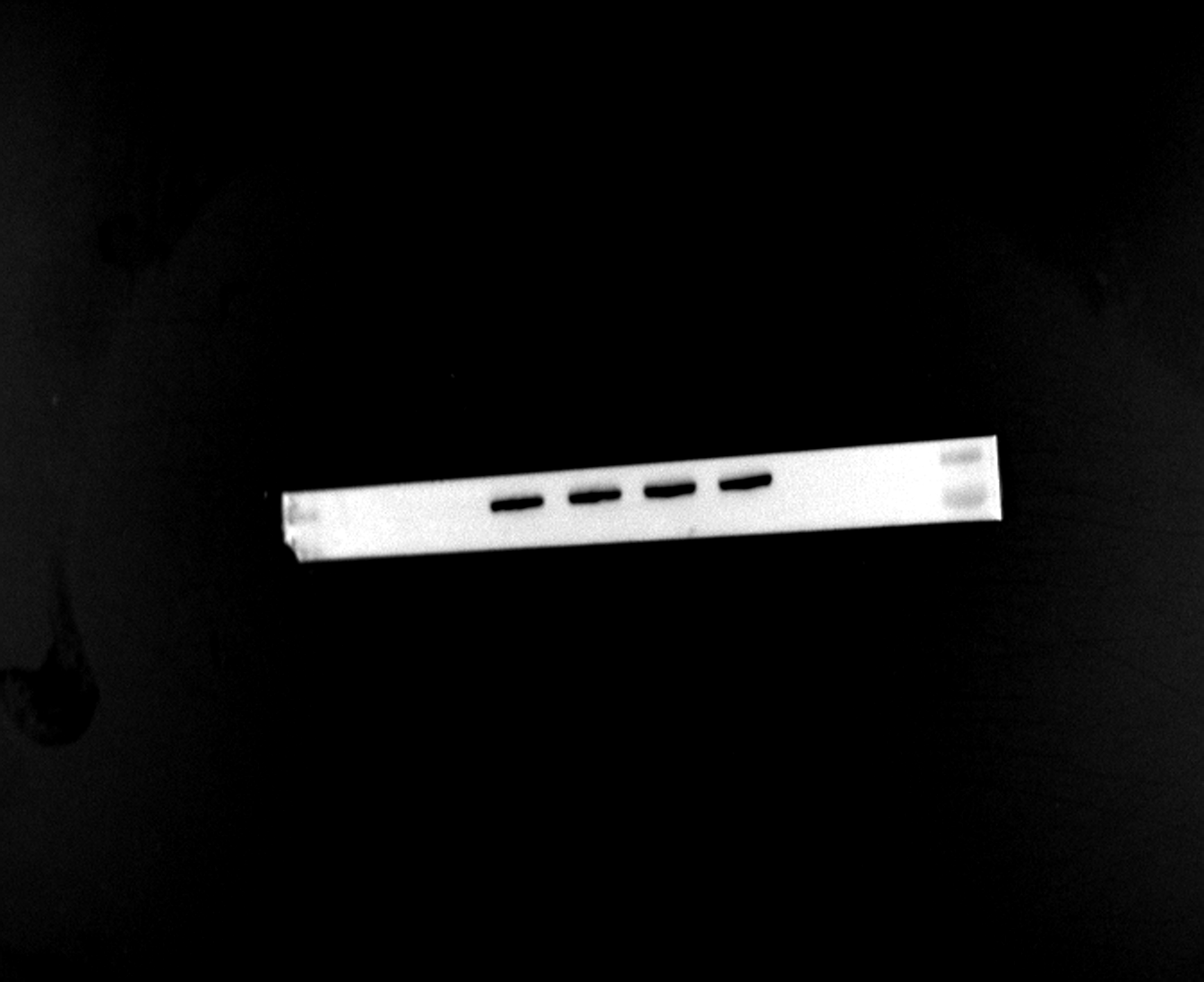


GAPDH-2 redone


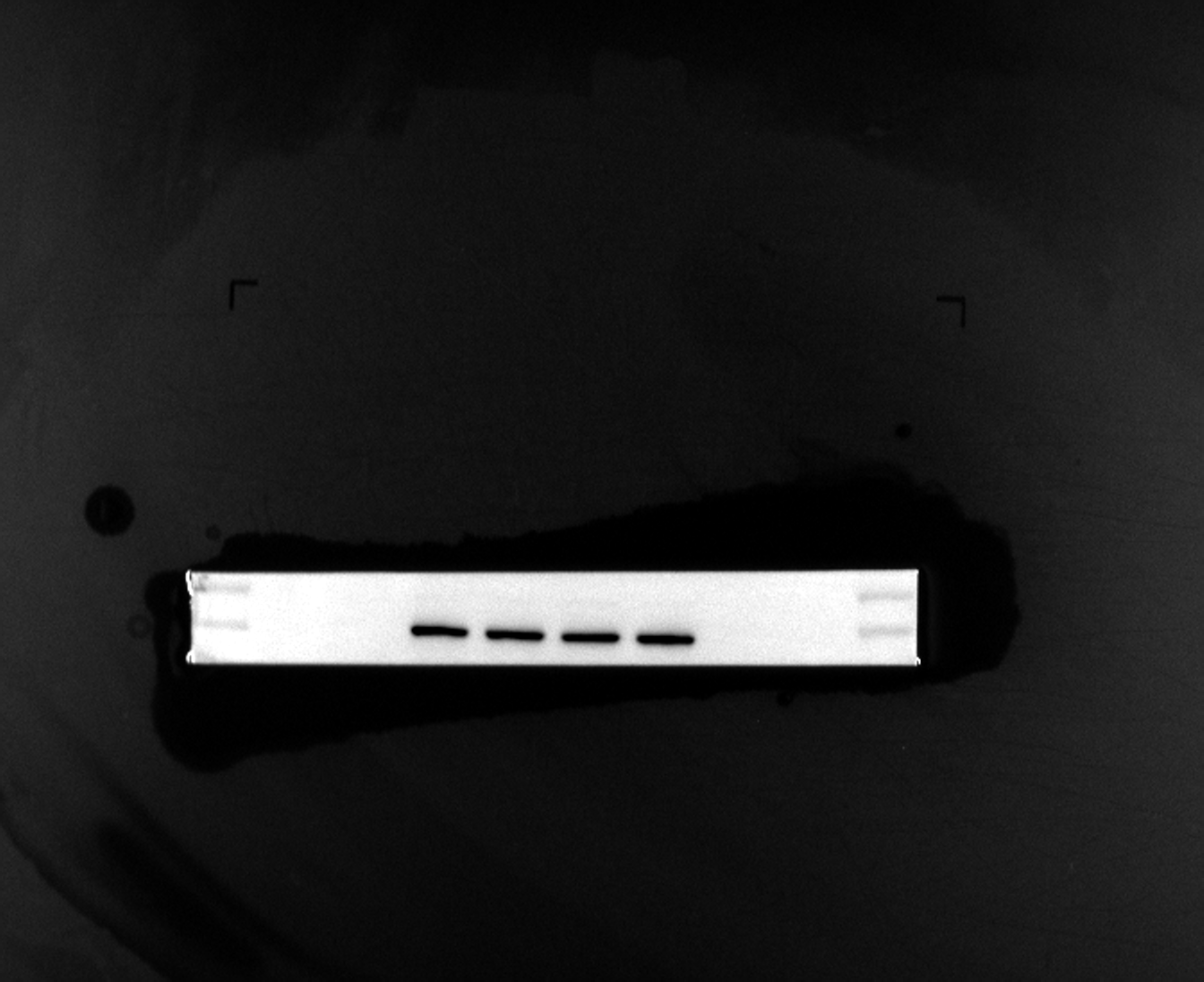


GAPDH-3 redone


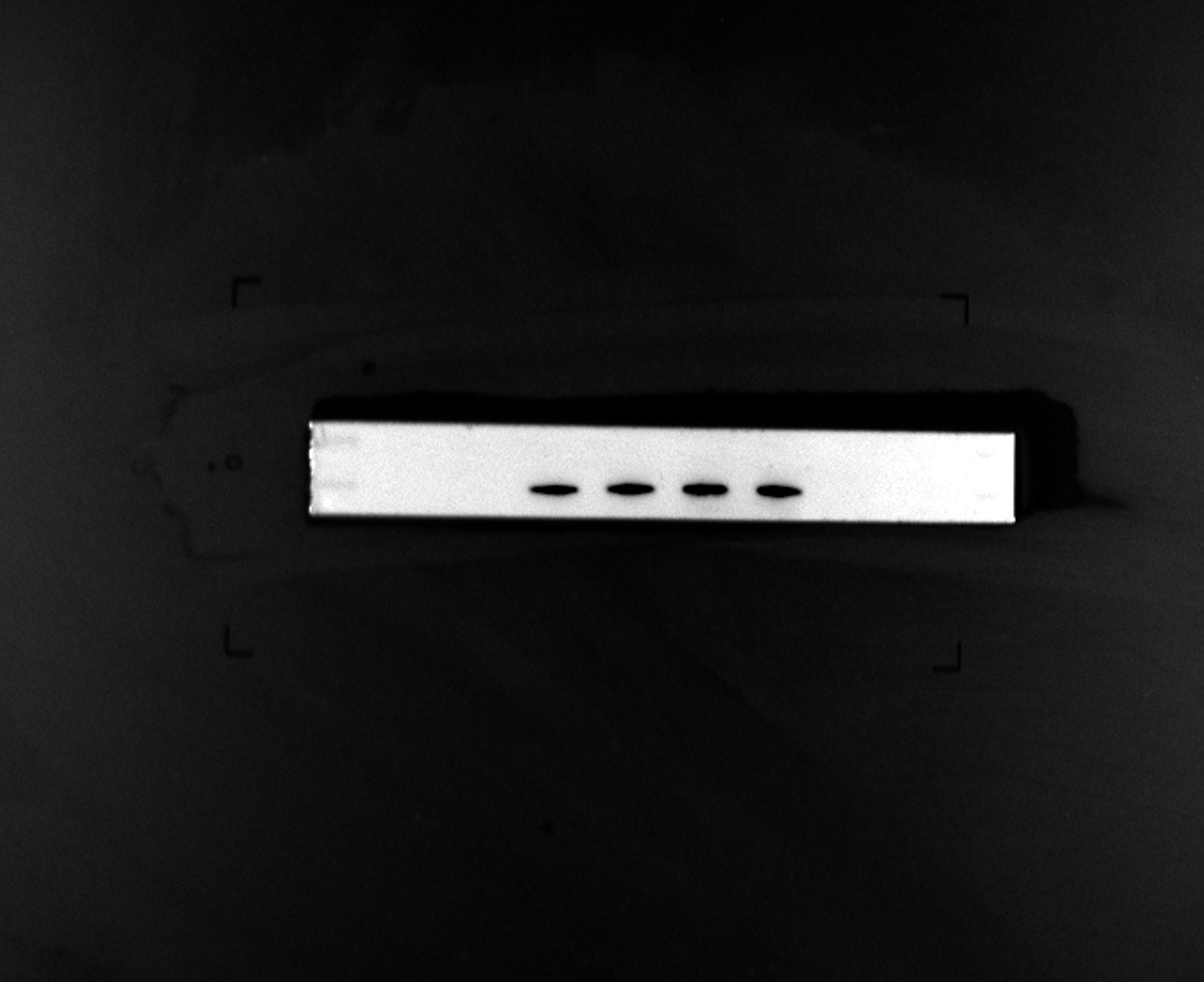


GAPDH-4 redone


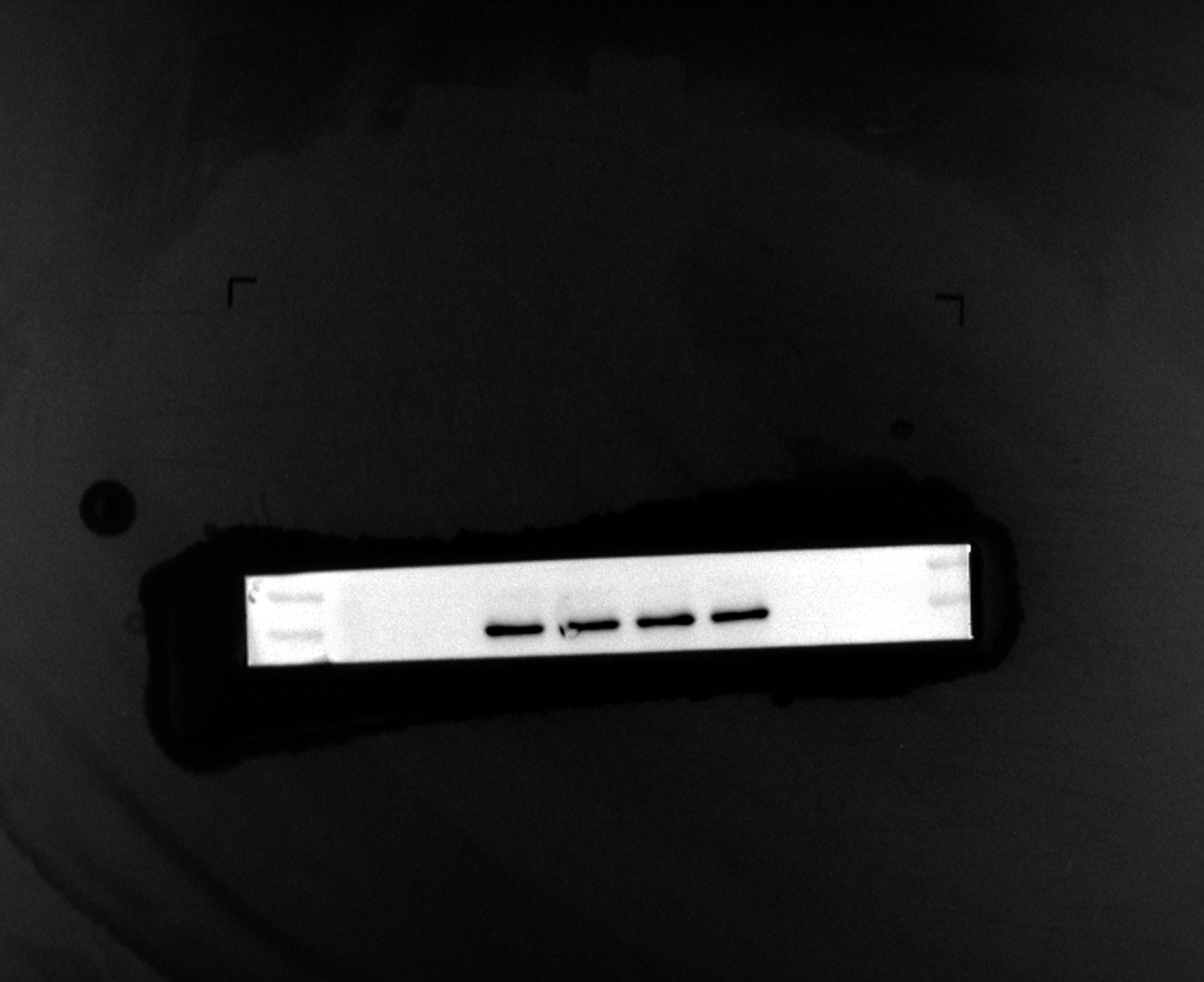


GAPDH-5 redone

1.
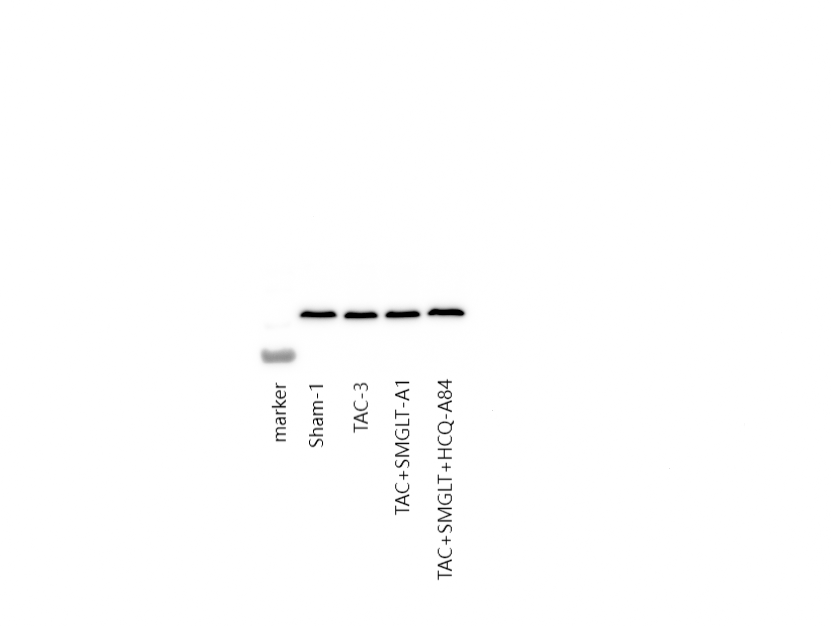
Images of blots with high contrast

GAPDH-1

GAPDH-2
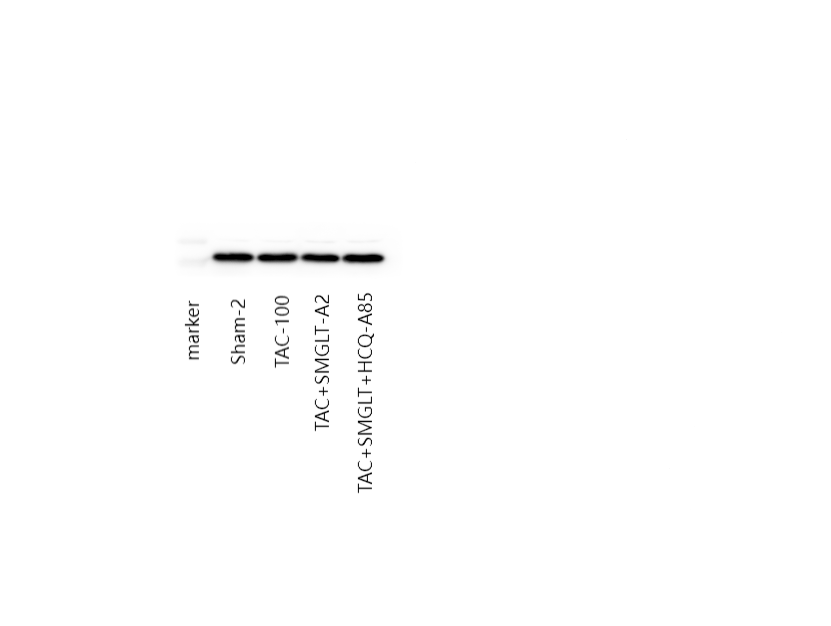


GAPDH-3
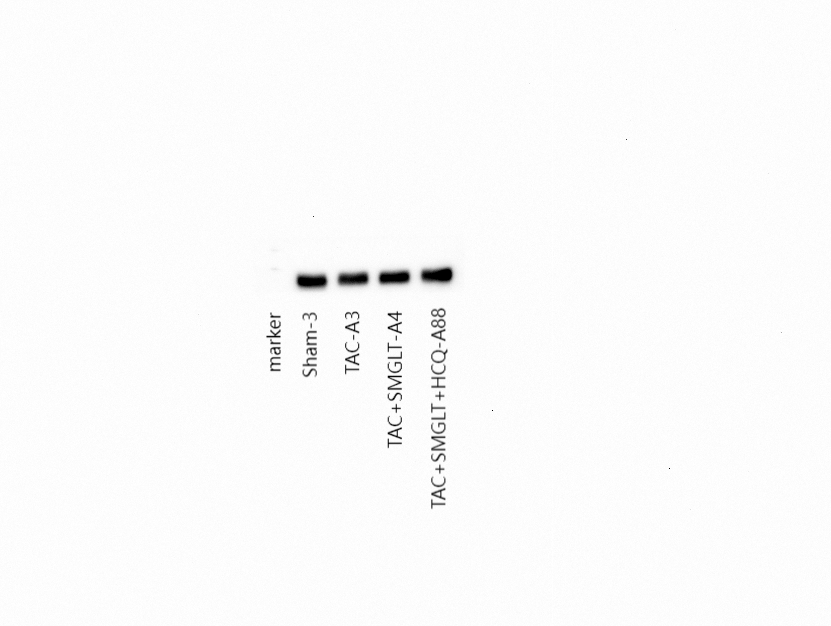


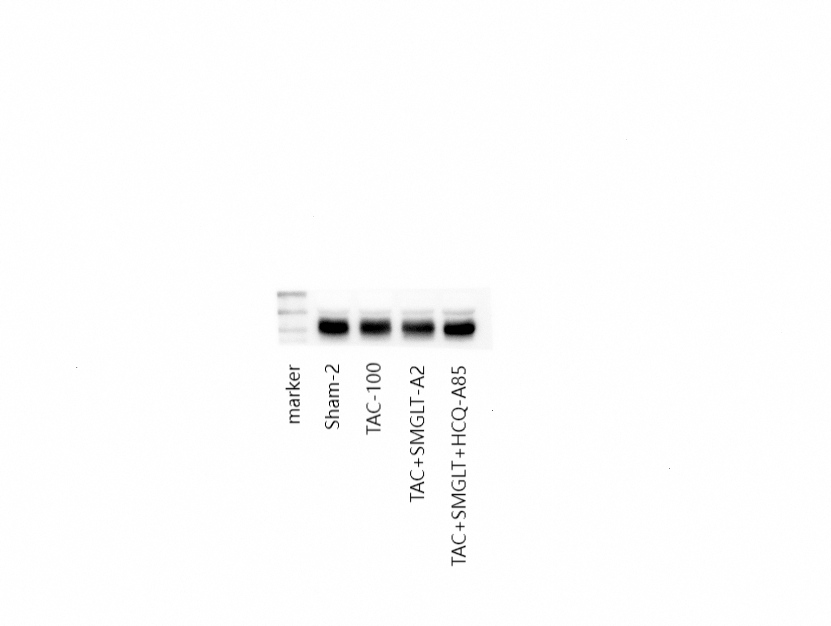


GAPDH-4


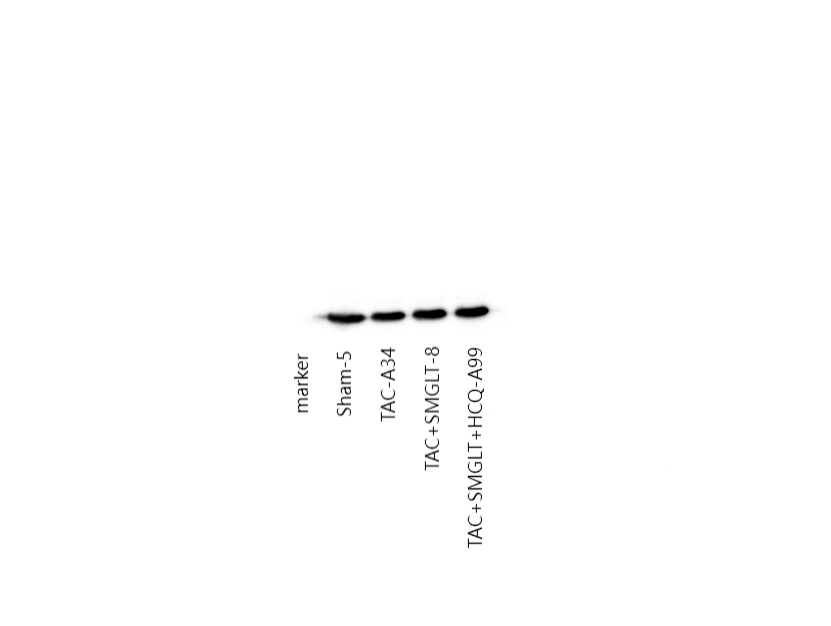


shown in figure 3D of manuscript GAPDH-5


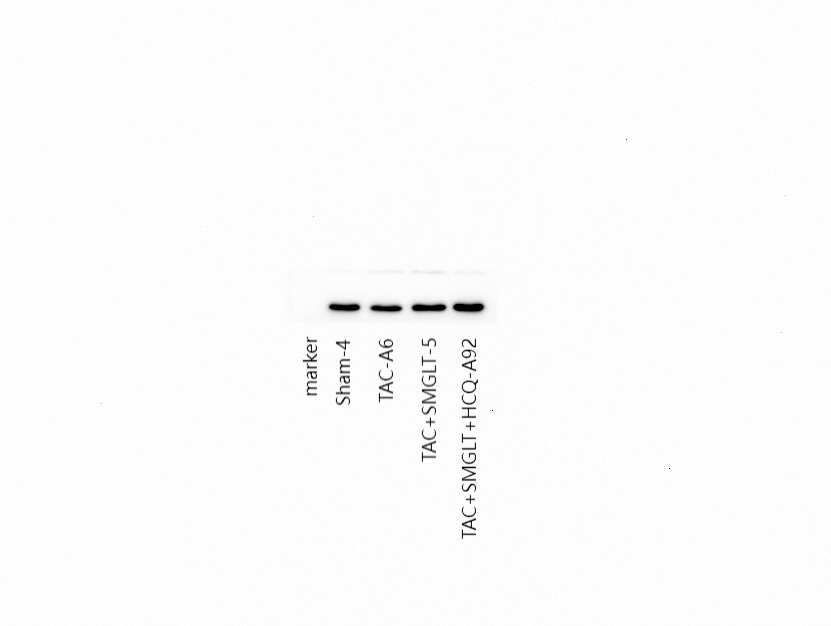


GAPDH-6
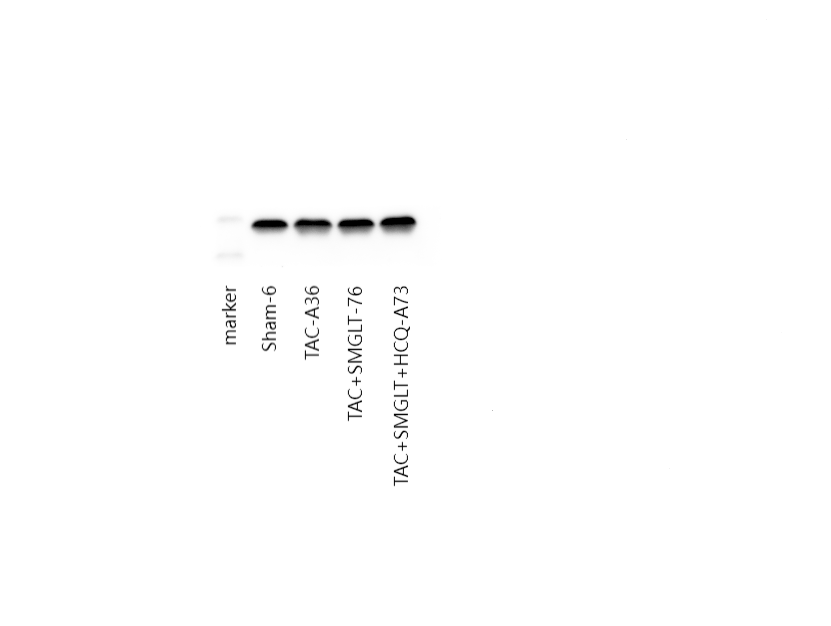


GAPDH-7
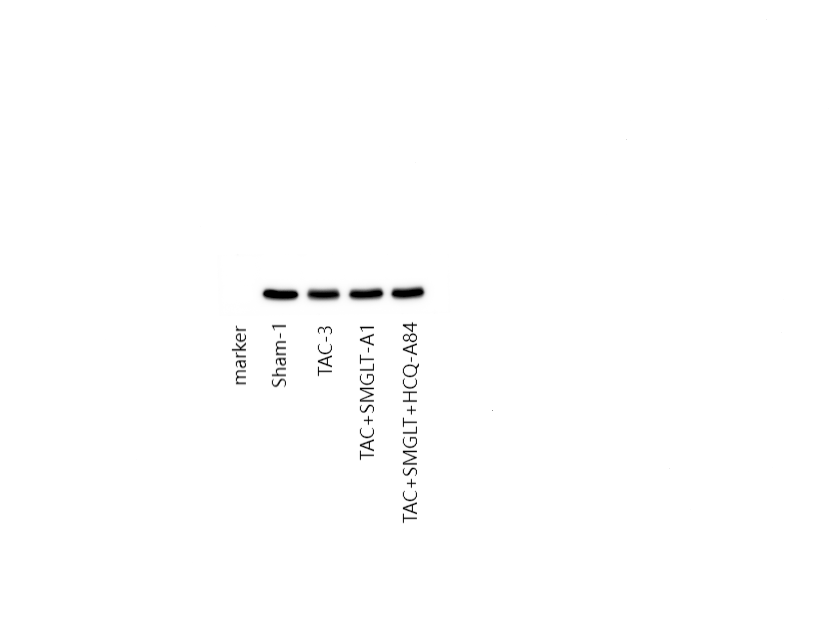


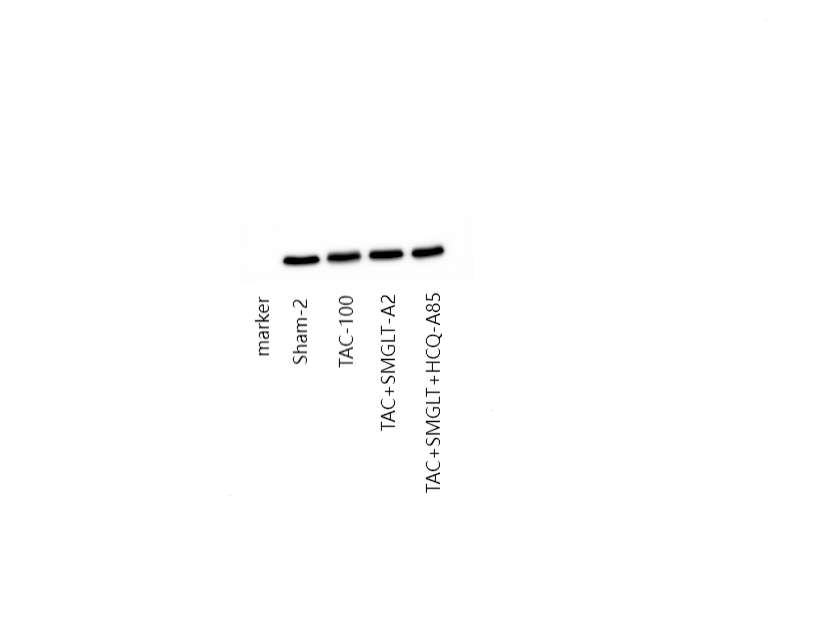


GAPDH-8

GAPDH-9
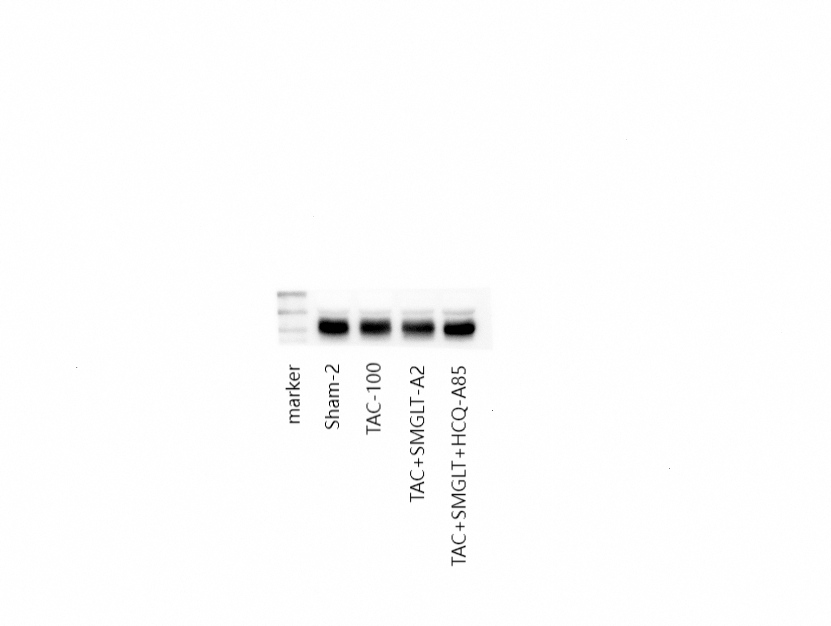


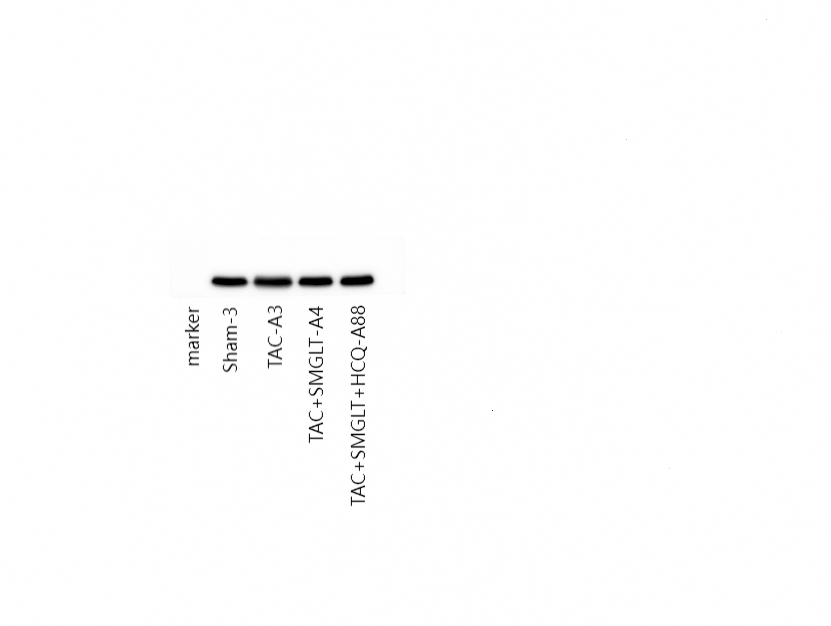


GAPDH-10


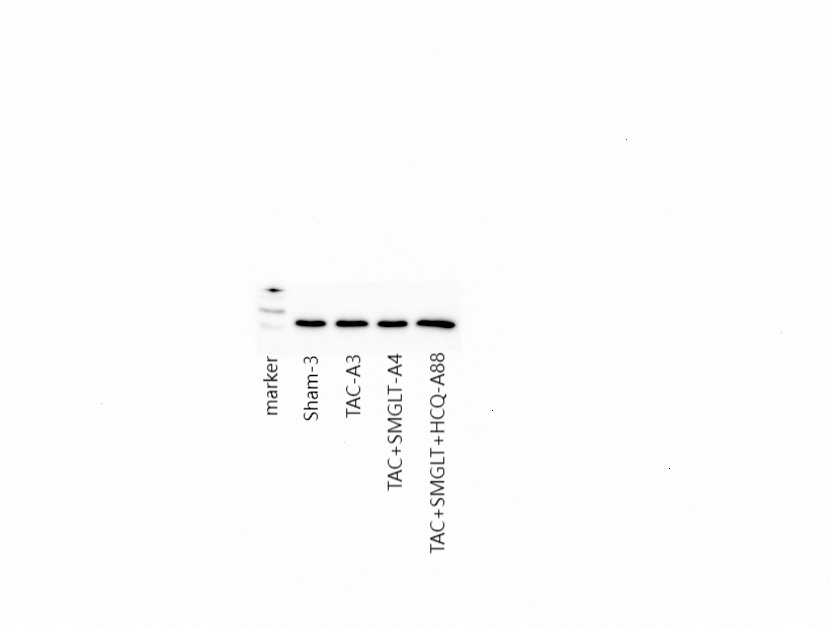
GAPDH-11

GAPDH-12
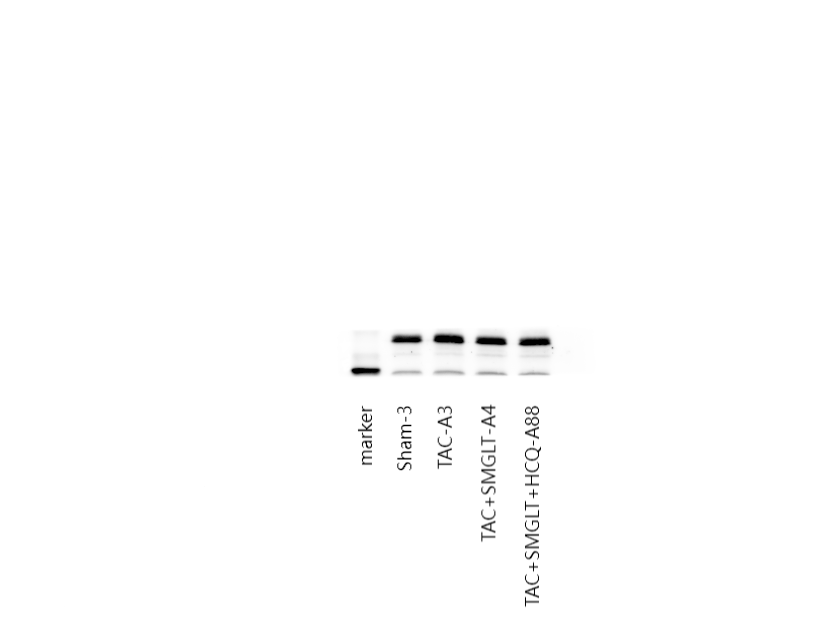


GAPDH-13
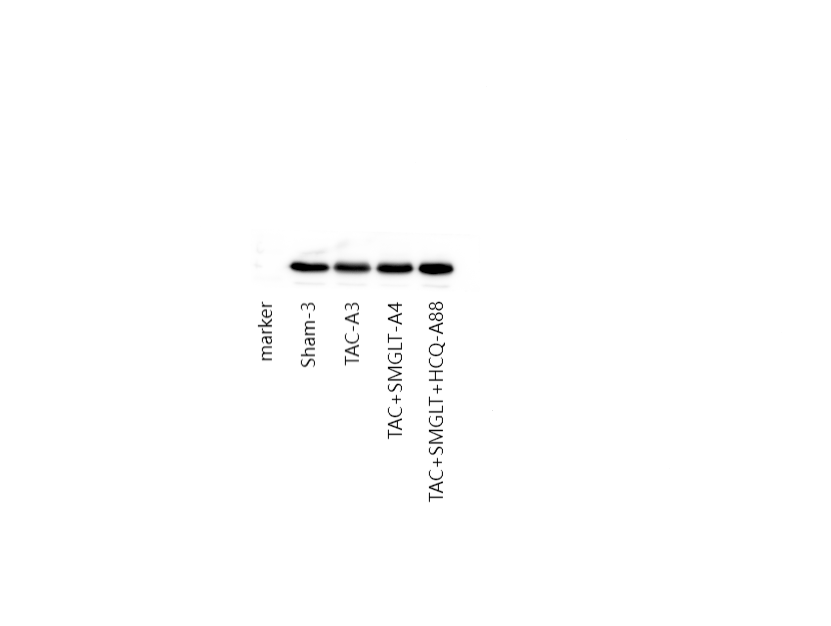


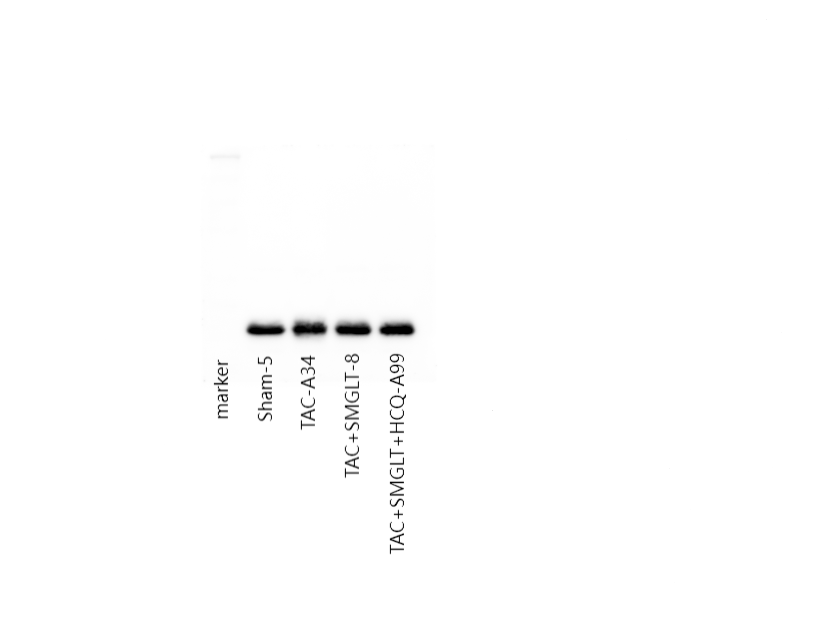
GAPDH-14

GAPDH-15
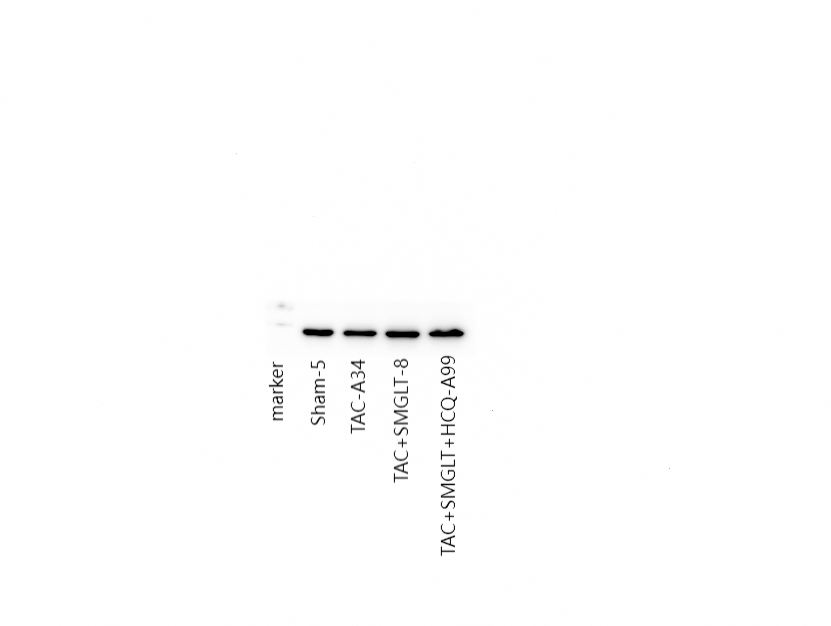


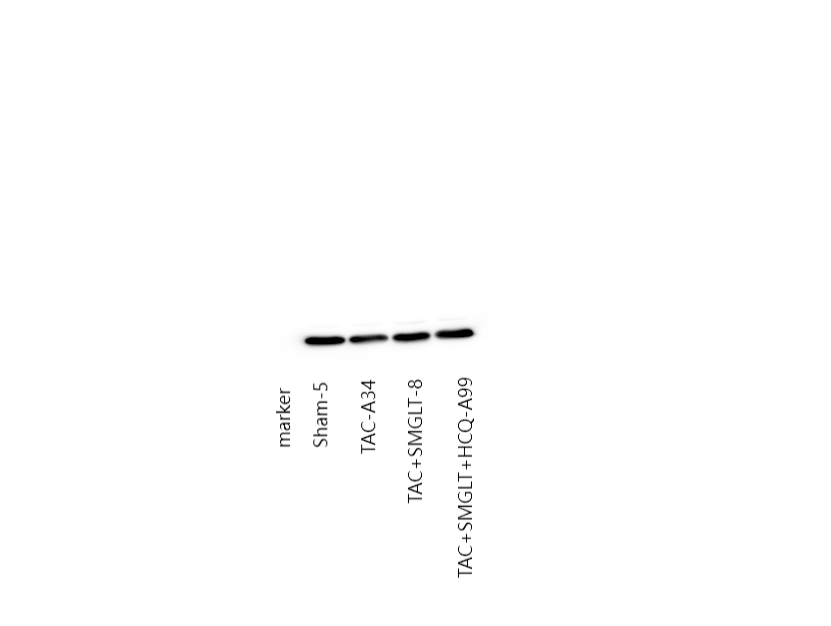


GAPDH-16

GAPDH-17
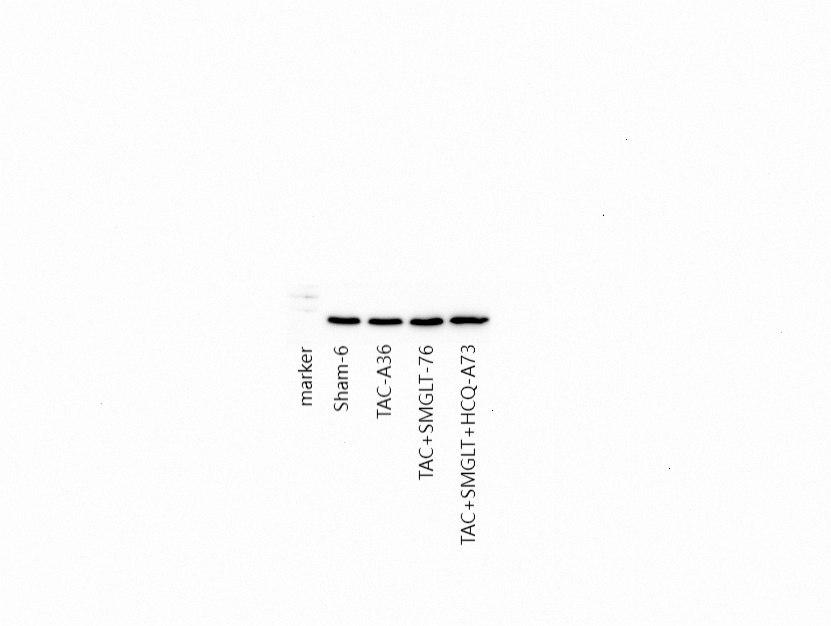


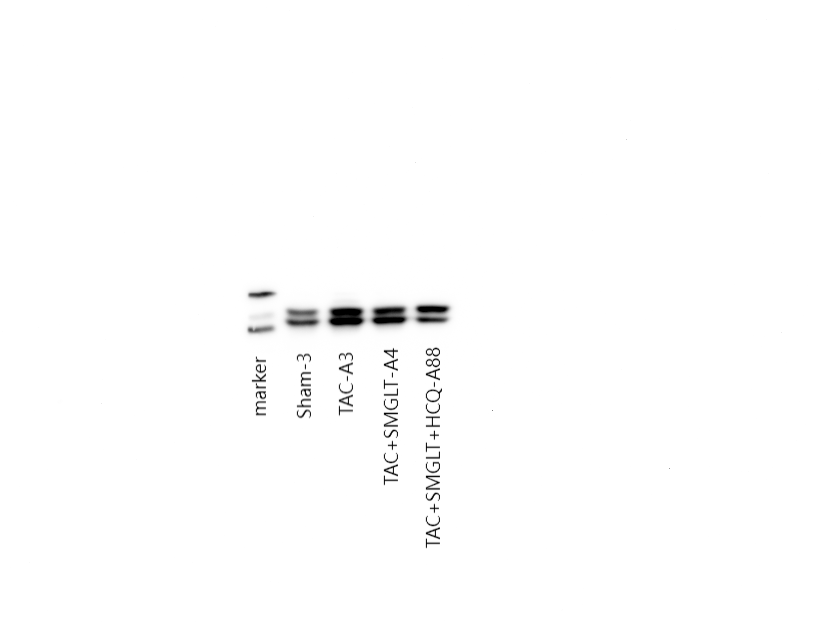


LC3B-3

LC3B-5
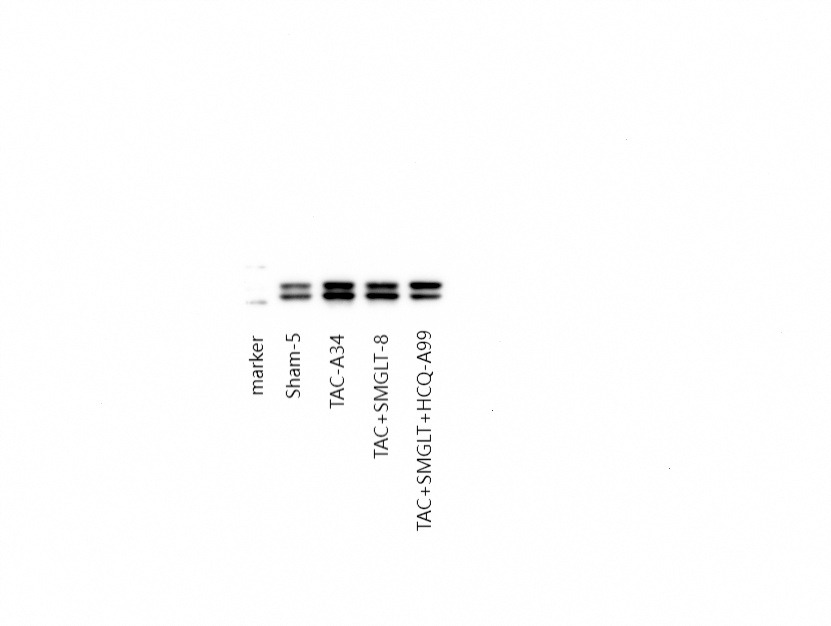


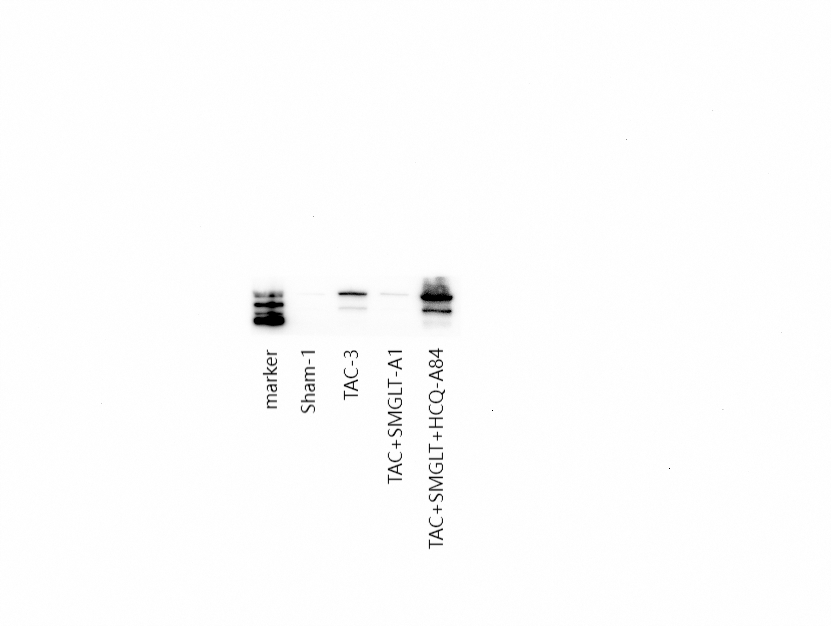


MYH7-1

MYH7-4
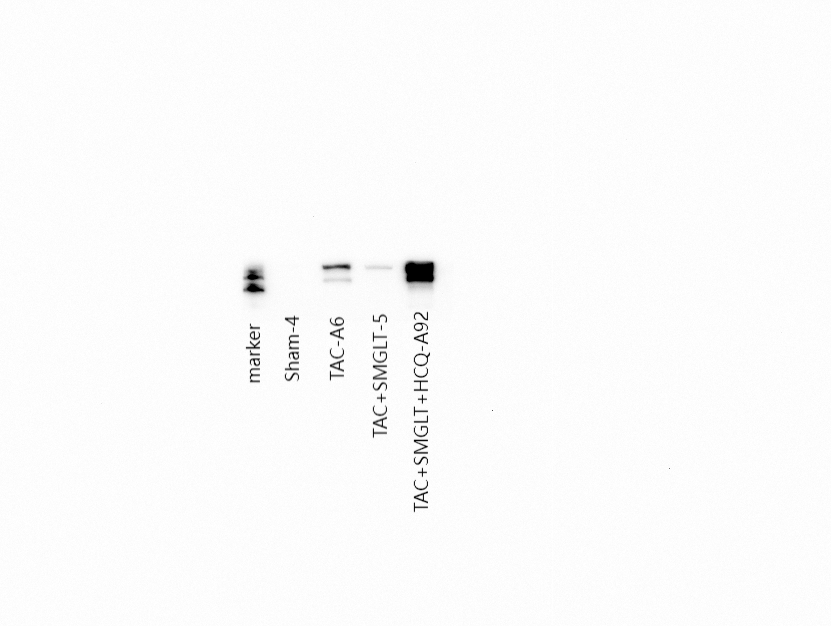


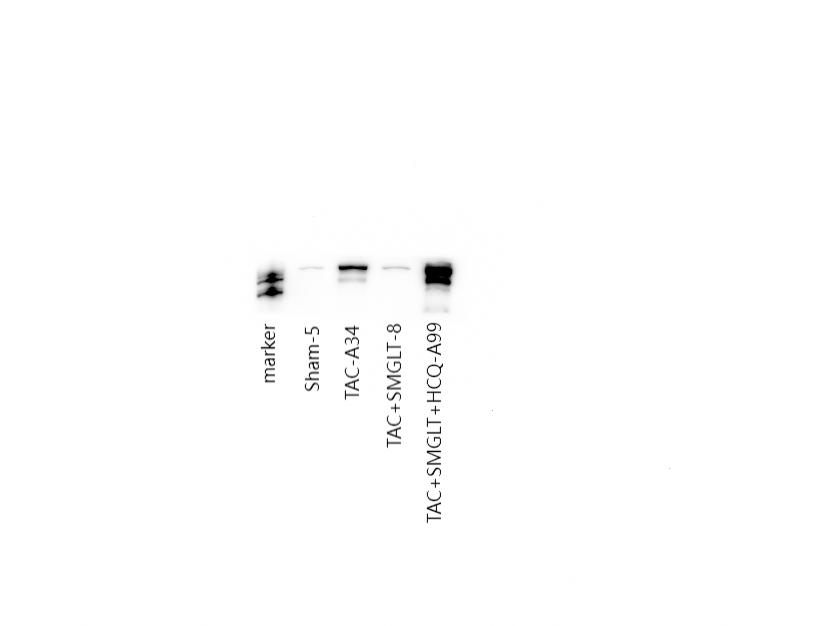


MYH7-5

MYH7-6
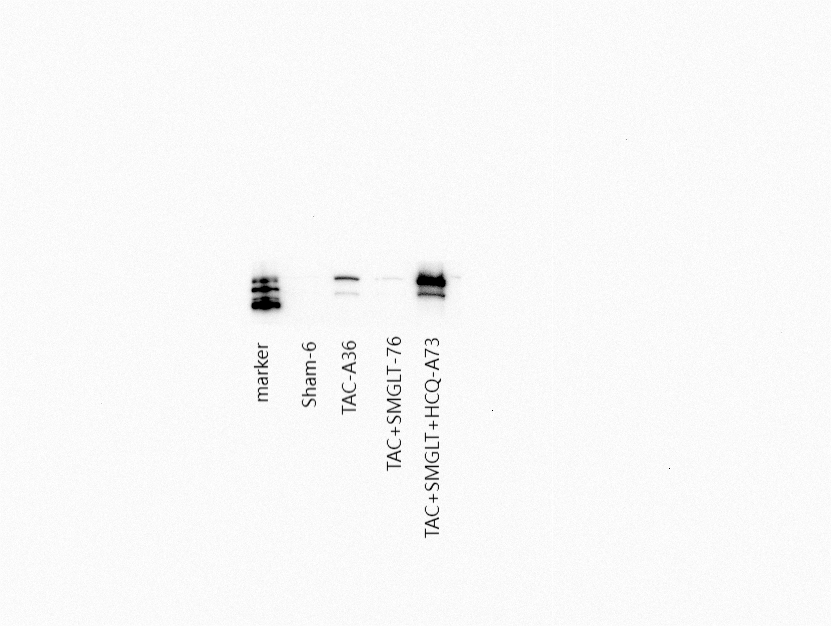


1. Blots of GAPDH with high contrast verse blots of GAPDH redone


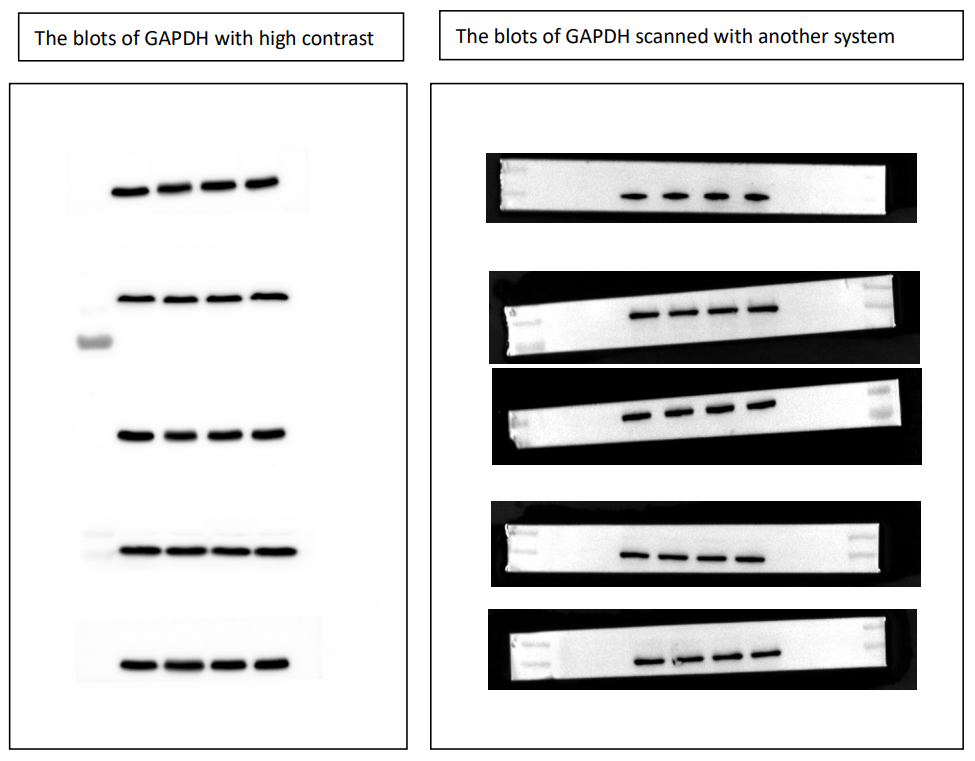

Supplement: Supplementary file 30 — Supplementary Information 30. [file 41598_2024_62465_MOESM30_ESM.docx]
