# Supplementary material for: Semaglutide ameliorates pressure overload-induced cardiac hypertrophy by improving cardiac mitophagy to suppress the activation of NLRP3 inflammasome
Source: Sci Rep. 2024 May 23;14:11824. doi: 10.1038/s41598-024-62465-6 (PMC11116553; doi:10.1038/s41598-024-62465-6)
Supplement: Supplementary file 34 — Supplementary Information 34. [file 41598_2024_62465_MOESM34_ESM.docx]

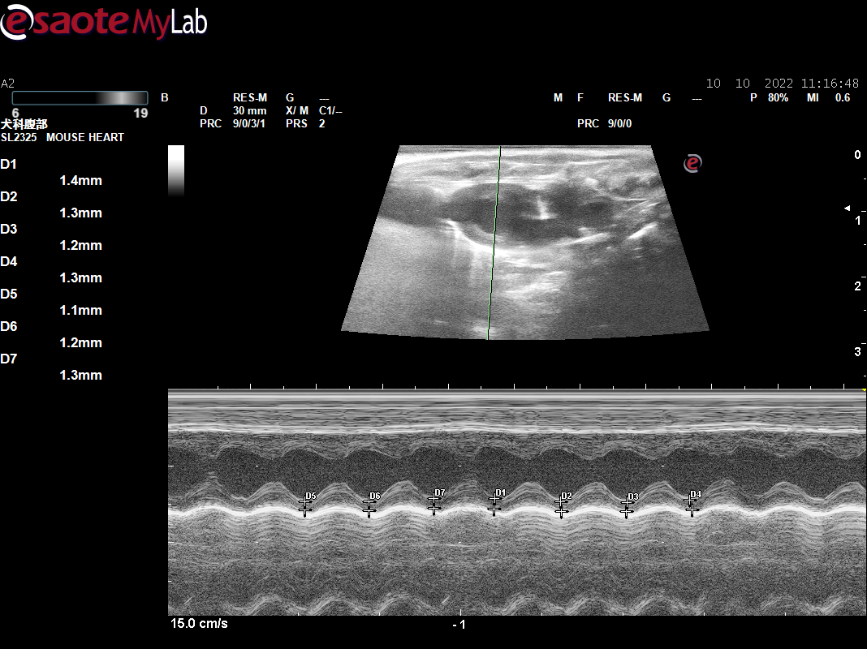

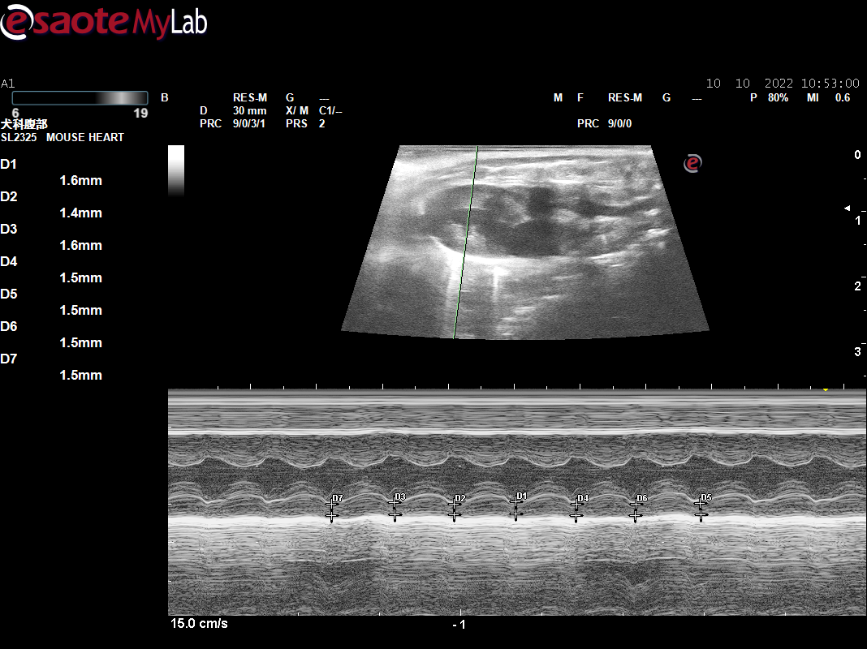

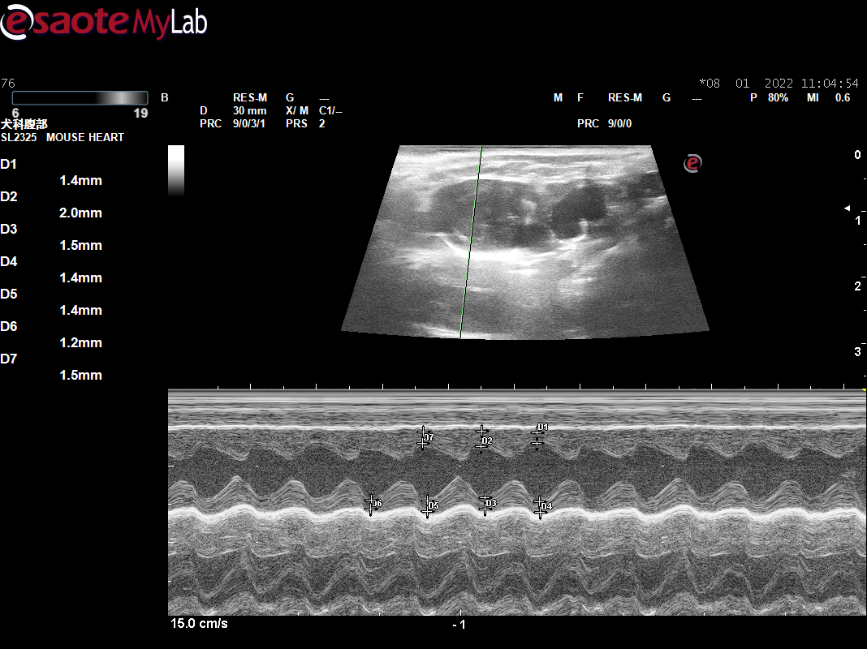

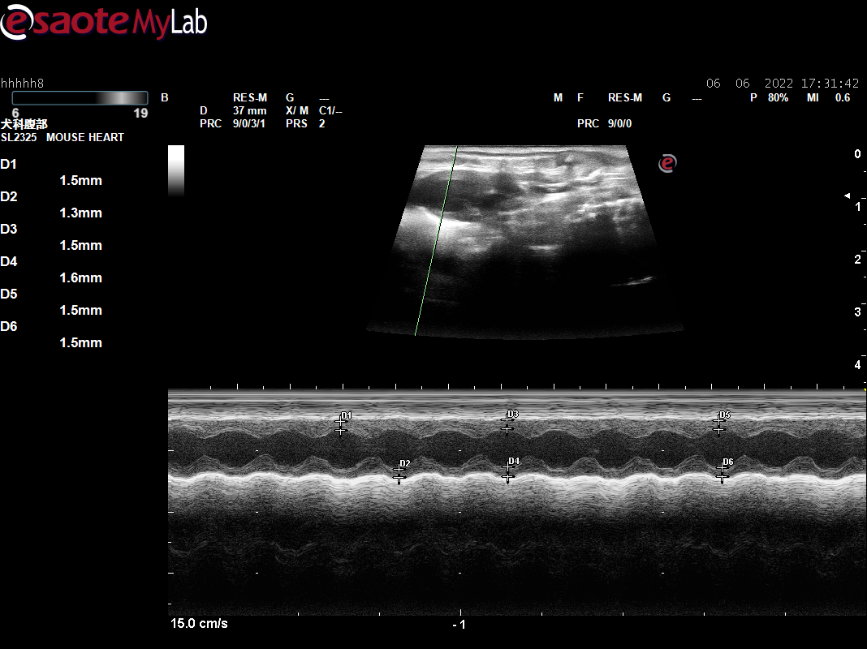

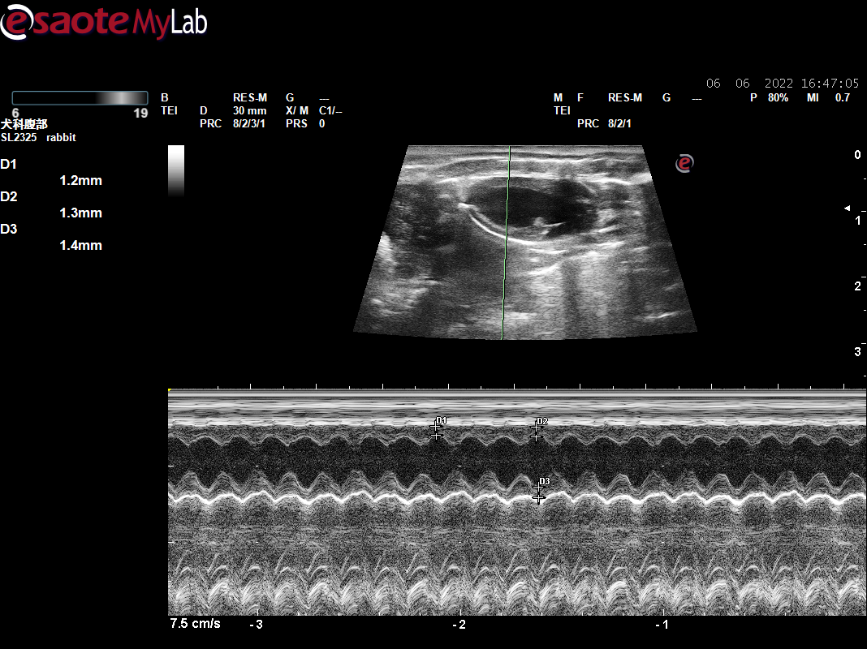

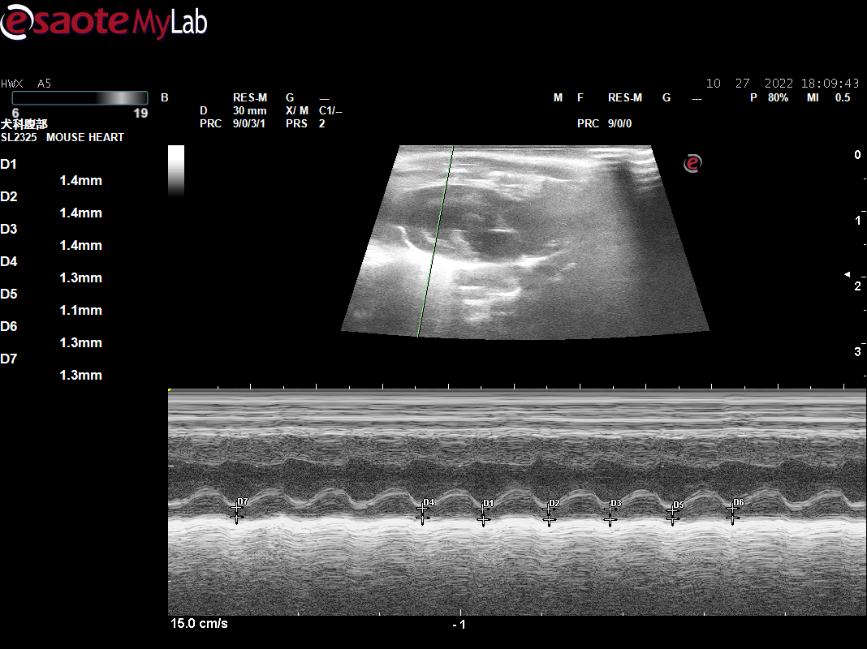


The red part of this picture is shown in our manuscript (Fig. 2E).

TAC+Semaglutide

The red part of this picture is shown in our manuscript (Fig. 1A).
