# Supplementary material for: Semaglutide ameliorates pressure overload-induced cardiac hypertrophy by improving cardiac mitophagy to suppress the activation of NLRP3 inflammasome
Source: Sci Rep. 2024 May 23;14:11824. doi: 10.1038/s41598-024-62465-6 (PMC11116553; doi:10.1038/s41598-024-62465-6)
Supplement: Supplementary file 40 — Supplementary Table 1. [file 41598_2024_62465_MOESM40_ESM.docx]

| **_Groups_**  **_Paramters_** | **Sham** | **TAC** | **TAC+Semaglutide** | **TAC+Semaglutide+HCQ** |
| --- | --- | --- | --- | --- |
| **LVPWT(mm)** | 1.39±0.03 | 2.09±0.10^**^ | 1.35±0.05^##^ | 2.19±0.04^&&&^ |
| **IVST(mm)** | 1.43±0.03 | 2.06±0.11^*^ | 1.55±0.05^#^ | 2.03±0.03^&&&^ |
| **LVMI(mm)** | 1.21±0.12 | 2.32±0.07^****^ | 1.18±0.09^####^ | 2.38±0.07^&&&&^ |
| **LVEF(%)** | 86.16±13.81 | 87.84±7.99^ns^ | 85.12±8.37^ns^ | 82.45±3.97^ns^ |
| **FS(%)** | 61.00±6.27 | 58.72±4.40^ns^ | 53.91±4.96^ns^ | 52.18±4.17^ns^ |
| **LVEDD(mm)** | 5.74±0.35 | 5.34±0.16^ns^ | 5.39±0.19^ns^ | 5.49±0.23^ns^ |
| **LVESD(mm)** | 2.31±0.46 | 2.21±0.25^ns^ | 2.46±0.25^ns^ | 2.64±0.28^ns^ |
| **LVWT(HE staining)(μm)** | 1787±100.13 | 2652±128.59^***^ | 2073±24.83^##^ | 2838±136.77^&&^ |

**Supplementary Table 1 The measurements of left ventricle by echocardiography and HE staining of the rats’ whole heart in each group**

*P value<0.05 verse Sham group

**P value<0.01 verse Sham group

***P value<0.001verse Sham group

****P value<0.0001 verse Sham group

#P value<0.05 verse TAC group

##P value<0.01 verse TAC group

####P value<0.0001 verse TAC group

&&P value<0.01verse TAC+Semaglutide group

&&&P value<0.001verse TAC+Semaglutide group

&&&&P value<0.0001verse TAC+Semaglutide group

TAC: transverse aortic constriction, HCQ: hydroxychloroquine, an inhibitor of mitophagy, LVPWT: left ventricular posterior wall thickness,

IVST: inter-ventricular septum thickness, LVMI: left ventricular mass index, LVEF: left ventricular ejection fraction, FS: fractional shortening

LVEDD: left ventricular end diastolic diameter, LVESD: left ventricular end systolic diameter, LVWT: left ventricular wall thickness,

HE:hematoxylin and eosin.

n=6 in each group when measuring the parameters of left ventricle by echocardiography, n=3 in each group when measuring LVWT by HE

staining of the whole heart
