# Supplementary material for: Semaglutide ameliorates pressure overload-induced cardiac hypertrophy by improving cardiac mitophagy to suppress the activation of NLRP3 inflammasome
Source: Sci Rep. 2024 May 23;14:11824. doi: 10.1038/s41598-024-62465-6 (PMC11116553; doi:10.1038/s41598-024-62465-6)
Supplement: Supplementary file 41 — Supplementary Table 2. [file 41598_2024_62465_MOESM41_ESM.docx]

| **_Groups_**  **_Paramters_** | **Sham**  **(n=6)** | **TAC**  **(n=6)** | **TAC+Semaglutide**  **(n=6)** | **TAC+Semaglutide+HCQ**  **(n=6)** |
| --- | --- | --- | --- | --- |
| **MYH7** | 0.043±0.02 | 0.580±0.10^*^ | 0.083±0.02^#^ | 0.471±0.11^&&&^ |
| **ANP** | 0.633±0.16 | 1.432±0.19^**^ | 0.567±0.12^##^ | 1.202±0.10^&&^ |
| **NLRP3** | 0.480±0.06 | 0.889±0.10^**^ | 0.463±0.07^##^ | 0.898±0.10^&&^ |
| **Caspase-1** | 0.690±0.04 | 1.167±0.10^***^ | 0.65±0.07^####^ | 1.025±0.07^&&^ |
| **IL-18** | 0.752±0.07 | 1.365±0.09^****^ | 0.690±0.07^####^ | 1.201±0.03^&&&&^ |
| **COXII** | 0.48±0.05 | 1.03±0.10^****^ | 0.51±0.04^####^ | 1.06±0.06^&&&&^ |
| **LC3II/LC3Ⅰ** | 1.67±0.11 | 1.14±0.06^**^ | 1.81±0.16^##^ | 1.04±0.15^&&&^ |

**Supplementary Table 2 The relative expression of proteins from left ventricular tissues of rats in each group by western-blot**

*P value<0.05 verse Sham group

**P value<0.01 verse Sham group

***P value<0.001verse Sham group

****P value<0.0001 verse Sham group

#P value<0.05 verse TAC group

##P value<0.01 verse TAC group

###P value<0.001 verse TAC group

####P value<0.0001 verse TAC group

&P value<0.05 verse TAC+Semaglutide group

&&P value<0.01verse TAC+Semaglutide group

&&&P value<0.001verse TAC+Semaglutide group

&&&&P value<0.0001verse TAC+Semaglutide group

TAC: transverse aortic constriction, HCQ: hydroxychloroquine, an inhibitor of mitophagy, MYH7: myosin heavy chain 7, ANP: atrial natriuretic peptide, IL-18: interleukin-18, COXII: cytochrome c oxidase subunit II
