# Supplementary material for: Semaglutide ameliorates pressure overload-induced cardiac hypertrophy by improving cardiac mitophagy to suppress the activation of NLRP3 inflammasome
Source: Sci Rep. 2024 May 23;14:11824. doi: 10.1038/s41598-024-62465-6 (PMC11116553; doi:10.1038/s41598-024-62465-6)
Supplement: Supplementary file 42 — Supplementary Table 3. [file 41598_2024_62465_MOESM42_ESM.docx]

**^Supplementary Table 3 The quantity measurements of mitochondria and autophagosomes from left ventricular tissues of rats in each group^**

| **^Groups^**  **_Paramters_** | **Sham**  **(n=6)** | **TAC**  **(n=6)** | **TAC+Semaglutide**  **(n=6)** | **TAC+Semaglutide+HCQ**  **(n=6)** |
| --- | --- | --- | --- | --- |
| **Mitochondria** | 18.50±1.75 | 31.00±1.29^****^ | 18.00±1.27^####^ | 29.50±2.31^&&&^ |
| **Autophagosomes** | 2.50(2.00, 3.25) | 0.00(0.00, 0.25)^*^ | 2.50(2.00, 3.25) ^#^ | 0.00(0.00, 0.00) ^&&^ |

*P value<0.05 verse Sham group

****P value<0.0001 verse Sham group

#P value<0.05 verse TAC group

####P value<0.0001 verse TAC group

&&P value<0.01verse TAC+Semaglutide group

&&&P value<0.001verse TAC+Semaglutide group

TAC: transverse aortic constriction, HCQ: hydroxychloroquine, an inhibitor of mitophagy.
