# Supplementary material for: Semaglutide ameliorates pressure overload-induced cardiac hypertrophy by improving cardiac mitophagy to suppress the activation of NLRP3 inflammasome
Source: Sci Rep. 2024 May 23;14:11824. doi: 10.1038/s41598-024-62465-6 (PMC11116553; doi:10.1038/s41598-024-62465-6)
Supplement: Supplementary file 43 — Supplementary Table 4. [file 41598_2024_62465_MOESM43_ESM.docx]

**Supplementary Table 4 The optical density measurement of the proteins from left ventricular tissues of rats by immunohistochemistry in each group**

| **_Groups_**  **_Paramters_** | **Sham**  **(n=6)** | **TAC**  **(n=6)** | **TAC+Semaglutide**  **(n=6)** | **TAC+Semaglutide+HCQ**  **(n=6)** |
| --- | --- | --- | --- | --- |
| **MYH7** | 0.052±0.006 | 0.162±0.001^****^ | 0.071±0.002^####^ | 0.150±0.003^&&&&^ |
| **ANP** | 0.047±0.005 | 0.150±0.002^****^ | 0.010±0.002^####^ | 0.166±0.002^&&&&^ |
| **NLRP3** | 0.006±0.002 | 0.150±0.002^****^ | 0.016±0.002^####^ | 0.161±0.003^&&&&^ |
| **IL-18** | 0.029±0.007 | 0.153±0.003^****^ | 0.013±0.004^####^ | 0.117±0.003^&&&&^ |
| **COXII** | 0.004±0.001 | 0.140±0.001^****^ | 0.016±0.006^####^ | 0.147±0.003^&&&&^ |

****P value<0.0001 verse Sham group

####P value<0.0001 verse TAC group

&&&&P value<0.0001verse TAC+Semaglutide group

TAC: transverse aortic constriction, HCQ: hydroxychloroquine, an inhibitor of mitophagy, MYH7: myosin heavy chain 7,

ANP: atrial natriuretic peptide, IL-18: interleukin-18, COXII: cytochrome c oxidase subunit II
