# Supplementary material for: Semaglutide ameliorates pressure overload-induced cardiac hypertrophy by improving cardiac mitophagy to suppress the activation of NLRP3 inflammasome
Source: Sci Rep. 2024 May 23;14:11824. doi: 10.1038/s41598-024-62465-6 (PMC11116553; doi:10.1038/s41598-024-62465-6)
Supplement: Supplementary file 44 — Supplementary Information 40. [file 41598_2024_62465_MOESM44_ESM.docx]

TAC+SMGLT-A1

TAC+SMGLT+HCQ-A84

Sham-1

TAC-3

250KDa

150KDa

100KDa


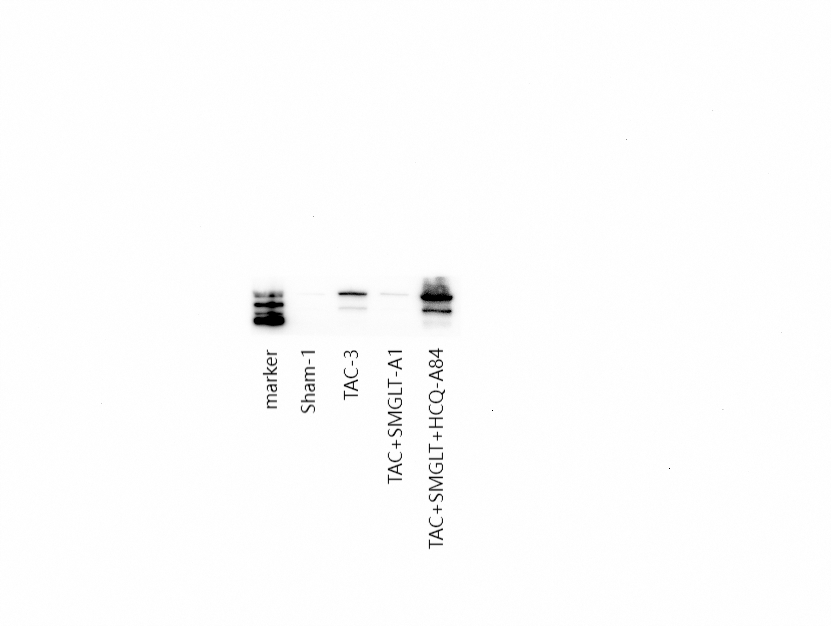


220KDa MYH7


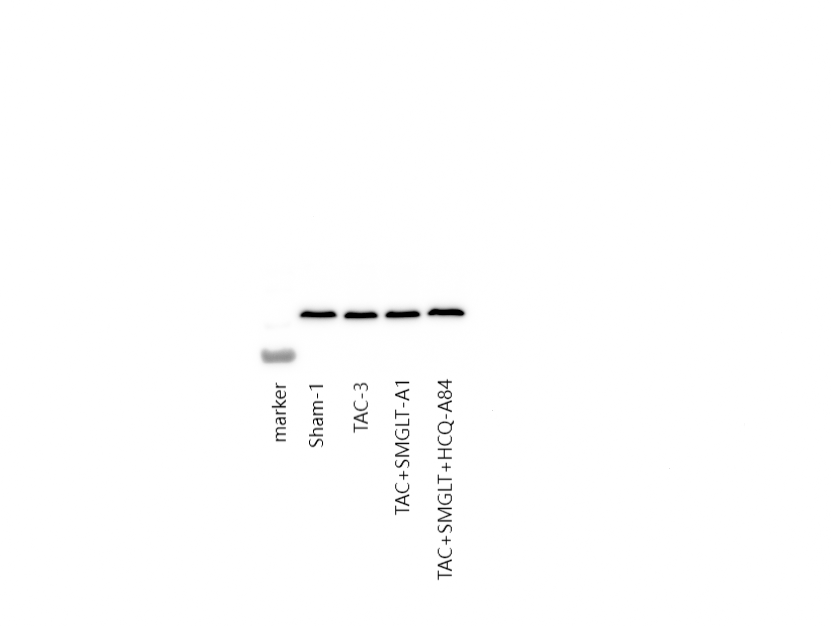


37KDa GAPDH

35KDa

25KDa

TAC+SMGLT+HCQ-A85

TAC+SMGLT-A2

TAC-100

Sham-2


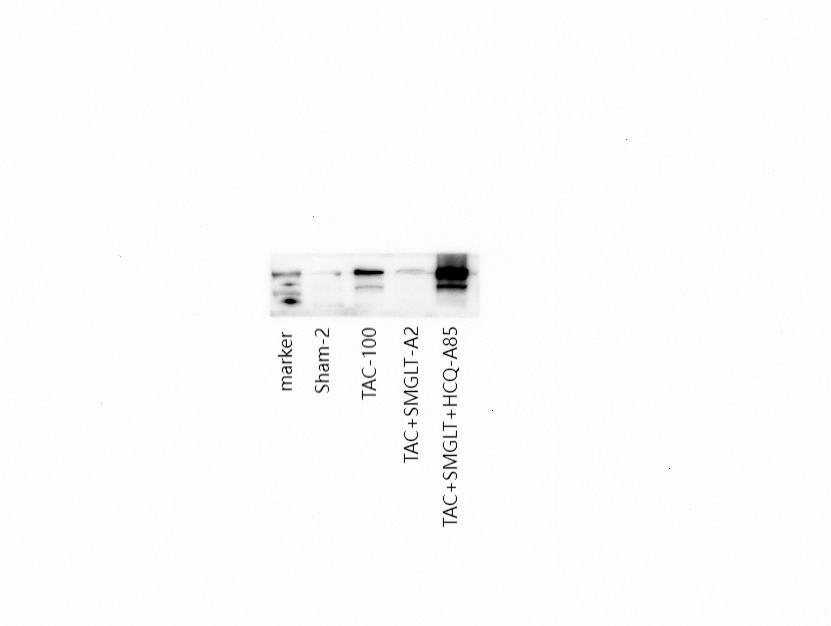


220KDa MYH7

250KDa

150KDa


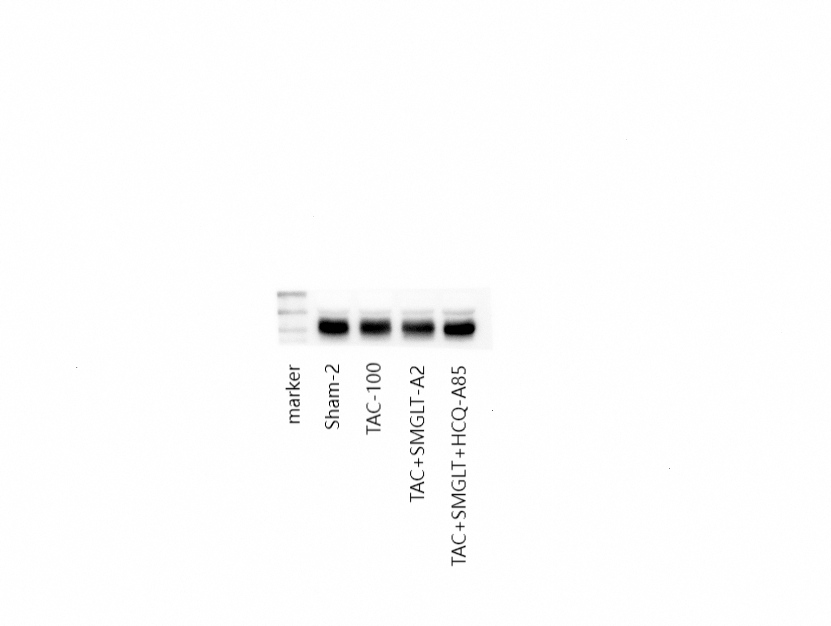


37KDa GAPDH

40KDa

35KDa

TAC+SMGLT+HCQ-A88

TAC+SMGLT-A4

TAC-A3

Sham-3


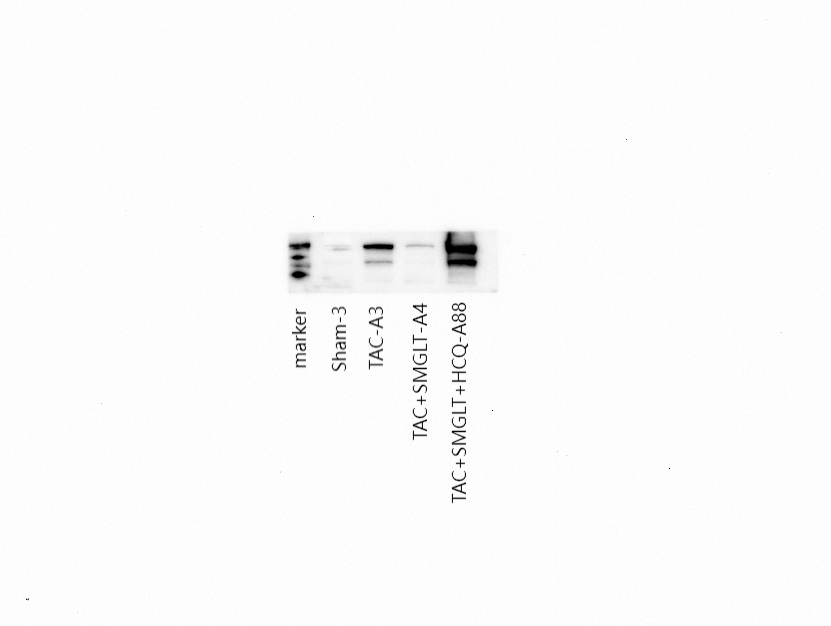


220KDa MYH7

250KDa

150KDa


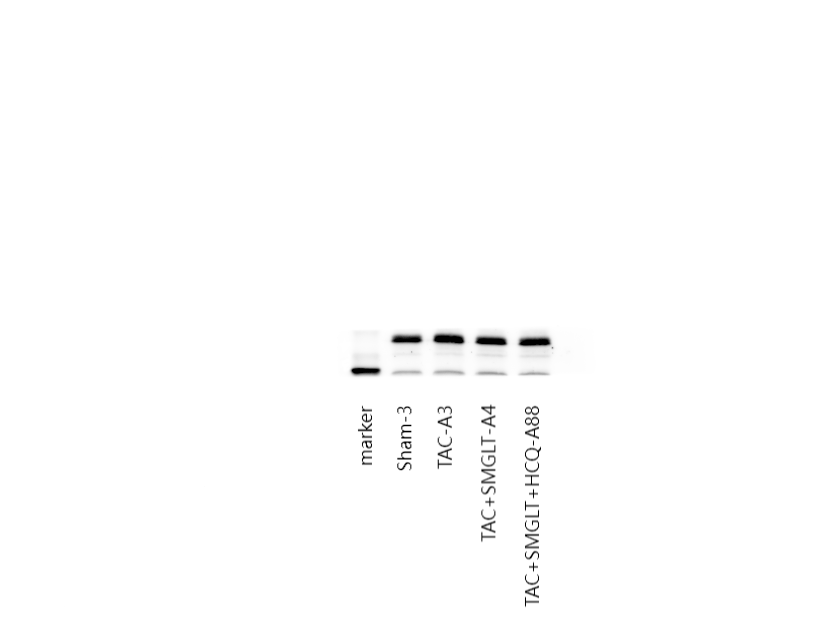


37KDa GAPDH

40KDa

35KDa

TAC+SMGLT+HCQ-A92

TAC+SMGLT-5

Sham-4

TAC-A6


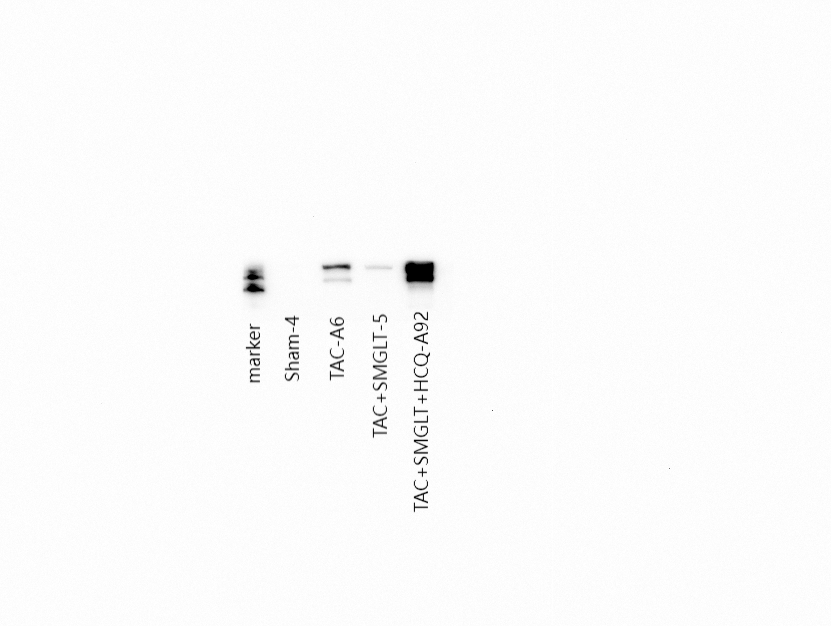


220KDa MYH7

150KDa

100KDa


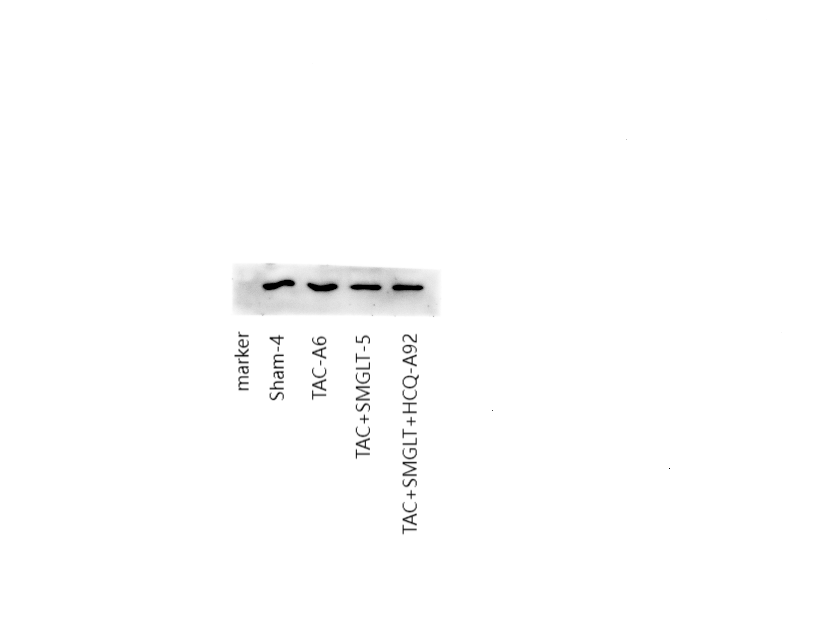


37KDa GAPDH

TAC+SMGLT+HCQ-A99

TAC+SMGLT-8

Sham-5

TAC-A34


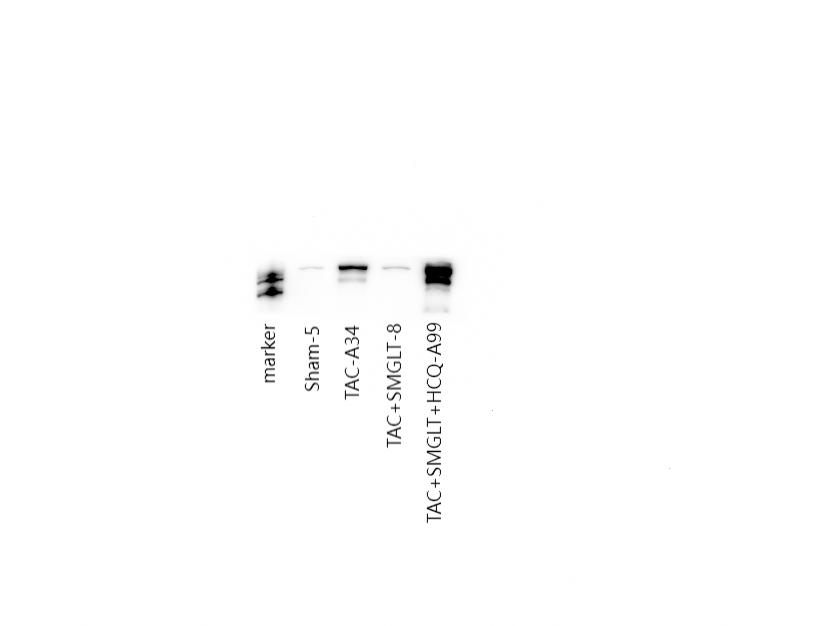


150KDa

100KDa

220KDa MYH7


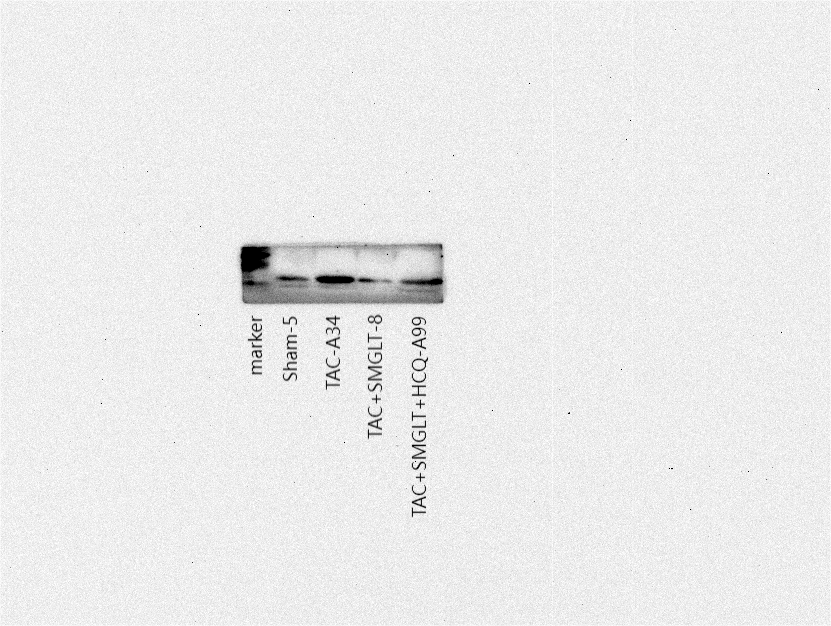


74KDa COX II

70KDa


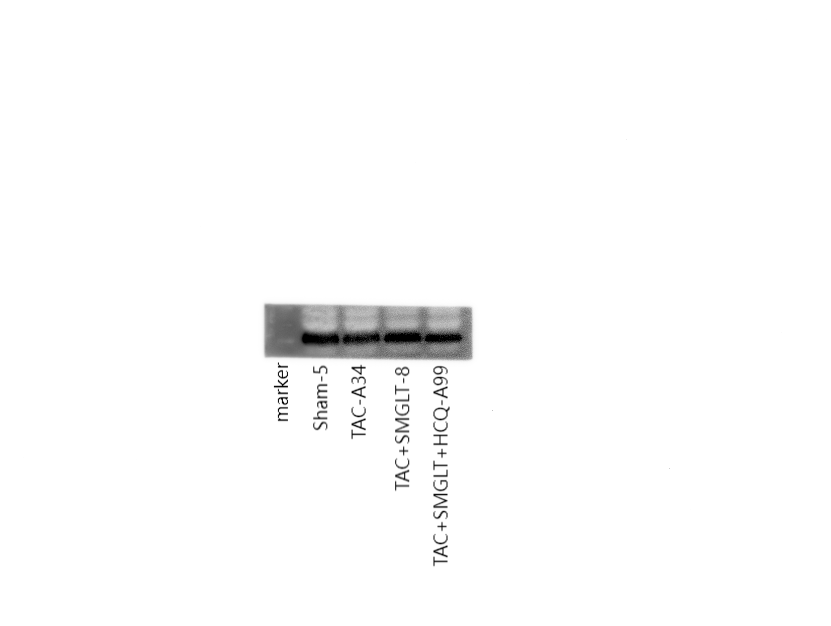


37KDa GAPDH

TAC+SMGLT+HCQ-A73

This figure of MYH7 was shown in our manuscript (Fig. 3A).


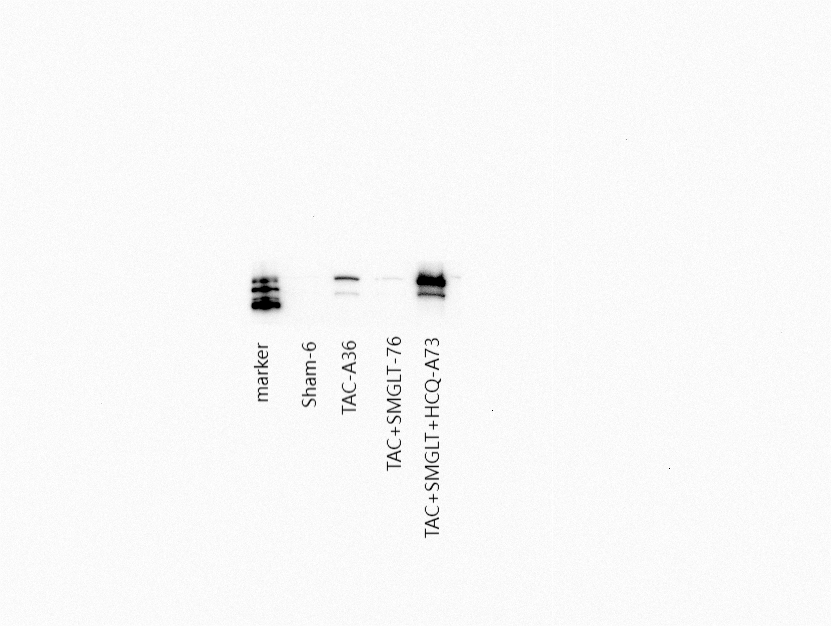


TAC+SMGLT-76

TAC-A36

Sham-6

220KDa MYH7

250KDa

150KDa

100KDa

50KDa

40KDa


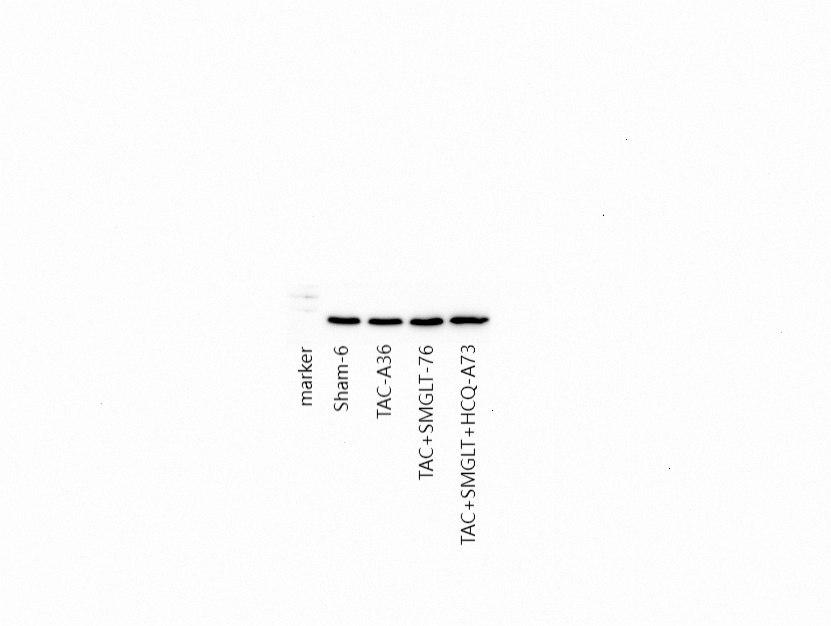


37KDa GAPDH


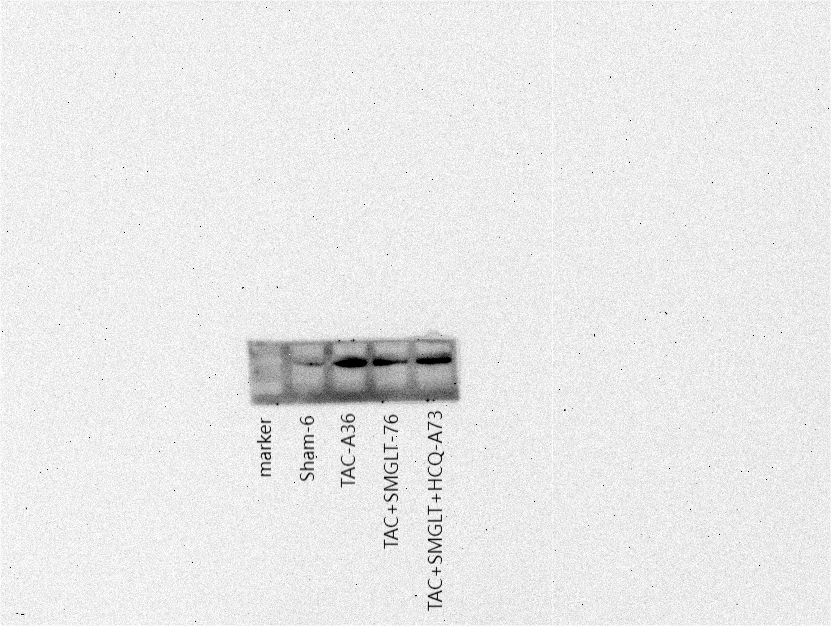


27KDa ANP

TAC+SMGLT+HCQ-A84

TAC+SMGLT-A1

Sham-1

TAC-3


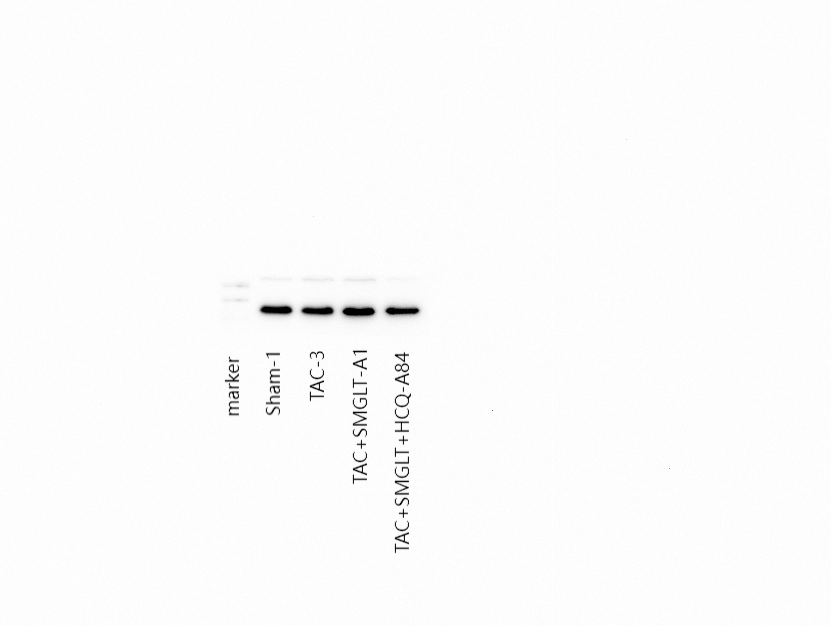


37KDa GAPDH

50KDa

40KDa


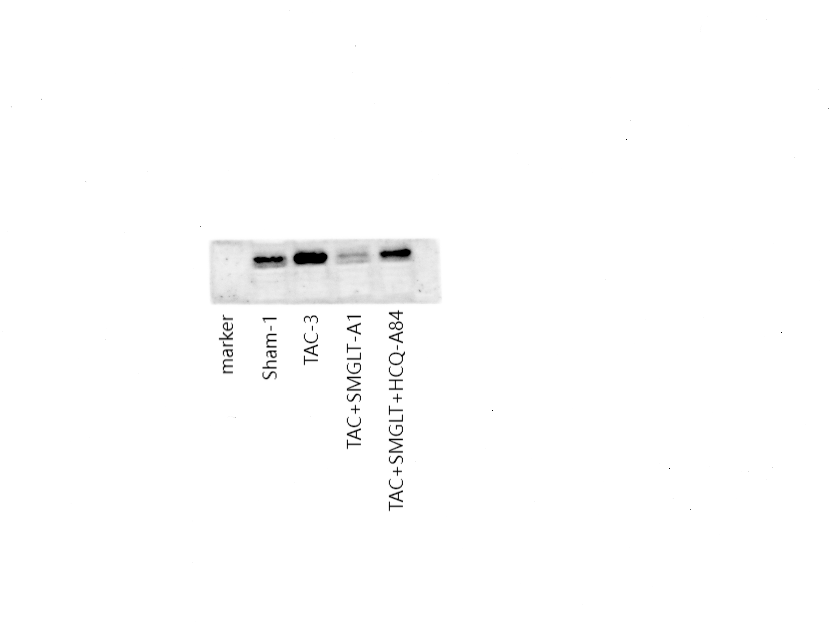


27KDa ANP

TAC+SMGLT+HCQ-85

TAC+SMGLT-A2

50KDa

40KDa

35KDa

Sham-2

TAC-100


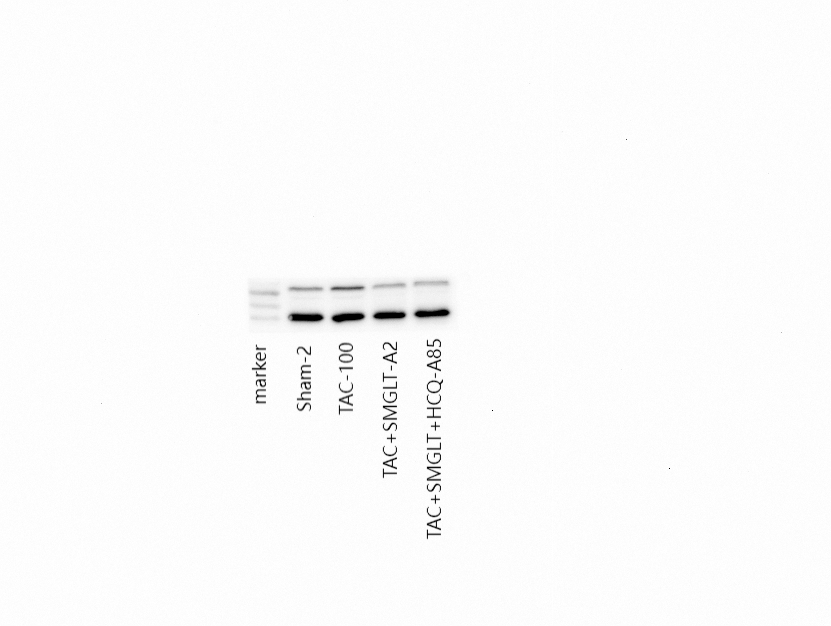

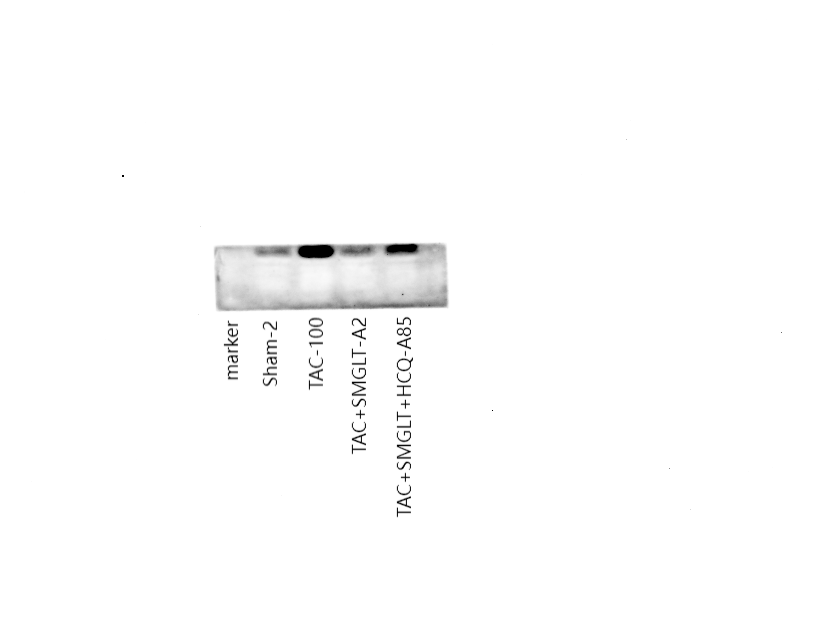


27KDa ANP

37KDa GAPDH

TAC+SMGLT+HCQ-A88

TAC+SMGLT-A4

TAC-A3

Sham-3


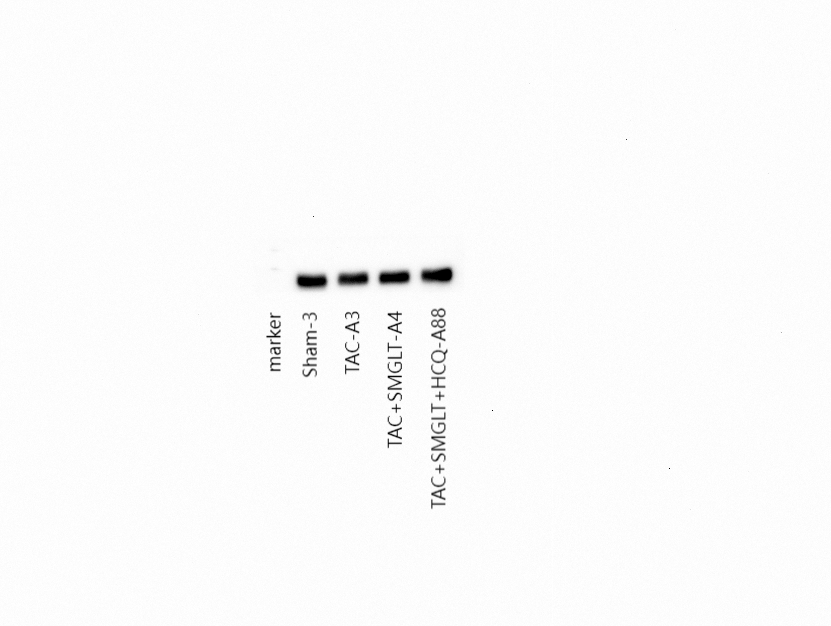


37KDa GAPDH


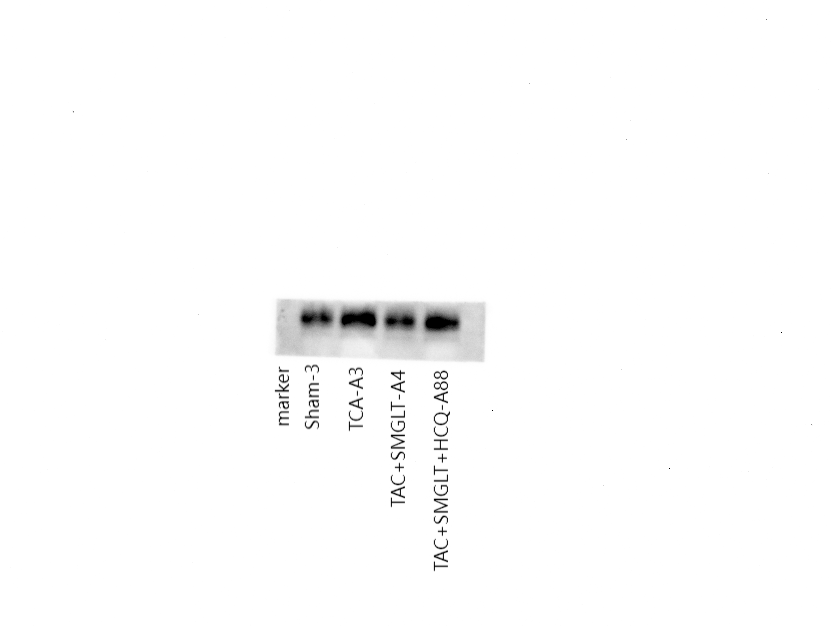


27KDa ANP

TAC+SMGLT+HCQ-A92

TAC+SMGLT-5

TAC-A6

Sham-4


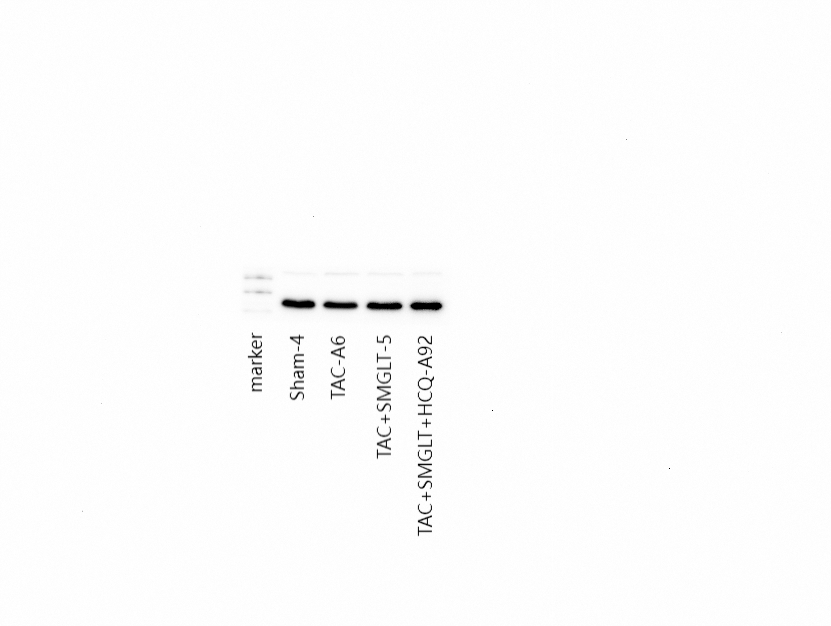


50KDa

40KDa

35KDa

37KDa GAPDH


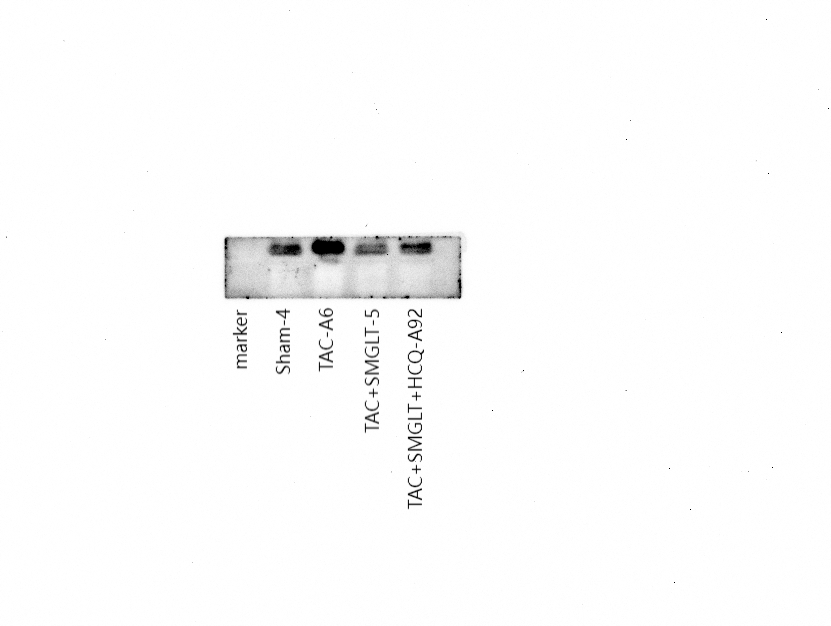


27KDa ANP

TAC+SMGLT+HCQ-A99

TAC-A34

Sham-5

Sham-5

TAC+SMGLT-8

TAC+SMGLT-8

TAC-A34

TAC+SMGLT+HCQ-A99


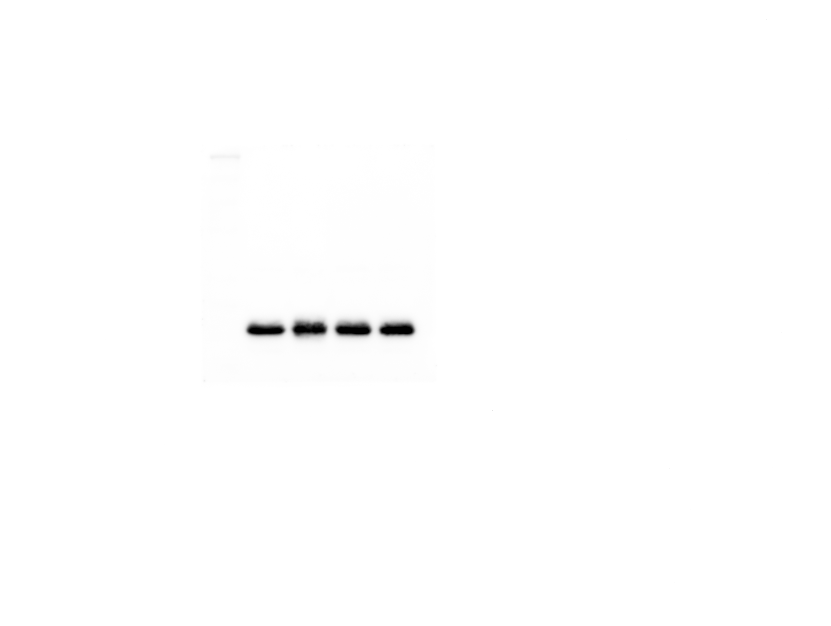

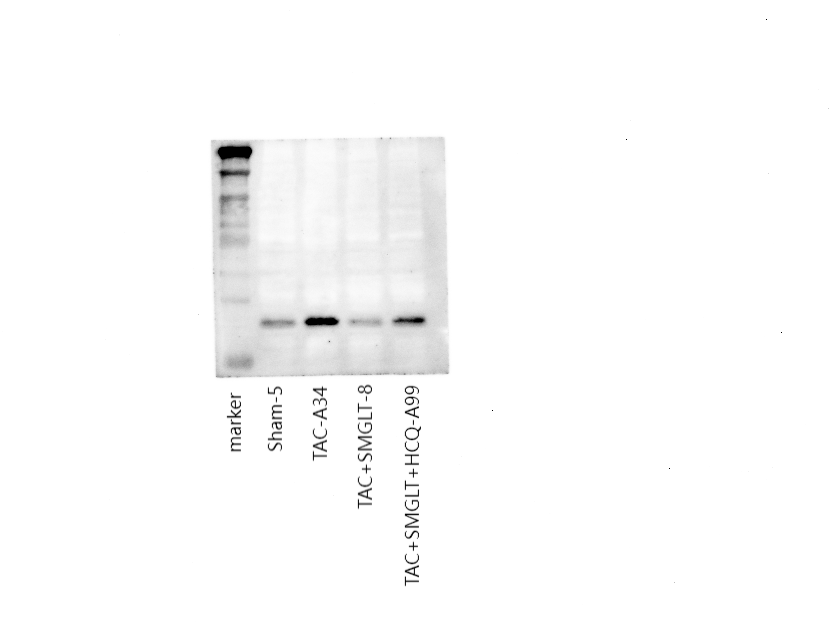


This figure of ANP was shown in our manuscript (Fig. 3A).

27KDa ANP

35KDa

25KDa

37KDa GAPDH

TAC+SMGLT+HCQ-A84

TAC+SMGLT-A1

Sham-1

TAC-3


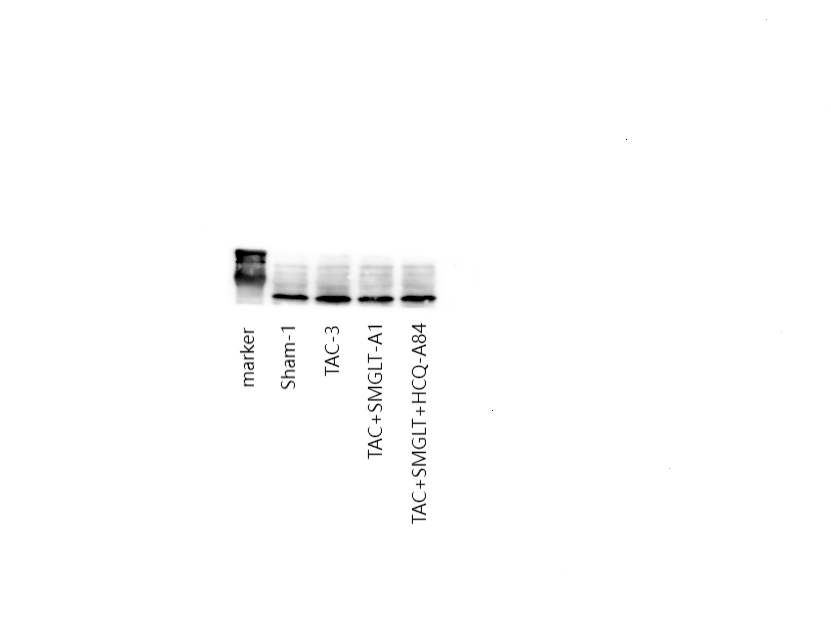


100KDa

74KDa COX II


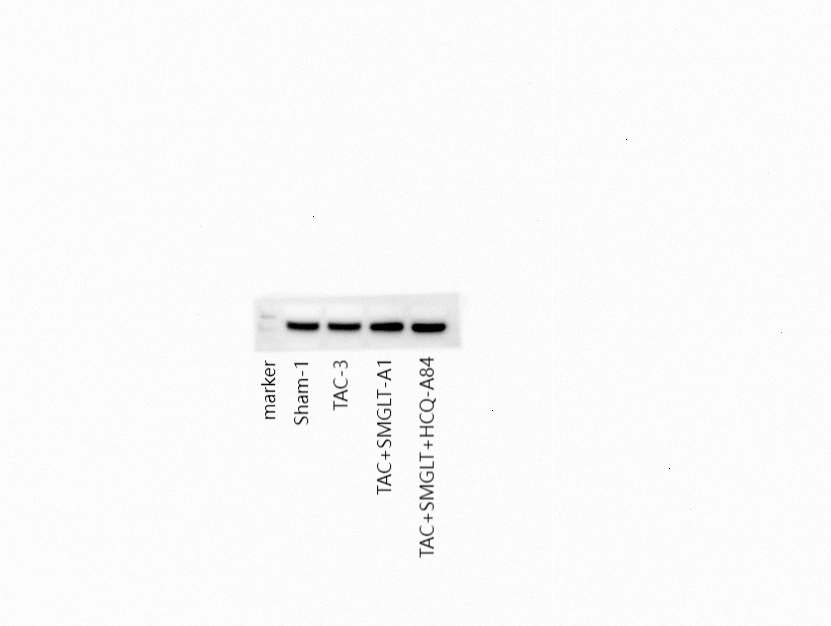


40KDa

35KDa

37KDa GAPDH

TAC+SMGLT+HCQ-A85

TAC+SMGLT-A2

Sham-2

TAC-100


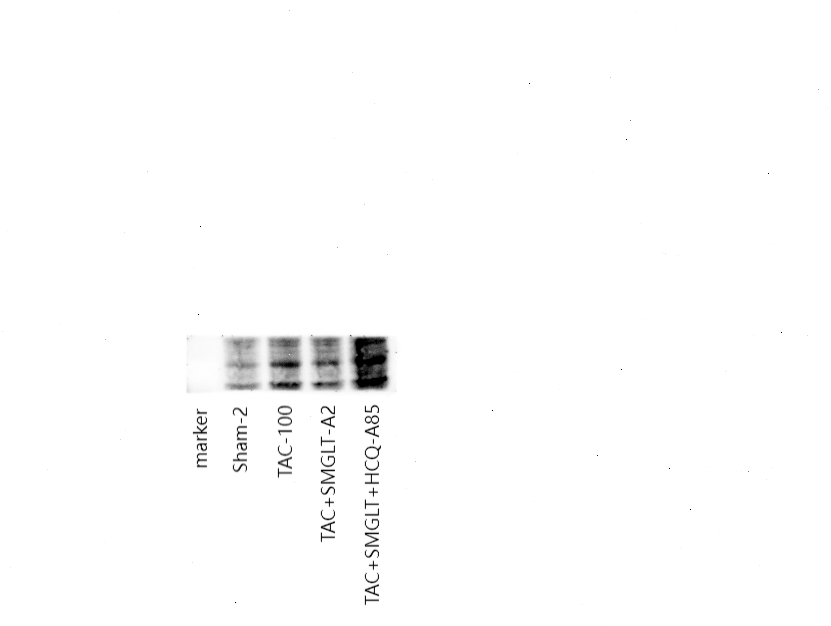


74KDa COX II


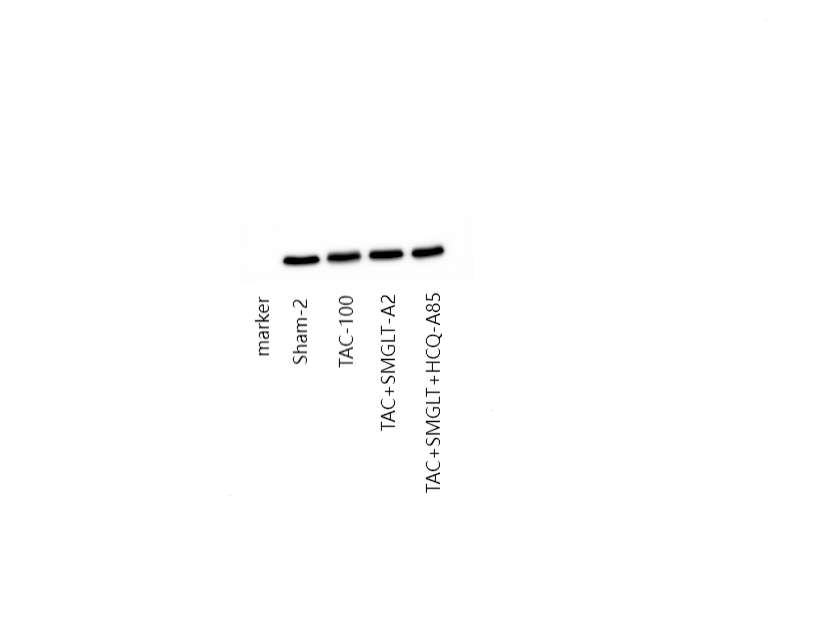


37KDa GAPDH

TAC+SMGLT+HCQ-A88

TAC-A3

TAC+SMGLT-A4

Sham-3


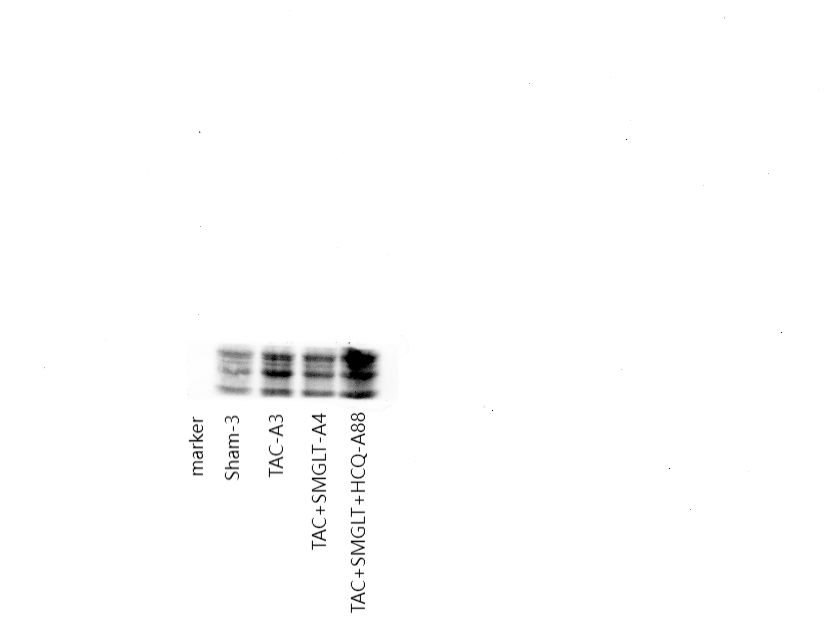


74KDa COX II


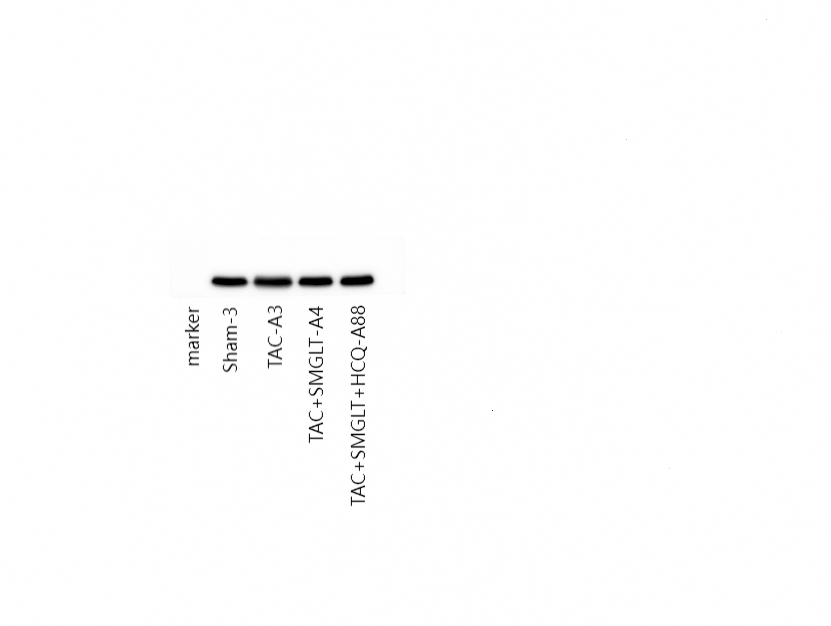


37KDa GAPDH

Sham-4

TAC-A6

TAC+SMGLT-5

TAC+SMGLT+HCQA92


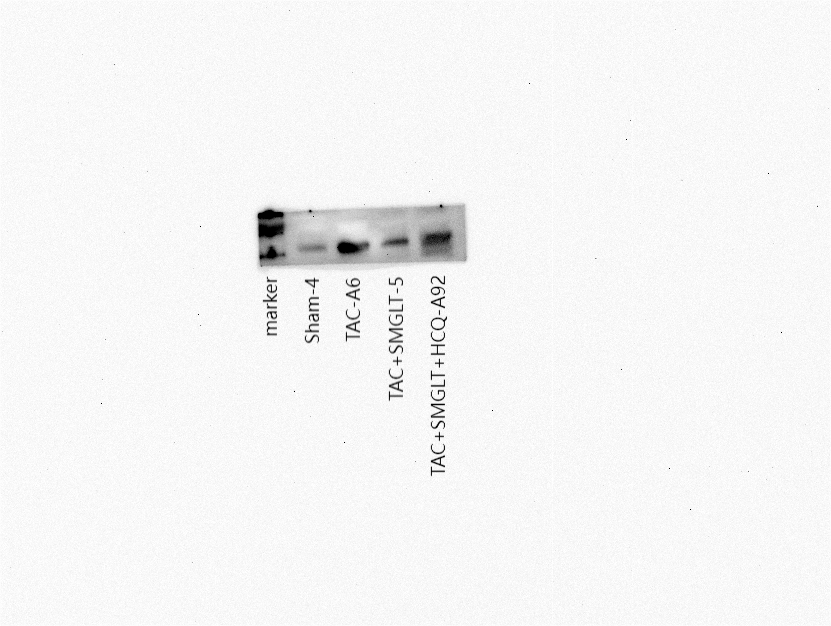


74KDa COX II

100KDa

70KDa


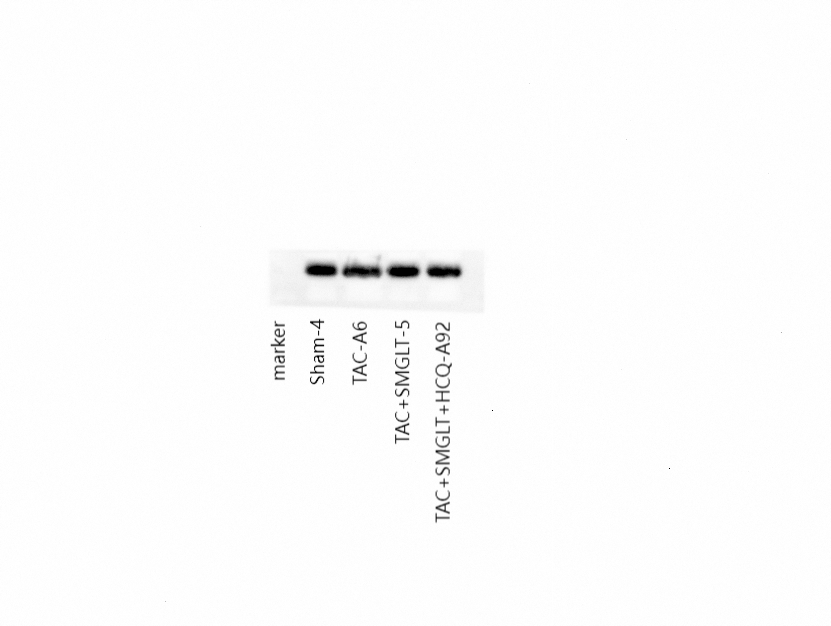


37KDa GAPDH

TAC+SMGLT+HCQ-A73

TAC+SMGLT-76

Sham-6

TAC-A36


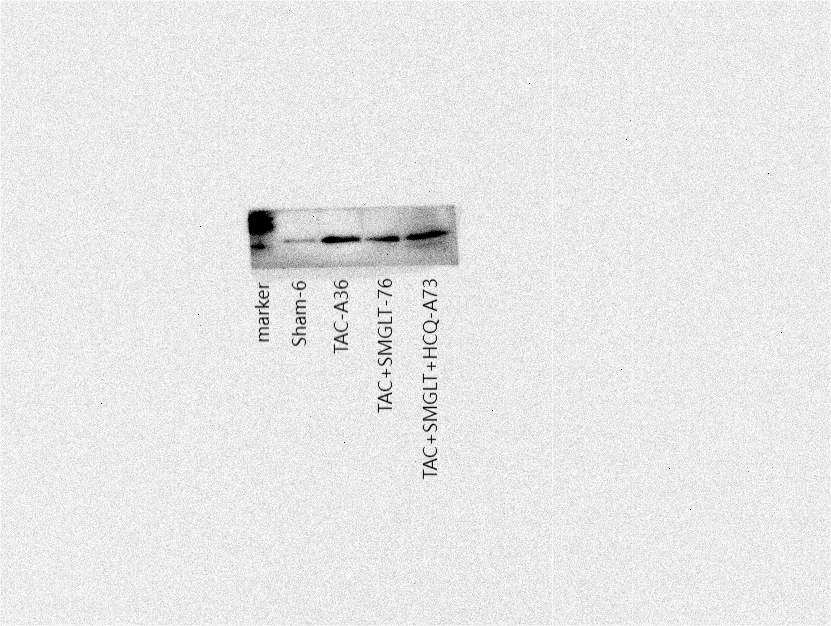


100KDa

70KDa

74KDa COX II


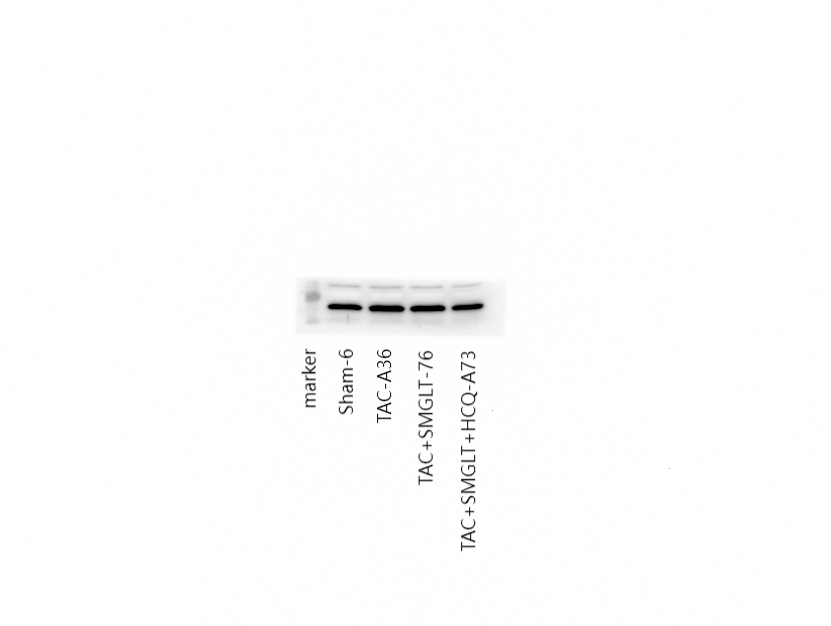


This figure of COXII was shown in our manuscript (Fig.2A).

40KDa

35KDa

37KDa GAPDH

TAC+SMGLT+HCQ-A84

TAC+SMGLT+HCQ-A84

TAC+SMGLT-A1

TAC+SMGLT-A1

Sham-1

TAC-3

Sham-1

TAC-3


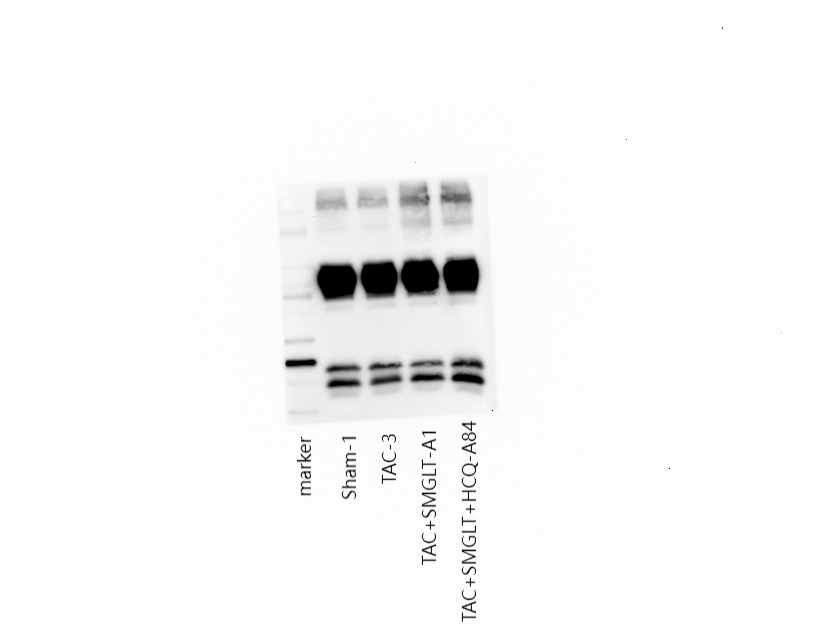

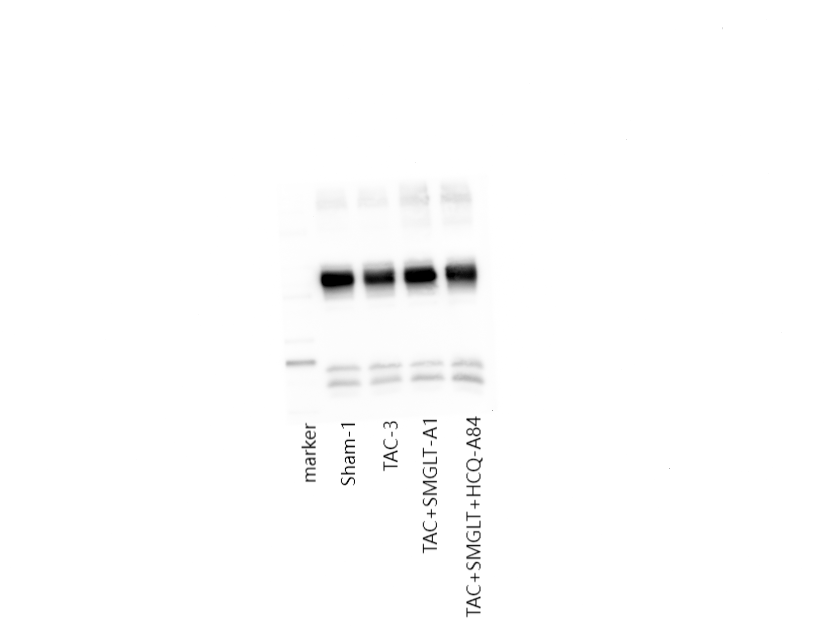


20KDa

20KDa

15KDa

10KDa

37KDa GAPDH

16/14KDa LC3B

Sham-2

TAC+SMGLT+HCQ-A85

TAC+SMGLT-A2

TAC-100


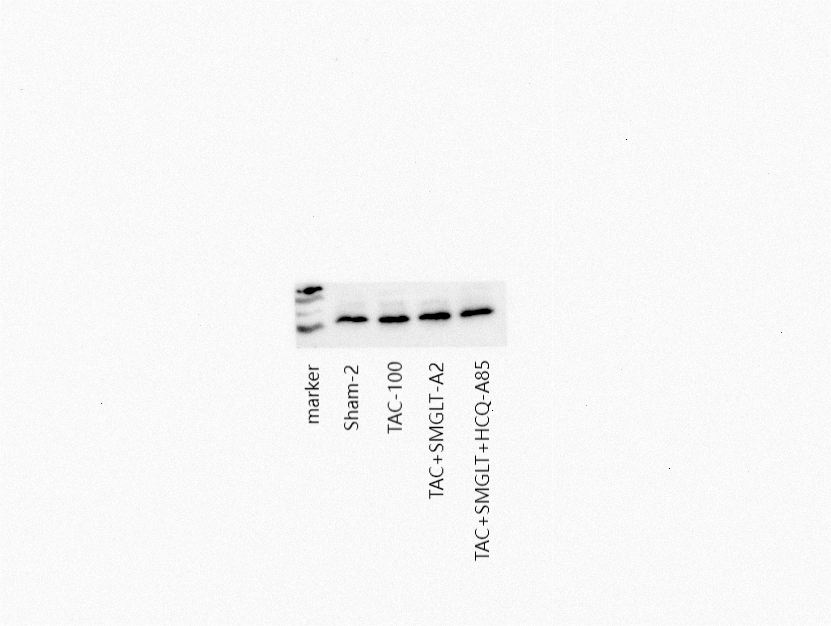


35KDa

37KDa GAPDH


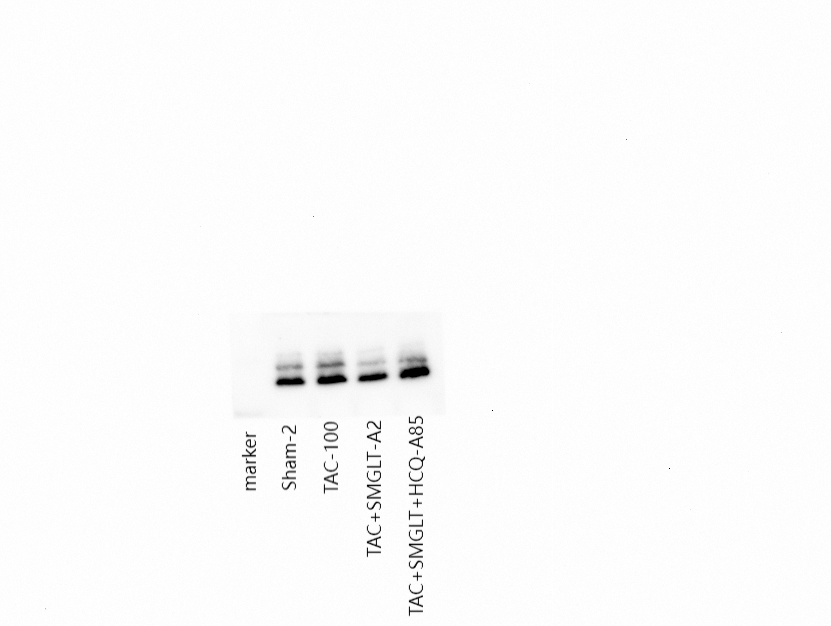


16/14KDa LC3B

TAC+SMGLT+HCQ-A88

TAC+SMGLT-A4


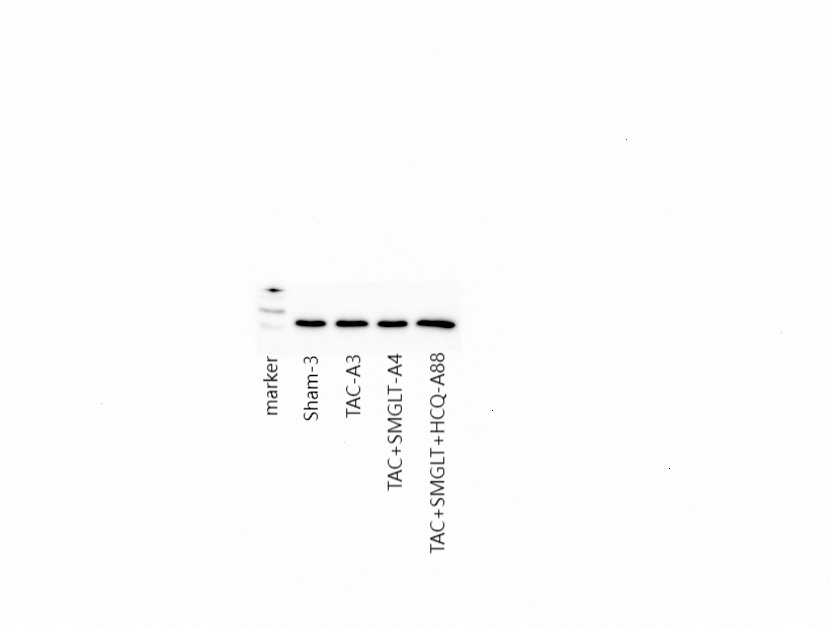


Sham-3

TAC-A3

40KDa

37KDa GAPDH


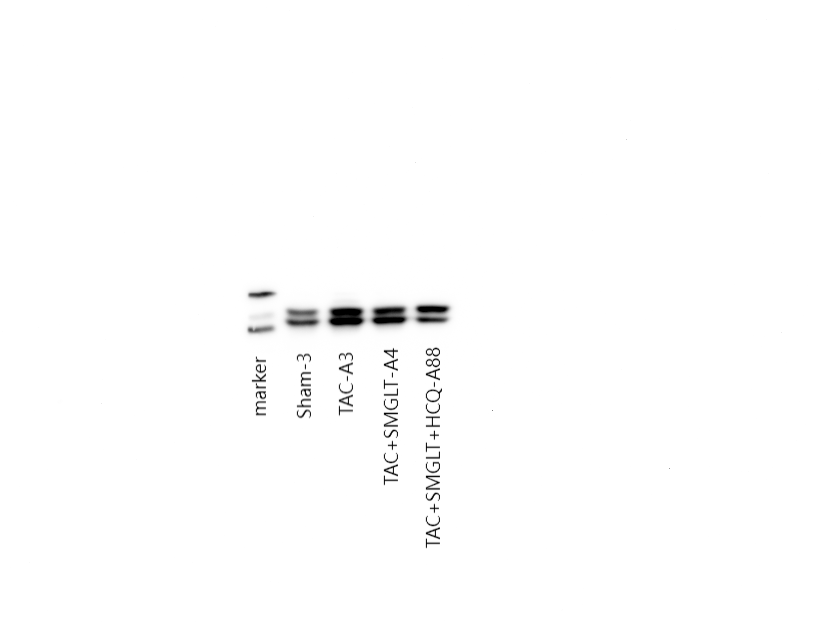


This figure of LC3B was shown in our manuscript (Fig. 2A).

20KDa

10KDa

16/14KDa LC3B

TAC+SMGLT+HCQ-A92

Sham-4

TAC-A6

TAC+SMGLT-5


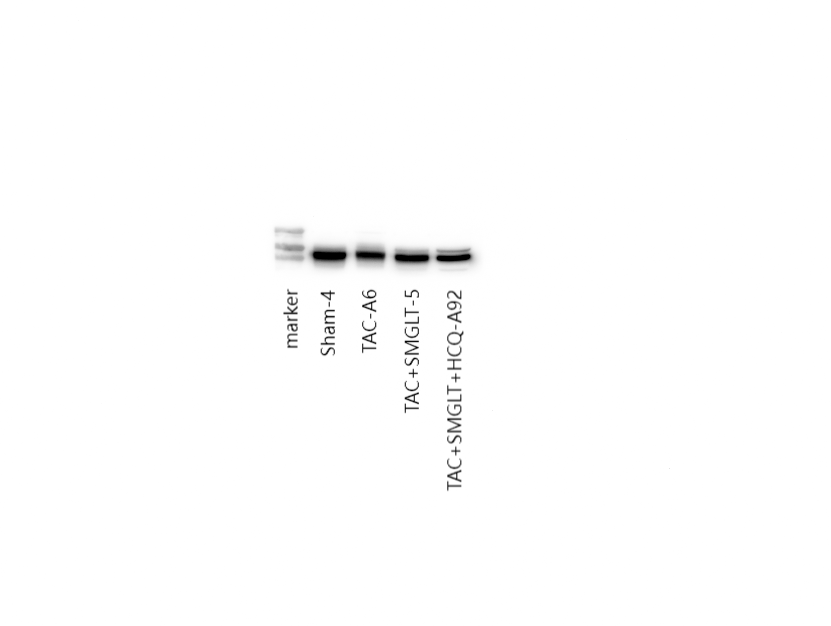


40KDa

35KDa

37KDa GAPDH


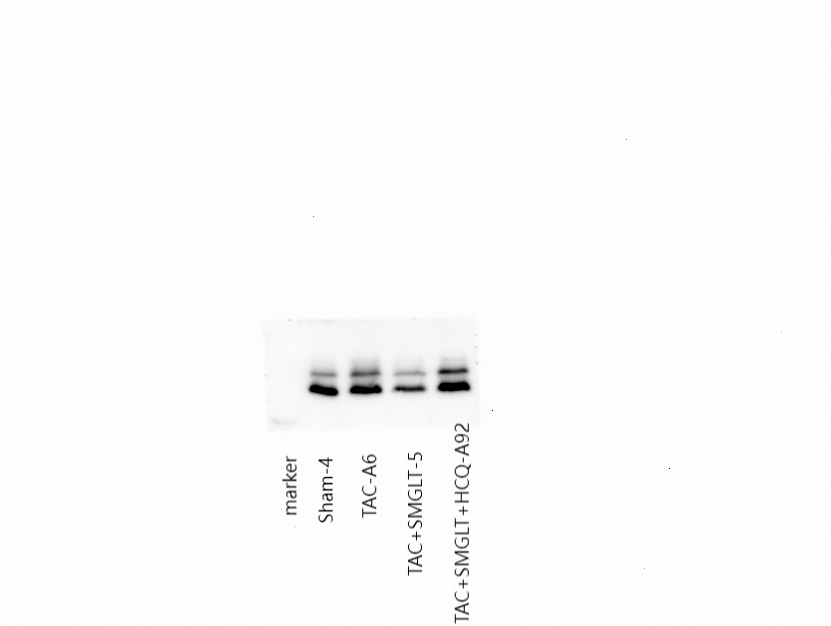


16/14KDa LC3B

Sham-5

TACA34

TAC+SMGLT-8

TAC+SMGLT+HCQ-A99


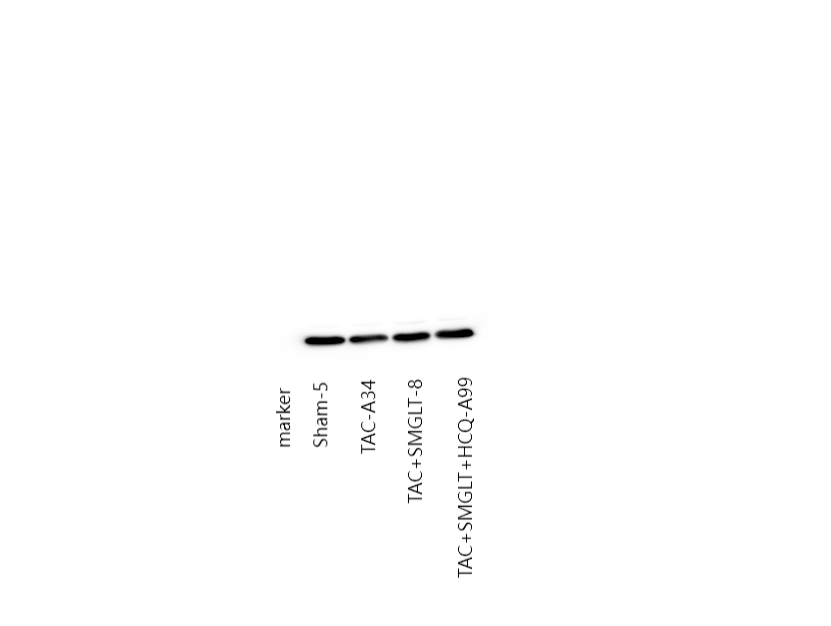


37KDa GAPDH


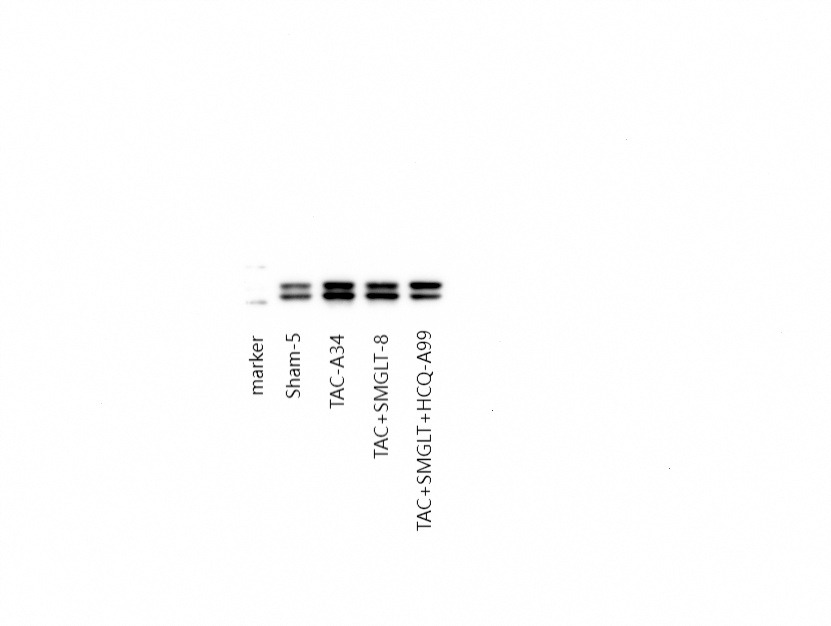


20KDa

10KDa

16/14KDa LC3B

TAC+SMGLT+HCQ-A73

TAC+SMGLT-76

TAC-A36

Sham-6


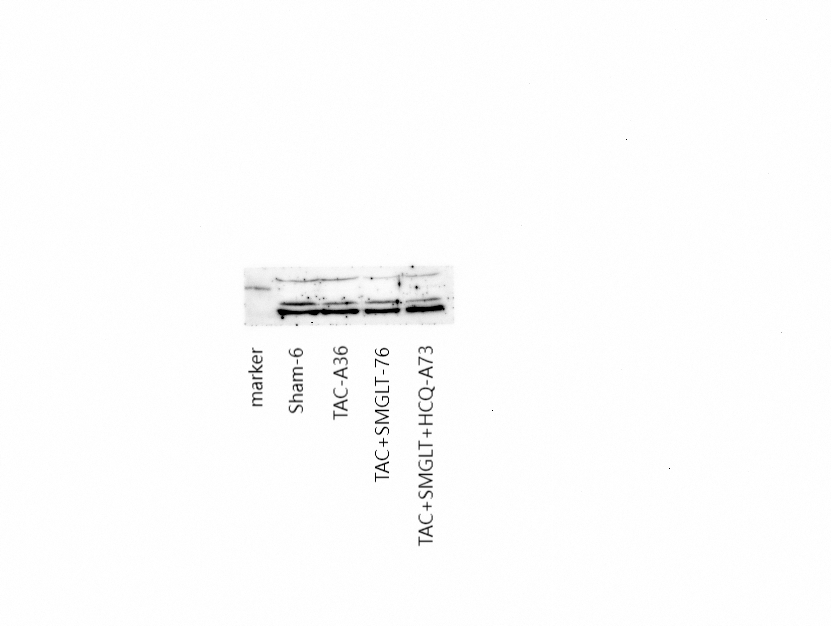


37KDa GAPDH

40KDa


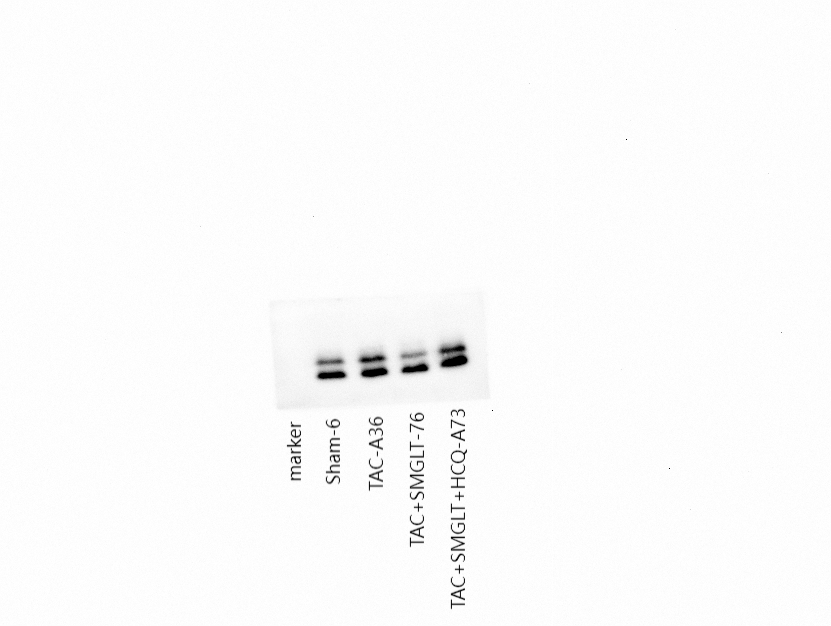


16/14KDa LC3B

TAC+SMGLT+HCQ-A84

TAC+SMGLT-A1

Sham-1

TAC-3


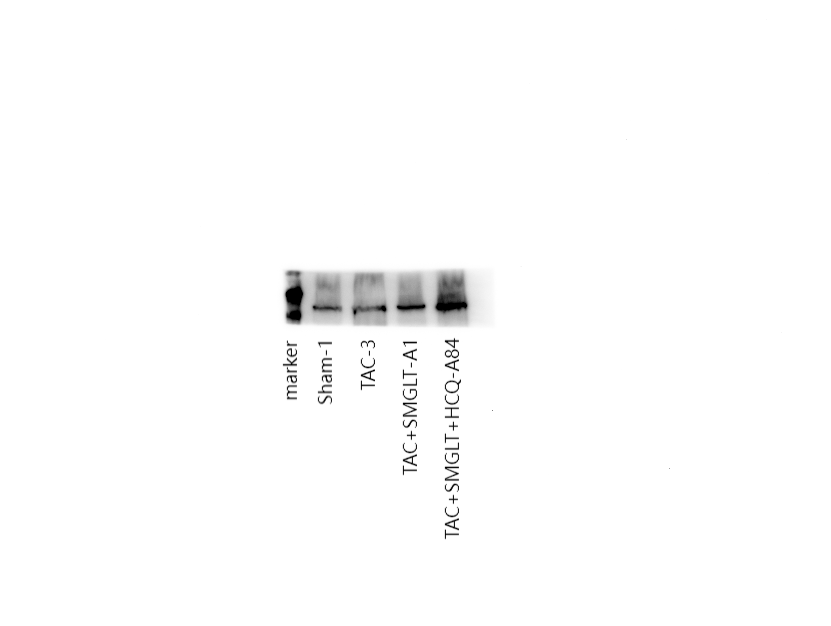


150KDa

100KDa

110KDa NLRP3


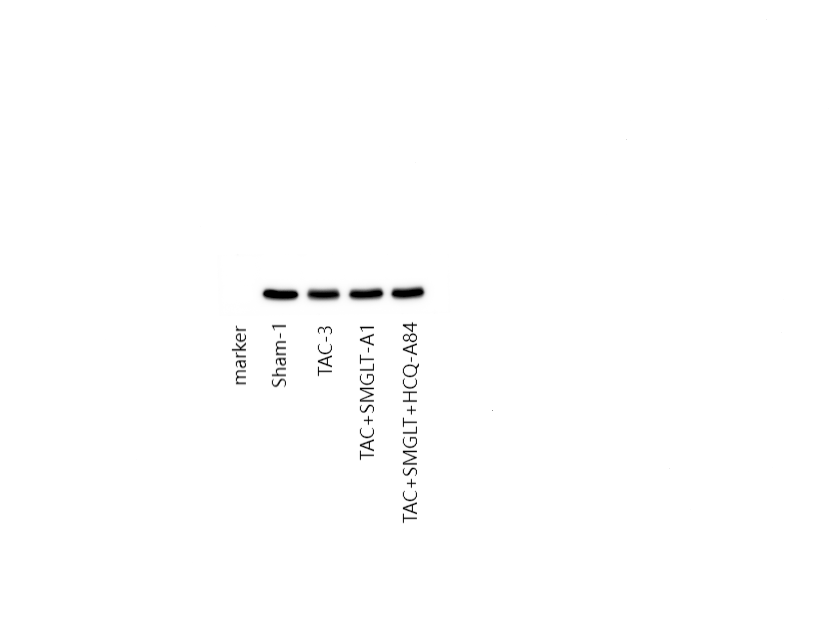


37KDa GAPDH

TAC+SMGLT-A2

TAC+SMGLT+HCQ-A85

Sham-2

TAC-100


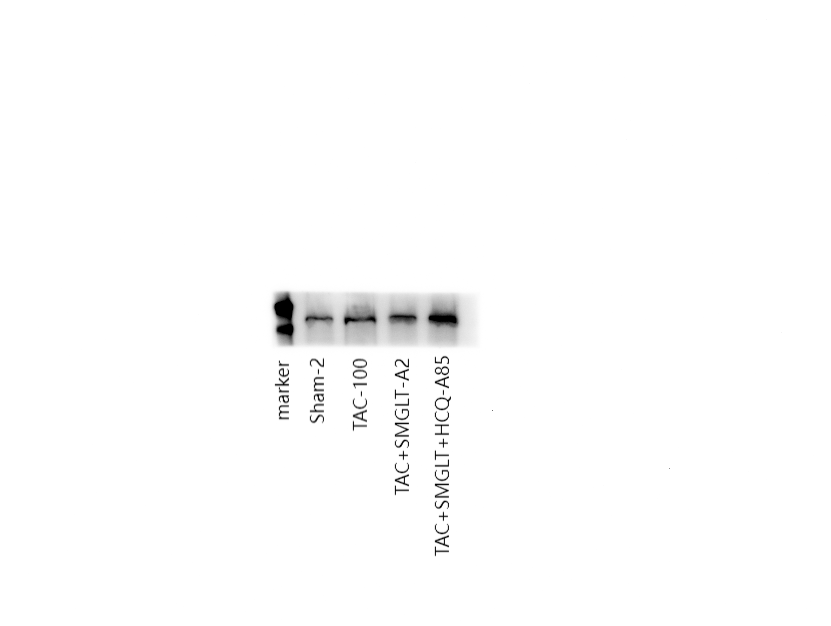


40KDa

35KDa

150KDa

100KDa

110KDa NLRP3


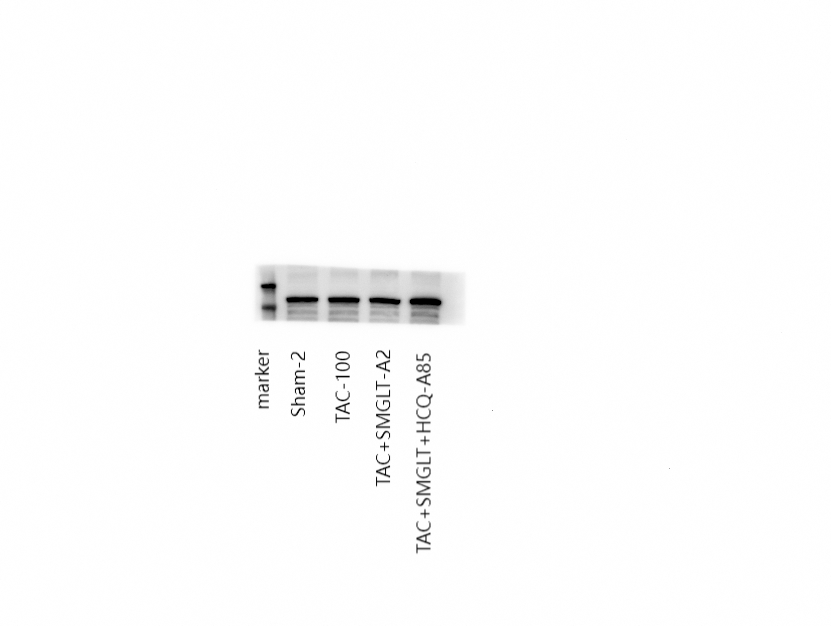


37KDa GAPDH

Sham-3

TAC+SMGLT-A4

TAC+SMGLT+HCQ-A88

TAC-A3

This figure of NLRP3 was shown in our manuscript (Fig. 3D).


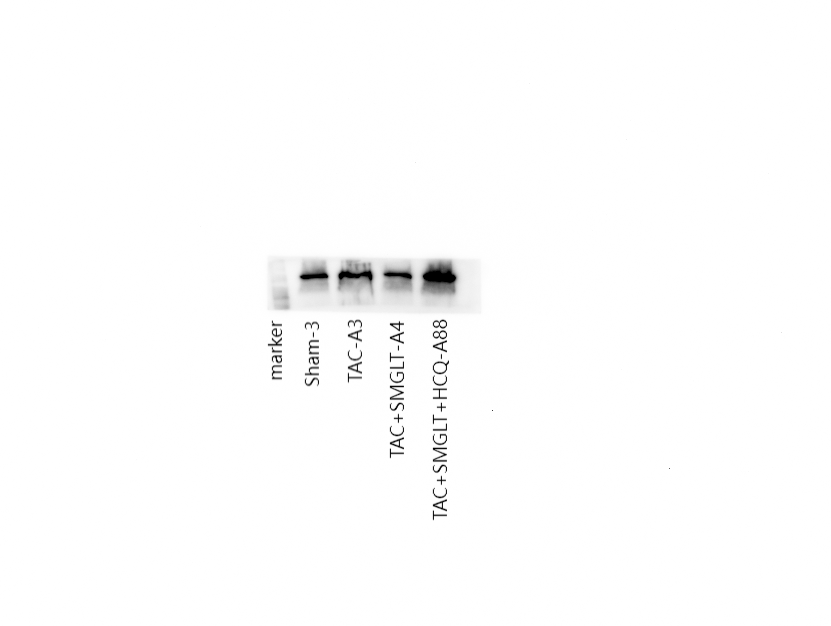


110KDa NLRP3


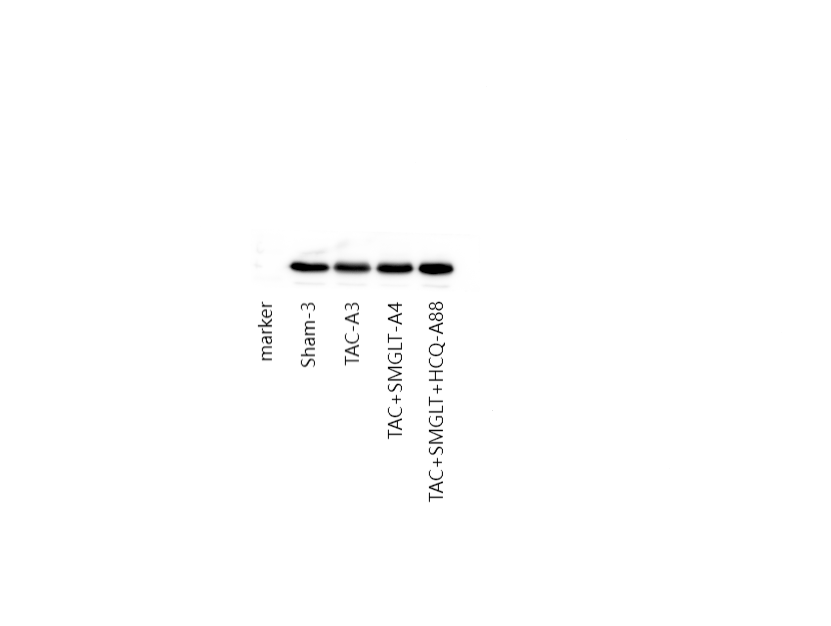


37KDa GAPDH


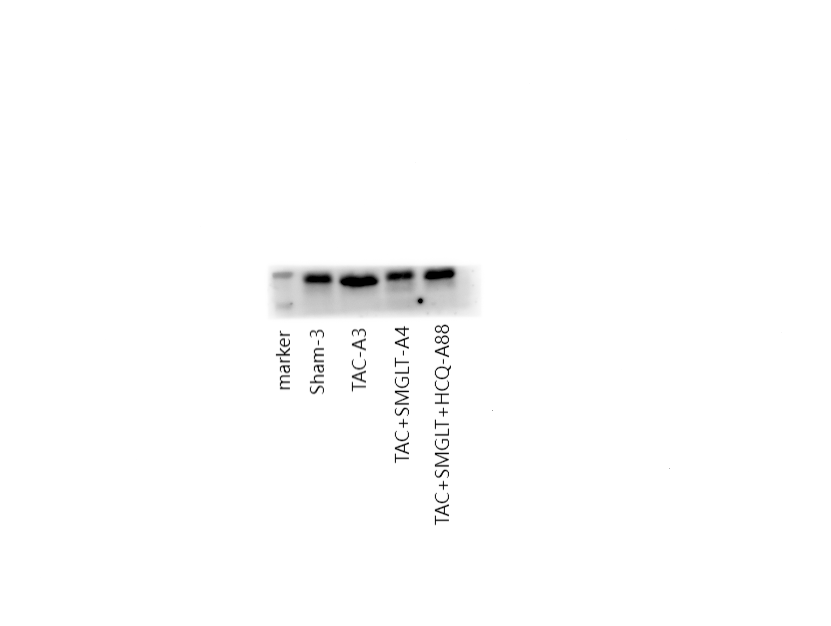


25KDa

20KDa

23KDa IL-18

Sham-4

TAC-A6

TAC+SMGLT-5

TAC+SMGLT+HCQ-A92


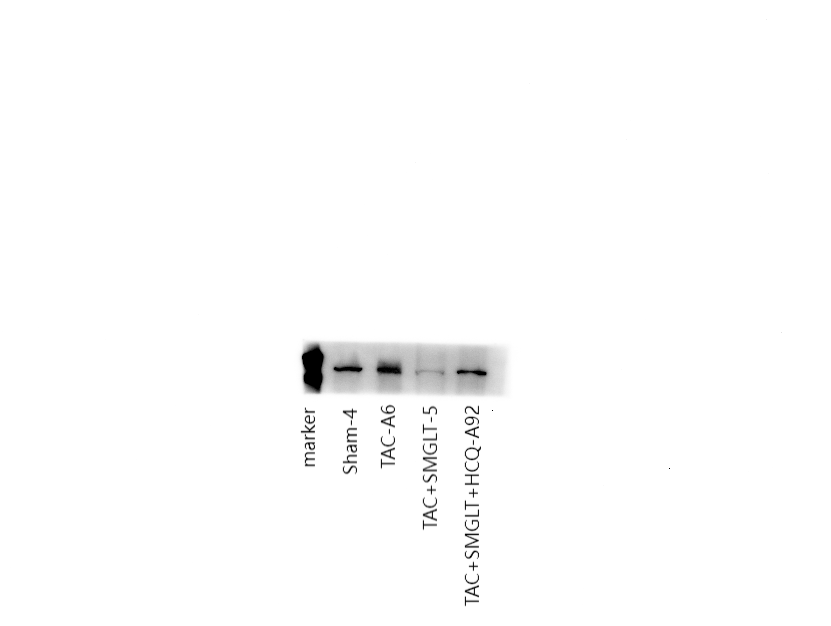


150KDa

100KDa

110KDa NLRP3


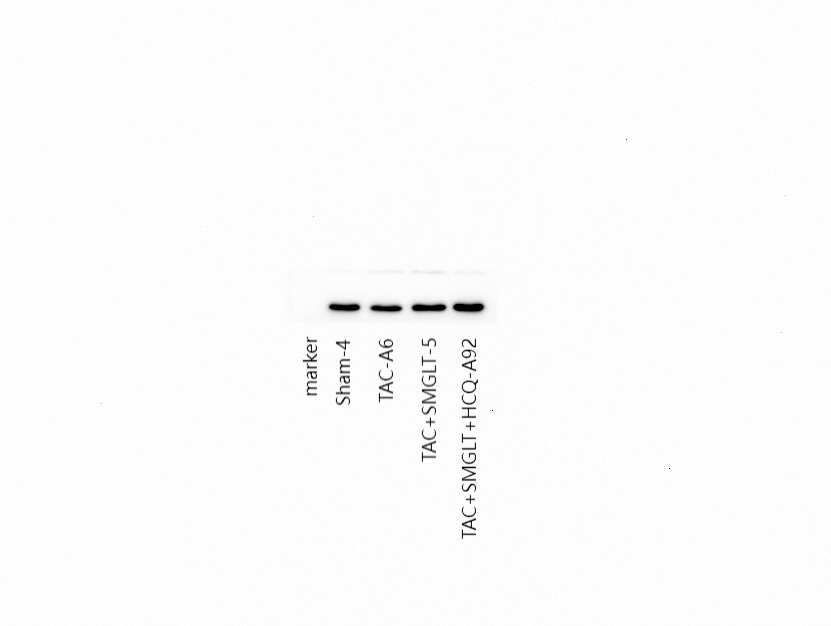


37KDa GAPDH


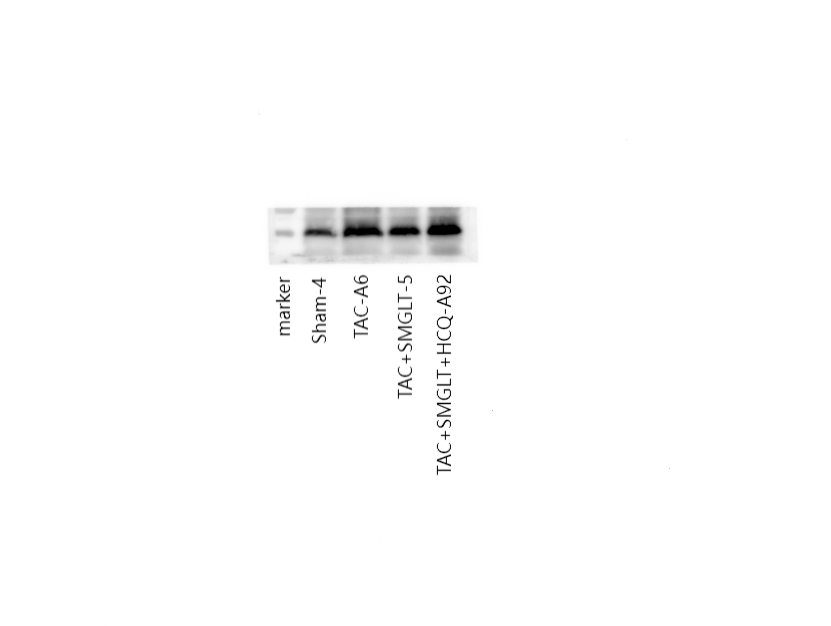


20KDa Caspase-1

20KDa

TAC+SMGLT+HCQ-A99

TAC+SMGLT-8

TAC-A34

Sham-5


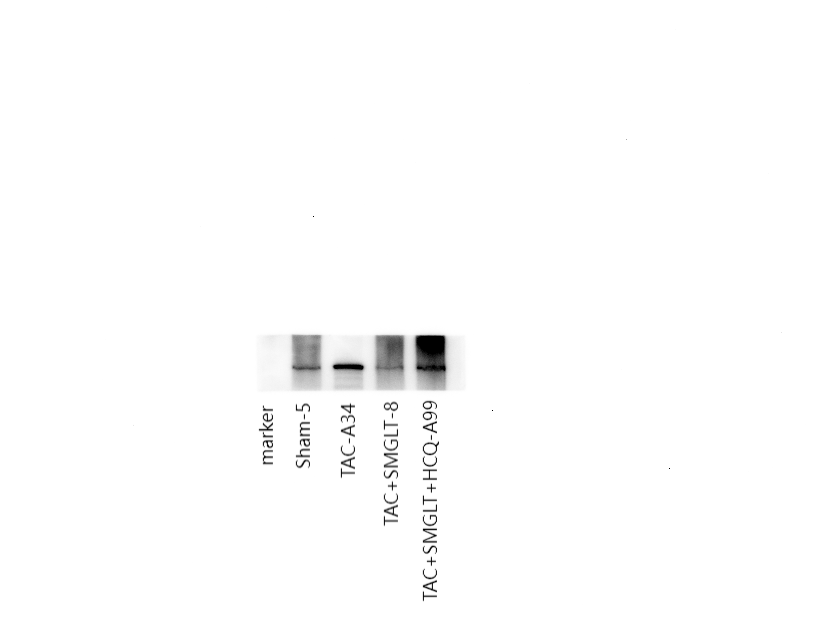


110KDa NLRP3

40KDa


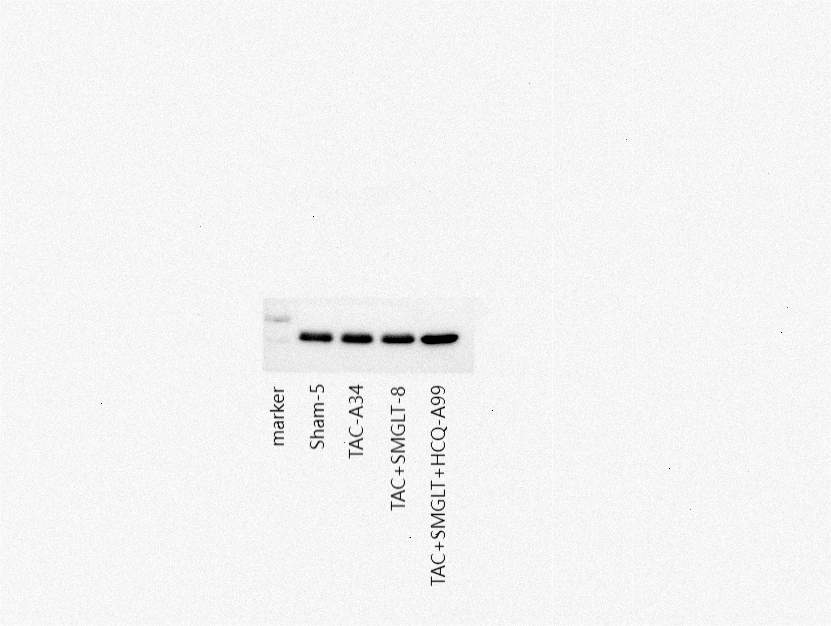


37KDa GAPDH

TAC+SMGLT+HCQA73

TAC+SMGLT-76

TAC-A36

Sham-6


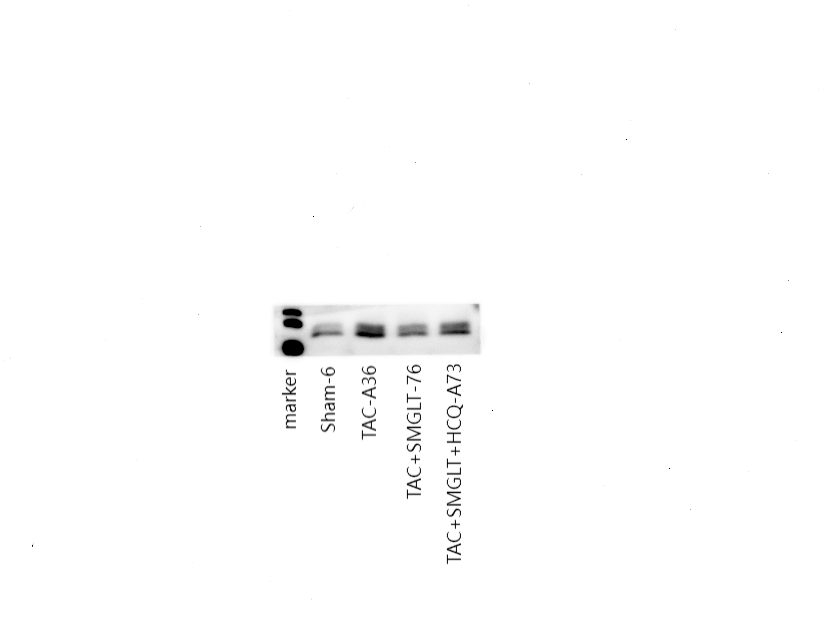


150KDa

100KDa

110KDa NLRP3


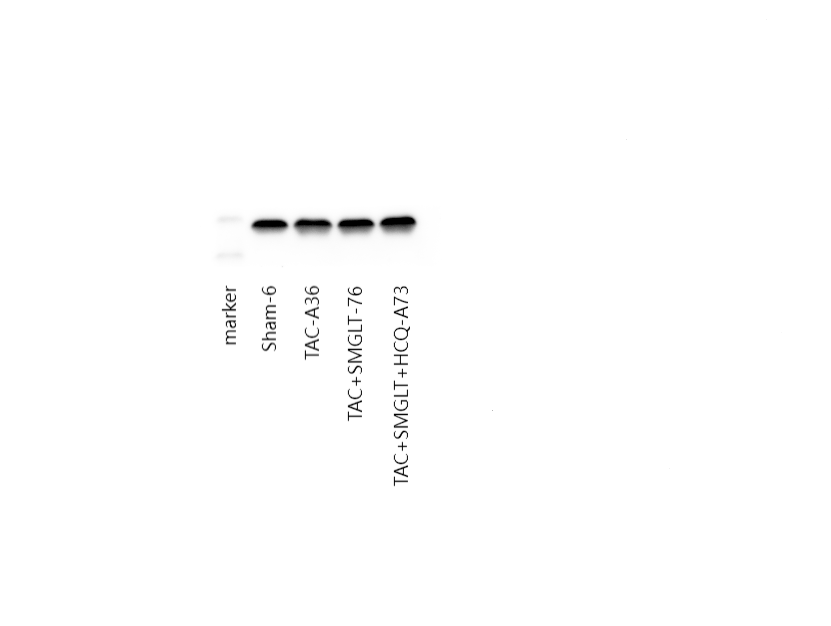


40KDa

35KDa

37KDa GAPDH


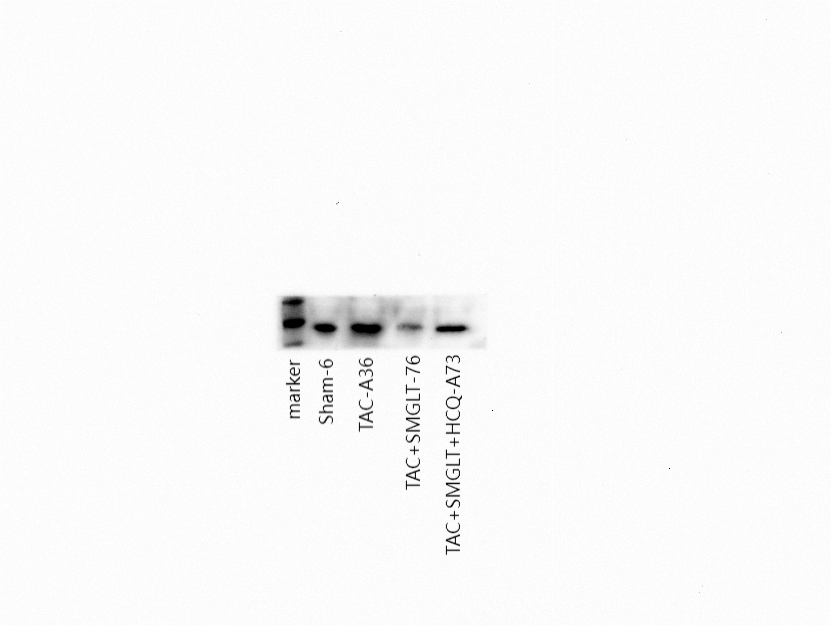


25KDa

23KDa IL-18

TAC+SMGLT+HCQ-A84

TAC+SMGLT-A1

Sham-1

TAC-3


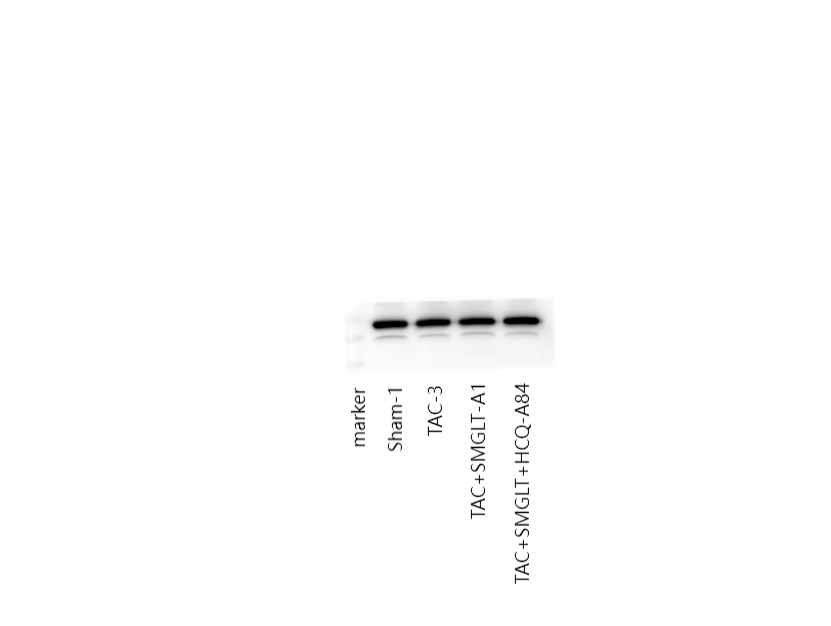

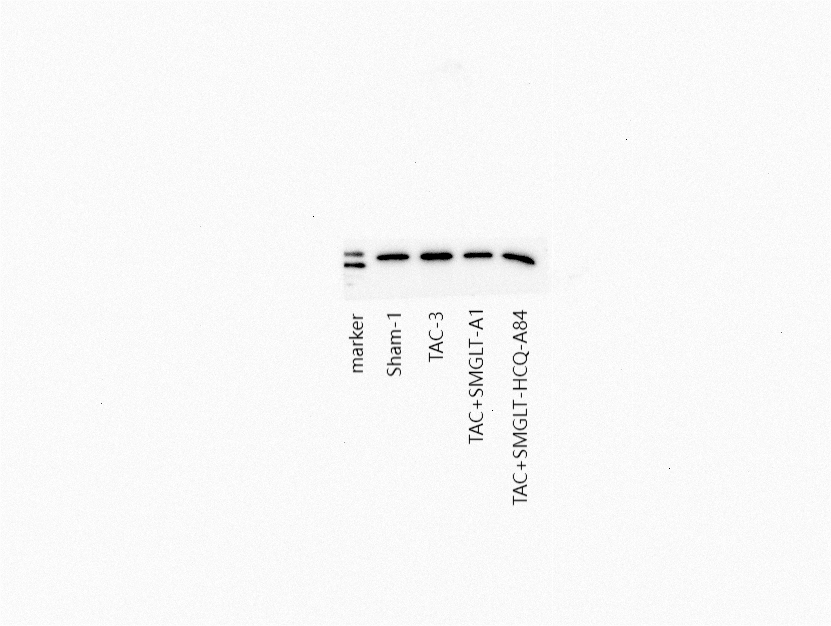


20KDa

37KDa GAPDH

20KDa Caspase-1

TAC+SMGLT-A2

TAC+SMGLT+HCQ-A85

Sham-2

TAC-100


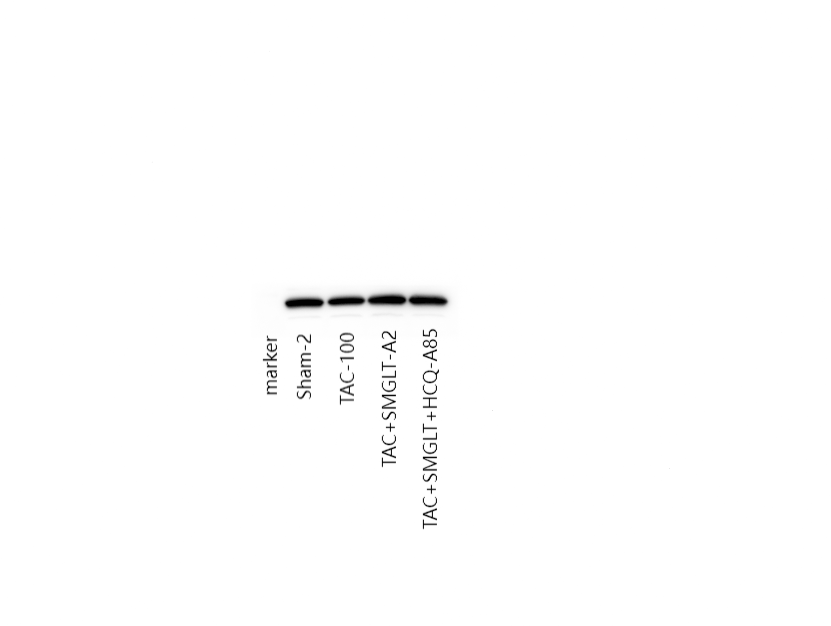


37KDa GAPDH


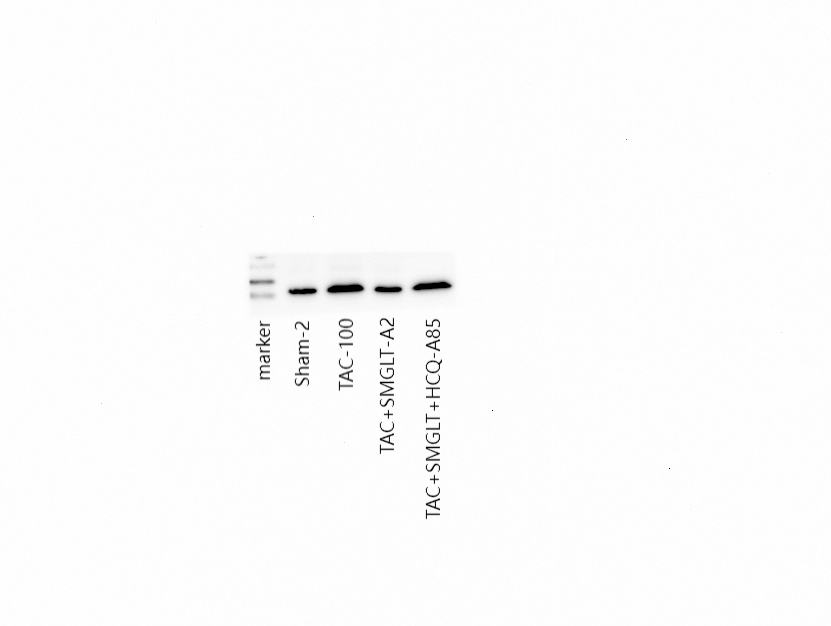


20KDa

20KDa Caspase-1

TAC+SMGLT+HCQ-A88

TAC+SMGLT-A4

TAC-A3

Sham-3


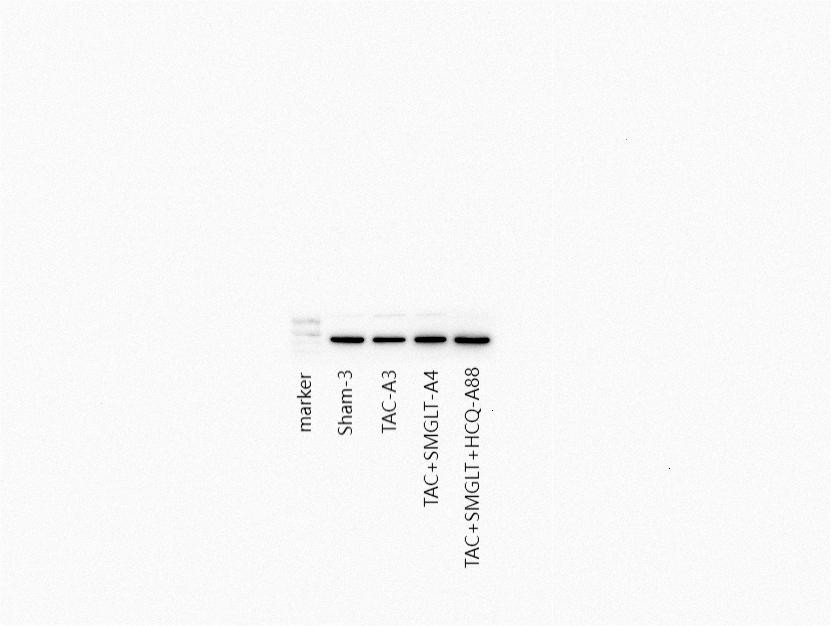


40KDa

37KDa GAPDH


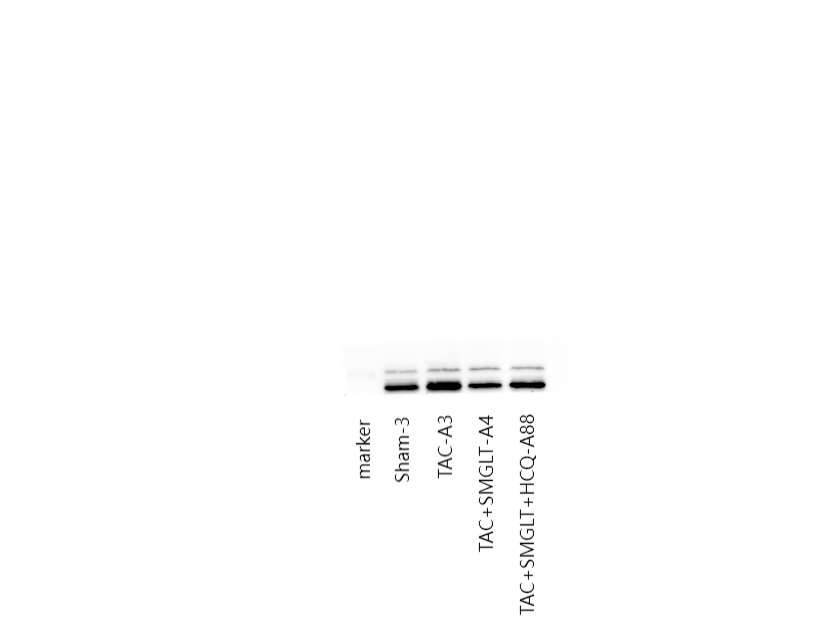


This figure of Caspase-1 was shown in our manuscript (Fig. 3D).

20KDa Caspase-1

TAC+SMGLT+HCQ-A99

TAC+SMGLT-8

TAC-A34

Sham-5


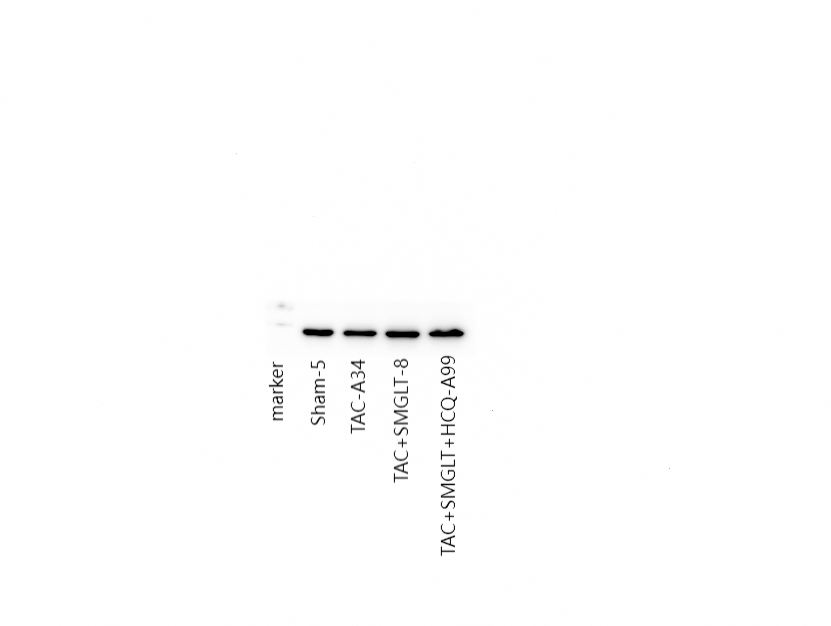


50KDa

40KDa

37KDa GAPDH


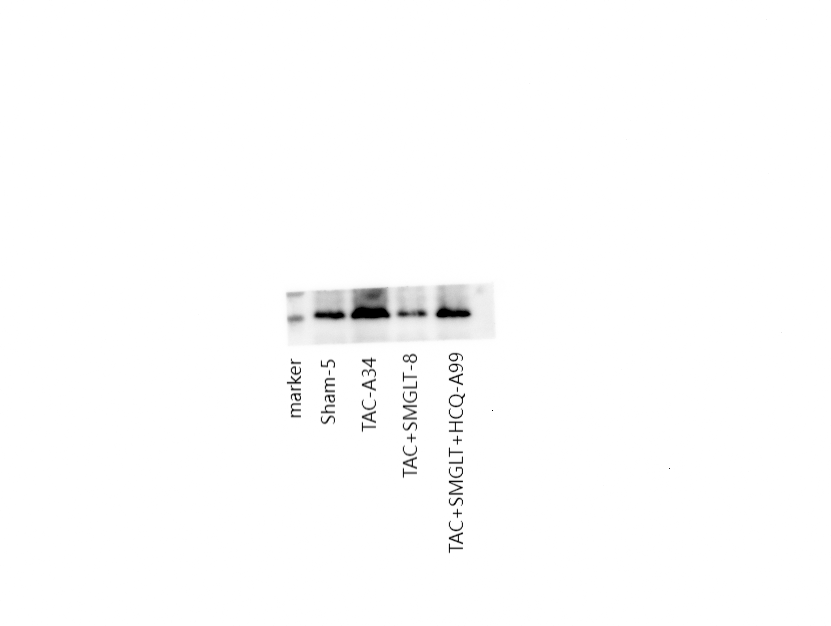


20KDa

20KDa Caspase-1

TAC+SMGLT+HCQ-A73

TAC+SMGLT-76

TAC-A36

Sham-6


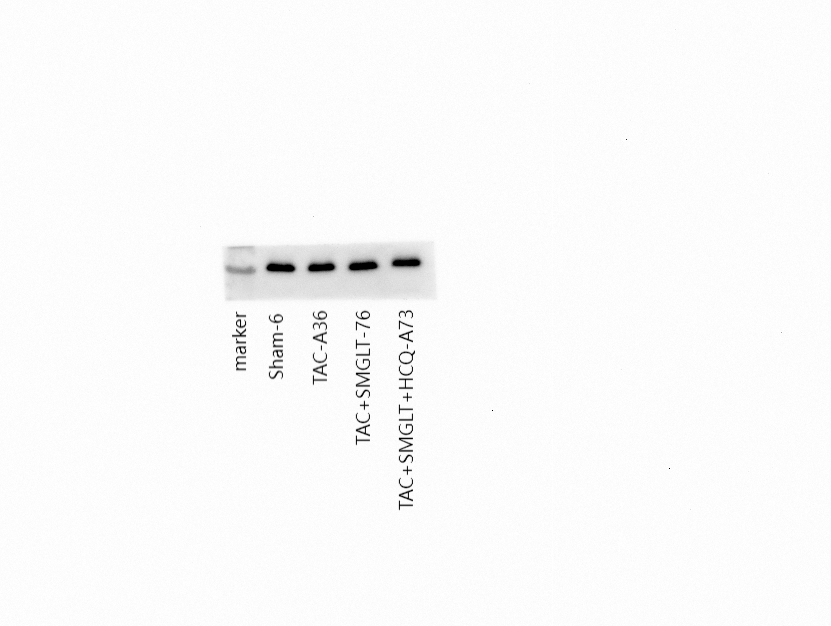


37KDa GAPDH

35KDa


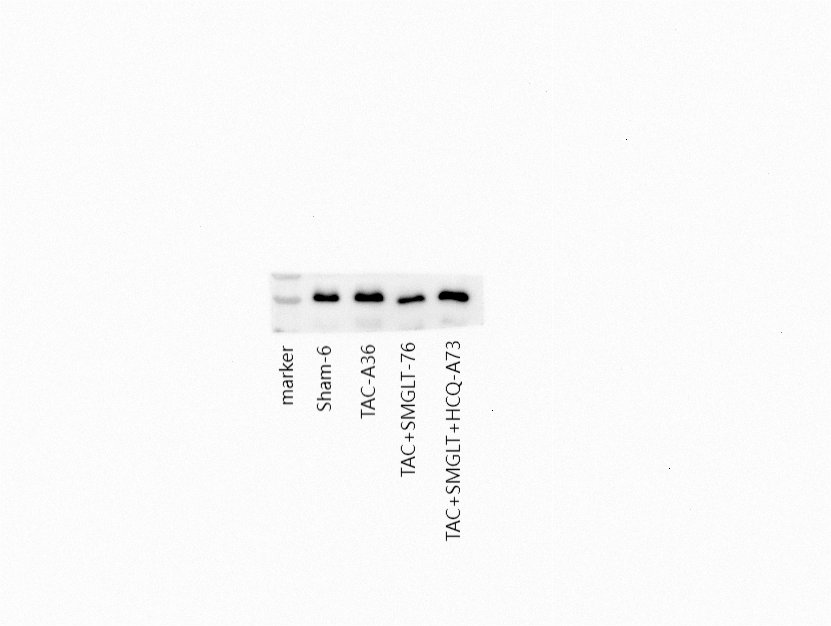


20KDa

20KDa Caspase-1

TAC+SMGLT+HCQ-A84

TAC+SMGLT-A1

Sham-1

TAC-3


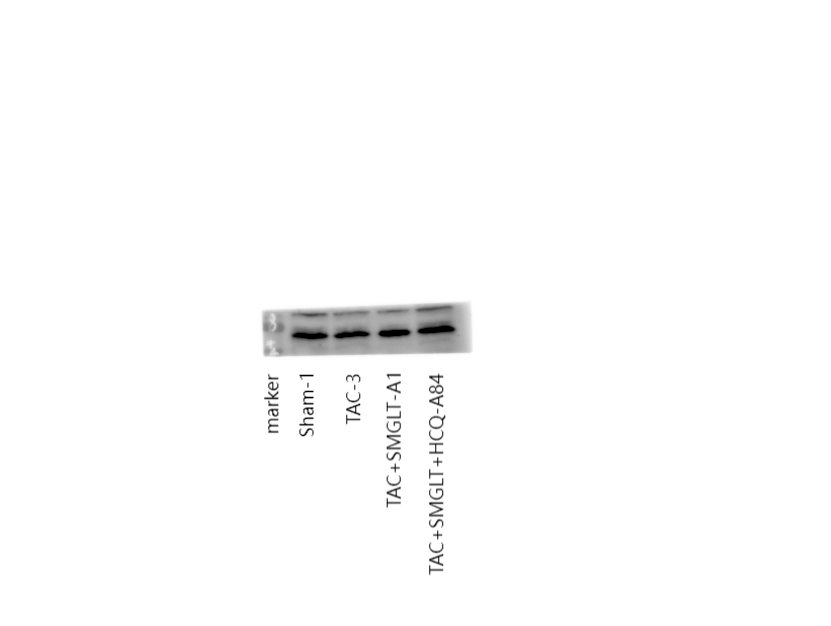


40KDa

37KDa GAPDH


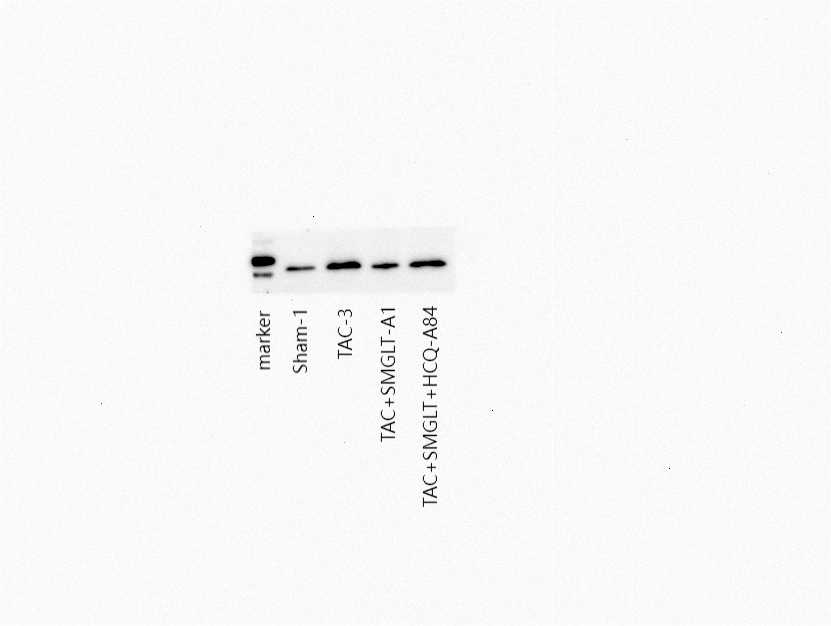


25KDa

23KDa IL-18

TAC+SMGLT+HCQ-A85

TAC+SMGLT-A2

TAC-100

Sham-2


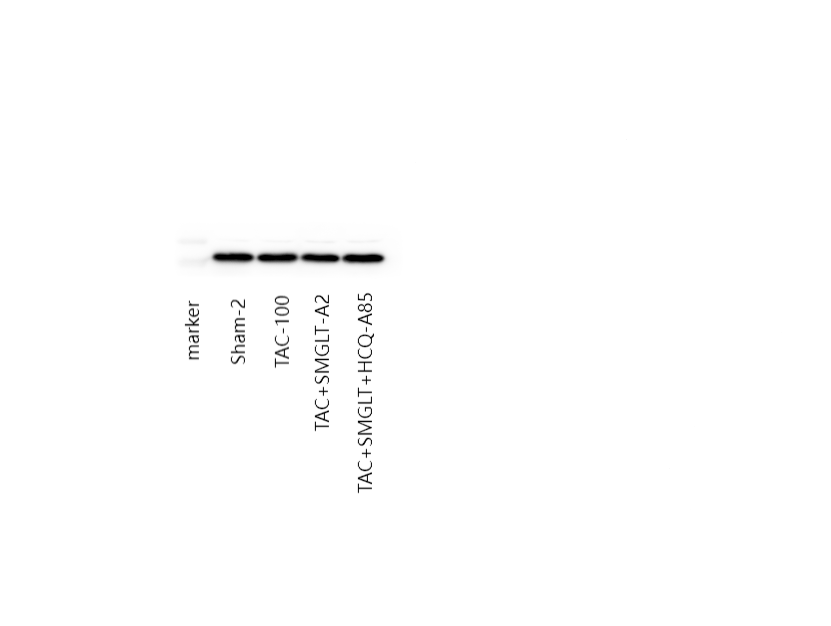


37KDa GAPDH

40KDa

35KDa


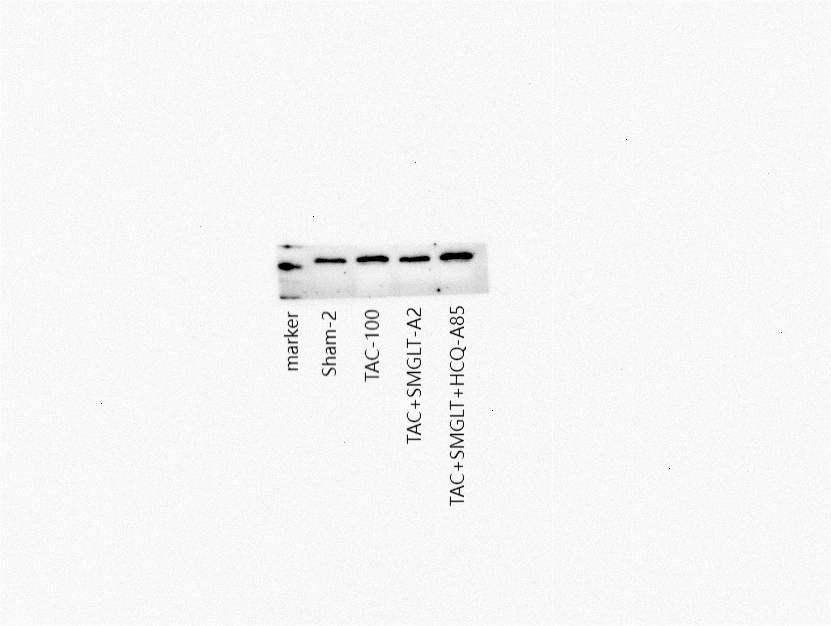


20KDa

23KDa IL-18

TAC+SMGLT+HCQ-A92

TAC+SMGLT-5

Sham-4

TAC-A6


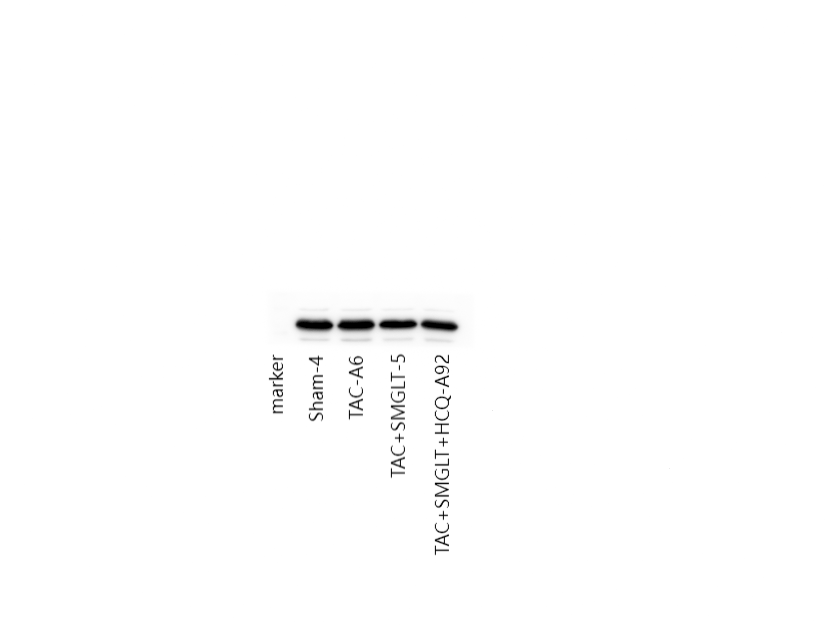


37KDa GAPDH


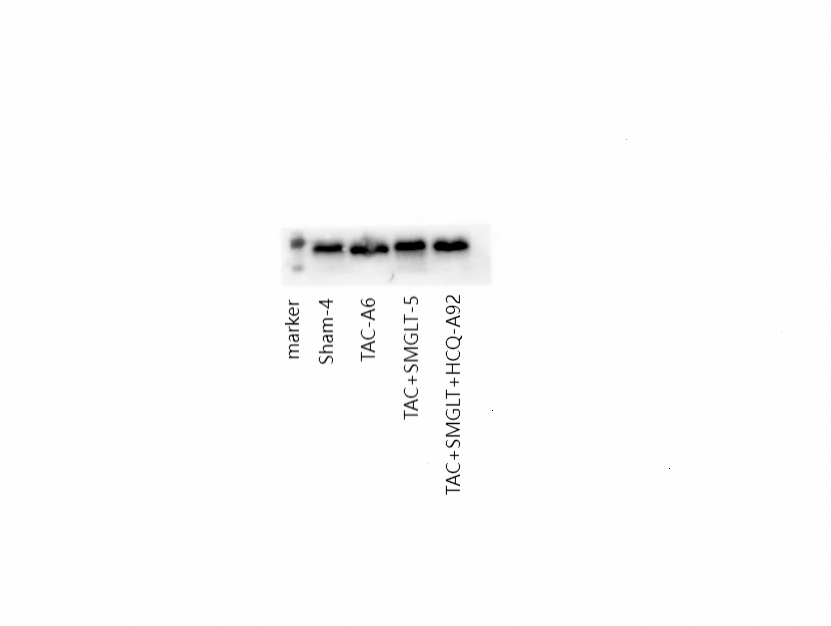


25KDa

23KDa IL-18

TAC+SMGLT+HCQ-A99

TAC+SMGLT-8

TAC-A34


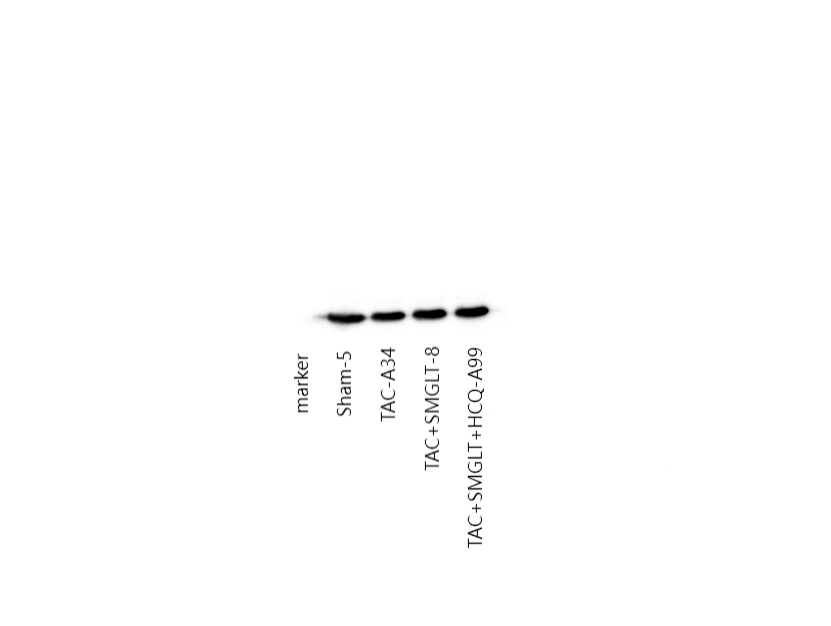


This figure of IL-18 was shown in our manuscript (Fig. 3D).

Sham-5

37KDa GAPDH


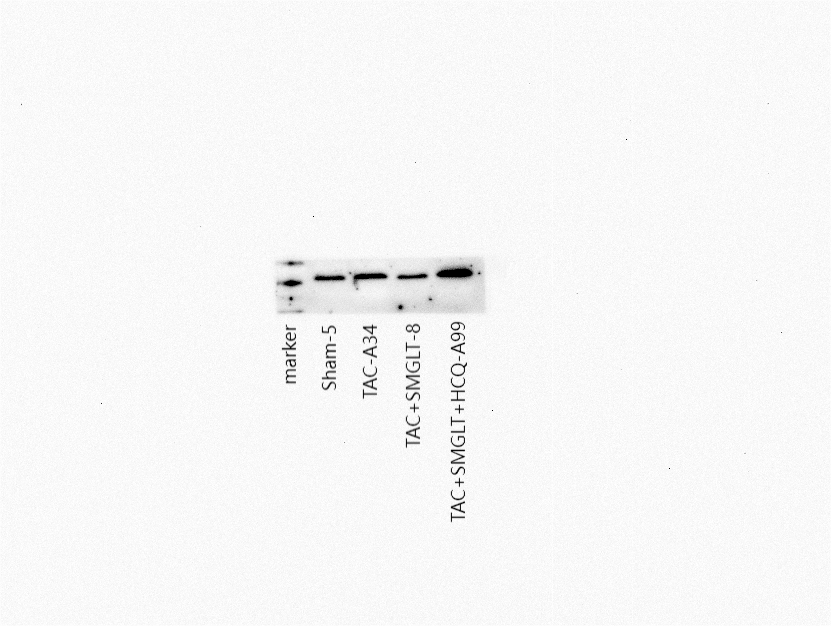


20KDa

23KDa IL-18
